# Core remodeling leads to long wavelength fluoro-coumarins†

**DOI:** 10.1039/d0sc02566f

**Published:** 2020-07-03

**Authors:** Siddharth S. Matikonda, Joseph Ivanic, Miguel Gomez, Gabrielle Hammersley, Martin J. Schnermann

**Affiliations:** Chemical Biology Laboratory, Center for Cancer Research, National Cancer Institute, National Institutes of Health Frederick Maryland 21702 USA martin.schnermann@nih.gov; Advanced Biomedical Computational Science, Frederick National Laboratory for Cancer Research Frederick Maryland 21702 USA

## Abstract

Low molecular weight, uncharged far-red and NIR dyes would be enabling for a range of imaging applications. Rational redesign of the coumarin scaffold leads to Fluoro-Coumarins (FCs), the lowest molecular weight dyes with emission maxima beyond 700, 800, and 900 nm. FCs display large Stokes shifts and high environmental sensitivity, with a 40-fold increase in emission intensity in hydrophobic solvents. Untargeted variants exhibit selective lipid droplet and nuclear staining in live cells. Furthermore, sulfo-lipid derivatization enables active targeting to the plasma membrane. Overall, these studies report a promising platform for the development of biocompatible, context-responsive imaging agents.

## Abstract 2

Fluoro-Coumarins are a novel class of far-red and near-infrared solvent sensitive dyes of exceptionally low molecular weight.

## Graphical abstract

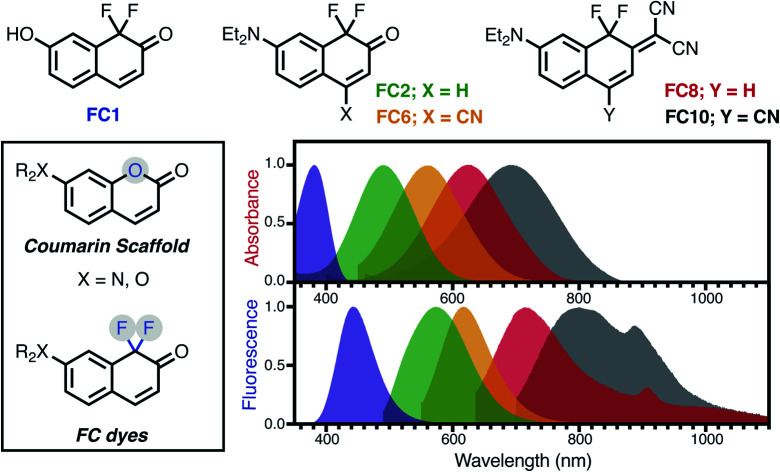

## Introduction

Fluorescent small molecules are foundational elements of biological experimentation.^1^ There remains a significant need for improved variants in the biologically compatible far-red and near-infrared (NIR) range.^2,3^ In particular, low molecular weight, uncharged NIR scaffolds are needed for contexts (*e.g.* intracellular settings) where the poor physical properties of existing chromophores are problematic.^4,5^ The most established bathochromic-shifting strategy involves extending the π-system, leading to an increase in molecular weight. It is also possible to tune chromophore electronics by introducing functional groups that reduce the HOMO → LUMO gap. While this approach has been applied fruitfully with rhodamine, fluorescein, and BODIPY derivatives,^6–17^ it had not been applied on coumarins, an important low molecular weight dye scaffold.

**Fig. 1:**
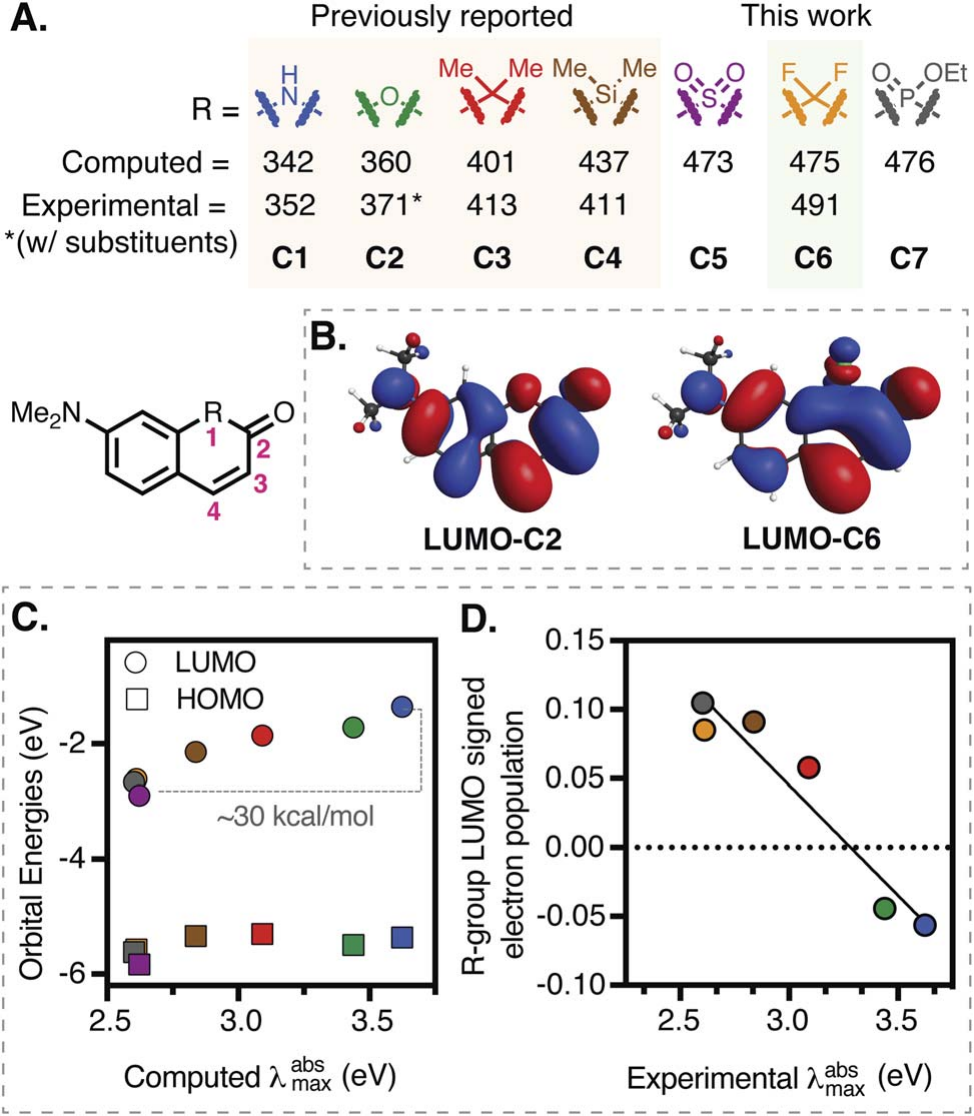
(A) Computational and experimental data for various substituents (**C1–C7**) at **R**-position on a 7-dimethylamino-coumarin-derived scaffold. (B) Comparison between the LUMOs of **C2** and **C6**. (C) Correlation between the computed *λ*^abs^_max_ (TDDFT-B3LYP-PCM (H_2_O)) and HOMO/LUMO orbital energies. (D) Correlation (*R*^2^ = 0.92) between signed R-group Mulliken population in LUMO and experimental *λ*^abs^_max_ (see ESI for full details†).

Coumarins are extensively employed due to excellent cell permeability, low toxicity, and significant utility as “turn-on” probes.^18,19^ Previous efforts to red-shift these molecules involved (1) replacing the carbonyl group at –2 position with electron withdrawing groups,^20–23^ (2) improving the electron donating ability of the aniline at –7 position,^24,25^ and (3) extending the conjugation at –3 position.^26–28^ These approaches append bulky moieties onto the coumarin core, significantly increasing molecular weight. Recent attempts to red-shift these molecules through modifications to the –1 position include dimethyl-methylene and dimethyl-silyl groups, which led to only modest effects on the absorbance maxima (Fig. 1A).^29,30^ The installation of electron deficient substituents at this position has not been explored previously, which is notable given the dramatic effects of electron-poor functional groups at corresponding positions on other scaffolds.^6–17^ Indeed, the computational studies detailed below suggest electron withdrawing substituents should result in dramatic bathochromic shifts. Driving these studies, and unexplored in any common fluorophore class, is the low molecular weight difluoro-methylene (CF_2_) substituent.

Here we detail the preparation and evaluation of the first series of Fluoro-Coumarin (FC) dyes. Installation of the key difluoro functionality is achieved by a high-yielding regioselective difluorination reaction. Building on this chemistry, we prepare a series of compounds that span the far-red to NIR range. Notably, FCs, especially the NIR donor–acceptor merocyanine variants, exhibit high solvent sensitivity with excellent quantum yields (*Φ*_F_) in hydrophobic environments. When applied to live-cell imaging, several untargeted variants exhibit high organelle specificity, with derivatives localizing selectively to lipid droplets and the nucleus. Furthermore, sulfo-lipid derivatization provides a NIR dye for use in selective plasma membrane labeling. Overall, FCs represent a new class of low molecular weight far-red and NIR dyes with significant potential for use as responsive chromophores.

## Results and discussion

### Computational design

Replacement of the ether oxygen atom on the rhodamine and fluorescein scaffold with electron withdrawing groups leads to significant bathochromic shifts.^6–14^ We hypothesized the same logic might apply to the coumarin scaffold, and computationally examined established and related replacements at the ring-embedded −1 oxygen atom (Fig. 1A). Unlike other cases where higher level methods were required,^31,32^ time-dependent density functional theory (TDDFT) with the B3LYP functional and water solvent modeling provided computed absorbance energies in good agreement with the experimental values for previously known compounds **C1–C4** (Fig. 1A and S16, Table S3†).^29,30,33^ Most notably, difluoro-methylene (**C6**), with a predicted *λ*^abs^_max_ of 475 nm, was essentially identical to that of sulfone (**C5**, 473 nm) and phosphinate (**C7**, 476 nm) derivatives. We chose to pursue the fluorocoumarins as we hypothesized these might be particularly straightforward to access, while having moderately lower molecular weights and total polar surface areas (Table S5†).

Additional analysis of the computational data suggests a principal role for LUMO effects – computed HOMO energies are similar across the series (varying by 0.47 eV), while LUMO energies decrease by nearly 1.54 eV (Fig. 1C and Section S7†). Along these lines, inspection of the frontier molecular orbitals of **C1–C7** revealed nearly constant HOMO structures, but significantly different LUMO configurations (Fig. 1B and S17†). Specifically, the ring-bound N– and O– atoms introduce an antibonding node in the LUMO, while the remaining coumarins have a bonding node between the substituted ring-bound atom and neighboring carbon atoms. This effect can be quantified *via* signed R-group Mulliken populations in the LUMOs (Fig. 1D and Table S4†). Rationalizing this observation, HN– and O– substitutions add two electrons to the π-system, destabilizing the LUMO, while other R groups acquire π-electron density rendering the LUMOs more diffuse and therefore stabilizing. Of note, computed dipole moment, a standard measure of the electron-withdrawing capacity, does not correlate with *λ*^abs^_max_ (Table S4†).

### FC synthesis

**Scheme 1:**
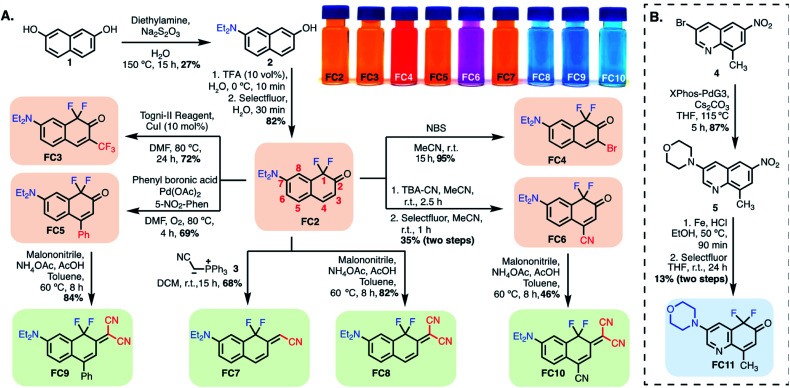
(A) Synthesis of FC dyes, **FC2–FC10**. (B) Quinoline-based fluoro-coumarin **FC11**. Color code: tangerine: FC variants modified at position-3/-4; green: position-2 modified FCs; blue: other FCs.

**Fig. 2:**
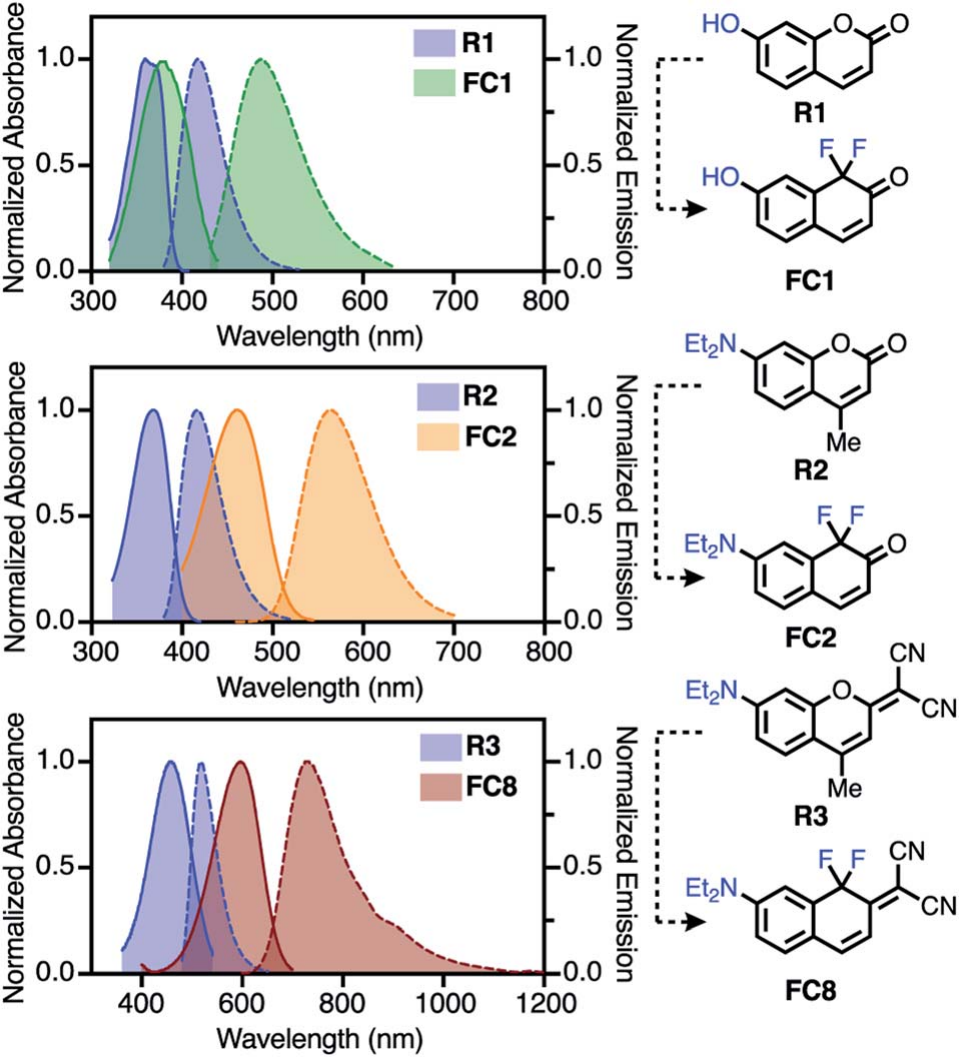
Comparison of **FC1**, **FC2** and **FC8** to reference coumarins **R1–R3** (in toluene), (solid = absorbance; dashed = emission).

Based on these computational results, we synthesized the significant series of FC dyes shown in Scheme 1A. The unsubstituted hydroxy derivative, **FC1** (Fig. 2), reported previously as a pharmaceutical precursor in a single study, was resynthesized.^34^ To generate the diethylamino derivatives, the first step involved a scalable Brucherer reaction of naphthalene **1** with diethylamine at 150 °C to provide **2** in modest, albeit reproducible, yield (27%). After significant screening, the combination of acidic conditions (TFA, H_2_O) and Selectfluor was found to regioselectively fluorinate **2** providing **FC2** in high yield (82%) on gram-scale. By contrast, optimal basic conditions (LiHMDS) were lower yielding (38%). Noting prior efforts on the coumarin scaffold, we sought to modify the −3 and −4 positions by derivatization of **FC2**.^28,35–37^ CF_3_ was introduced at the −3 position *via* copper-catalyzed electrophilic trifluoromethylation using the Togni-II reagent to provide **FC3**.^38^ 3-bromo-substituted **FC4** was accessed by treating **FC2** with NBS under aprotic conditions. A palladium-catalyzed oxidative Heck coupling was used to introduce a phenyl substituent at the −4 position to provide **FC5**.^39^ The 4-nitrile substituted derivative was synthesized by reacting **FC2** with *tert*-butylammonium cyanide, providing the mono-fluoro intermediate, which was then regioselectively fluorinated to give **FC6**.

Next, we sought to modify the −2 position to build donor–acceptor merocyanine-type derivatives, which find use as solvatochromic and stimuli-responsive probes (Scheme 1A).^22,23^ The mono-nitrile **FC7** was synthesized *via* Wittig reaction with the phosphine-ylide salt **3**. The difluoro ketones **FC2**, **FC5**, and **FC6** underwent Knovenagel condensation with malononitrile in the presence of ammonium acetate and acetic acid in toluene at 60 °C to provide **FC8**, **FC9** and **FC10**, respectively. These reactions highlight the ketone reactivity of the FC dyes and do not occur on the lactone of conventional coumarins.^20,22^ To tune the physical properties and sub-cellular localization of these dyes, we also prepared quinoline-derivative **FC11** (Scheme 1B). In brief, previously known **4** underwent palladium-catalyzed coupling with morpholine to provide **5**, which was subjected to Fe-reduction and electrophilic fluorination to access **FC11**.^40,41^ Finally, we initially investigated the synthesis of cyanine-type derivatives related to those recently reported by Sletten and coworkers, but found polymethine linked dimers to be unstable with FC monomers.^42^

### Photophysical characterization

We first characterized molecules where introduction of the difluoro-substituent represents a single-atom chromophore substitution from reference molecules **R1–R3**. Relative to 7-hydroxycoumarin **R1**, **FC1** exhibits significant bathochromic shifts in *λ*^abs^_max_ (*Δ* = 86 nm) and *λ*^em^_max_ (*Δ* = 135 nm) in 10% Fetal Bovine Serum (FBS), with less dramatic shifts in toluene (*Δ* = 18 and 72 nm, respectively). Compared to **R2**, **FC2** exhibits a large red-shift in the *λ*^abs^_max_ (*Δ* = 109 nm) and *λ*^em^_max_ (*Δ* = 130 nm) spectra in FBS, with a similar red-shift in *λ*^em^_max_ (*Δ* = 146 nm) in toluene. Finally, the donor–acceptor dye **FC8** exhibits the most dramatic bathochromic shift (*Δ λ*^abs^_max_/*λ*^em^_max_ = 130/217 nm (toluene) and 140/215 nm (FBS)) compared to **R3**.^22^ Also notable with **FC8** is the large Stokes shift of 144 (3265 cm^−1^) and 110 (2395 cm^−1^) nm in toluene and FBS, respectively. In general, all the FC dyes (except **FC11**) exhibited low solubility and weak emission in neutral aqueous media. By contrast, significant emission in FBS indicative of a possible serum binding of these hydrophobic dyes was recorded.

Additional striking observations are apparent upon inspecting the optical properties of the FC dyes (Table 1, Section S3). Introducing electron withdrawing groups at the −4 position had a dramatic effect on the *λ*^abs^_max_, with a 69 nm red-shift between **FC2** (R = H) and **FC6** (R = CN), which correlates with Hammett values across the series (Fig. S2†). Compound **FC10**, which combines the effect of the 4-nitrile and 2-malononitrile substitution, exhibits exceptionally red-shifted spectra with *λ*^abs^_max_/*λ*^em^_max_ of 694/807 nm in FBS and 668/770 nm in toluene. Additionally, and indicative of the positive solvatochromism of these dyes, the *λ*^em^_max_ of **FC10** is 902 nm in MeCN (Fig. S12†). A final feature of **FC8–FC10** is significant emission beyond 1000 nm (Fig. 2, S10, and S12†). This is noteworthy as off-peak >1000 nm emission has been harnessed with other cyanine-class dyes for high-resolution *in vivo* imaging.^43^ To the best of our knowledge, compounds **FC8–FC10** are the lowest molecular weight fluorophores known with *λ*^abs^_max_ over 600 nm and *λ*^em^_max_ over 700, and 800, respectively (Table S2†). As observed with other strategies that lead to bathochromic shifts, the longest-wavelength emitting compounds exhibit moderately reduced *Φ*_F_.^44^ Additionally, red-shifted FC dyes were generally found to have moderately lower molar extinction coefficients compared to the corresponding reference dyes. Similar trends were observed with previously reported modifications to the C-1 oxygen atom.^29,30^ The quinoline-based **FC11** is only modestly red-shifted (*Δ* = 37 nm) in its *λ*^abs^_max_ but has a 132 nm shift in *λ*^em^_max_, compared to **R2**. We have also initially probed the photostability these dyes (see Section S4). Of particular note, 2-malononitrile substituted dyes (**FC8–FC10**) exhibit exceptional photostability (Fig. S14†) in organic solvents.

**Table 1:** Summary of the photophysical properties of **FC1–FC11** and **R1–R3** in toluene and 10% FBS

Entry	*λ* ^abs^_max_ (nm)	*λ* ^em^_max_ (nm)	*ε* × 10^3^ (M^−1^ cm^−1^)	*Φ*_F_ b
	[Tol/FBS]^a^	[Tol/FBS]^a^	[Tol/FBS]^a^	[Tol]
**FC1**	380/467	489/578	8.0/7.2	0.54
**FC2**	461/491	565/575	12/11	0.74
**FC3**	483/500	567/572	14/13	0.81
**FC4**	493/514	597/598	13/12	0.59
**FC5**	470/480	570/572	8.7/7.8	0.75
**FC6**	530/560	636/630	12/5.6	0.55
**FC7**	469/480	580/565	13/15	0.46
**FC8**	596/625	740/735	9.2/8.9	0.32
**FC9**	602/608	737/725	8.9/7.7	0.23
**FC10**	668/694	770/807	11/6.6	0.09
**FC11**	408/422	551/531	14/7.9	0.57
**R1**	362/381	417/443	25/20	0.81 (ref. 45)
**R2**	371/382	419/445	25/23	0.88
**R3**	466/485	523/520	32/30	0.03

a Optical properties were measured in toluene (Tol) and 10% FBS in pH 7.4 PBS.

b Absolute *Φ*_F_ were measured using an integrating sphere.^32^ See Table S1 and Section S3 for additional data and discussion.

### Cellular imaging

**Fig. 3:**
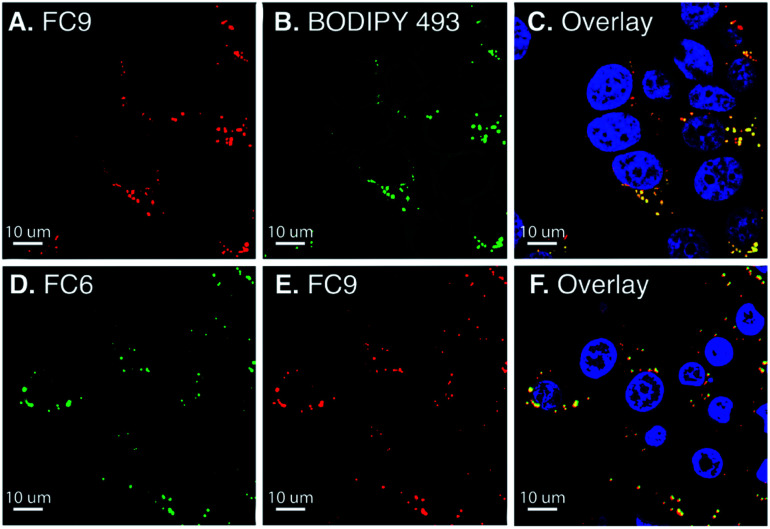
Laser scanning confocal microscopy of live MCF-7 cells incubated for 30 min (at 37 °C) in phenol red free DMEM with **FC9** (A–C) and **FC6** (D–F) (15 μM). The co-stains used were BODIPY 493/503 (lipid droplets) and Hoechst (nucleus). Red channel (A and E): excitation at 594 nm, emission collected: 665–759 nm. Green channel (B and D): excitation at 488 nm, emission collected: 489–553 nm. Blue channel (C and F): excitation at 405 nm, emission collected: 410–481 nm. The overlaid image corresponds to the combined red, green and blue channels. Pearson's coefficient, which denotes the goodness of colocalization, is calculated to be 0.81 (**FC6**) and 0.89 (**FC9**). Scale bar is 10 μm.

Solvent sensitive dyes have been broadly used for probing the dynamics of protein activity and monitoring the biophysical properties of lipid bilayers.^46–48^ In addition to positive solvatochromism (see Fig. S5 and S6†), **FC2** and **FC8** demonstrate a 30- and 40-fold increase in the emission intensity (MeOH → toluene, Fig. S13†). The solvent-sensitive nature of these dyes led us to examine their ability to passively stain lipid droplets (LD).^48^ Current red-emissive lipid droplet stains typically require fixation before imaging (Lipid-Tox) or viscosity modulators (NLV-1).^49,50^ Live-cell confocal imaging was performed using BODIPY 493/503 as a reference LD marker (Fig. 3). **FC8–FC10** dyes (15 μM) were incubated with MCF-7 cells for 30 min and a colocalization between **FC9** and BODIPY 493/503, with a Pearson's coefficient of 0.82, was noted. Next, **FC2–FC6** were tested, and due to their extensive cross talk with other commercial LD markers (Nile red and BODIPY 493/503), **FC9** was used as a uniquely long-wavelength reference standard. Of these, **FC6** was found to passively stain LDs with a Pearson's coefficient of 0.81. Also, notable, the more polar quinoline derivative **FC11** exhibits high nuclear localization (Pearson's coefficient: 0.88; Fig. S15†), demonstrating the versatility of FC dyes.

**Fig. 4:**
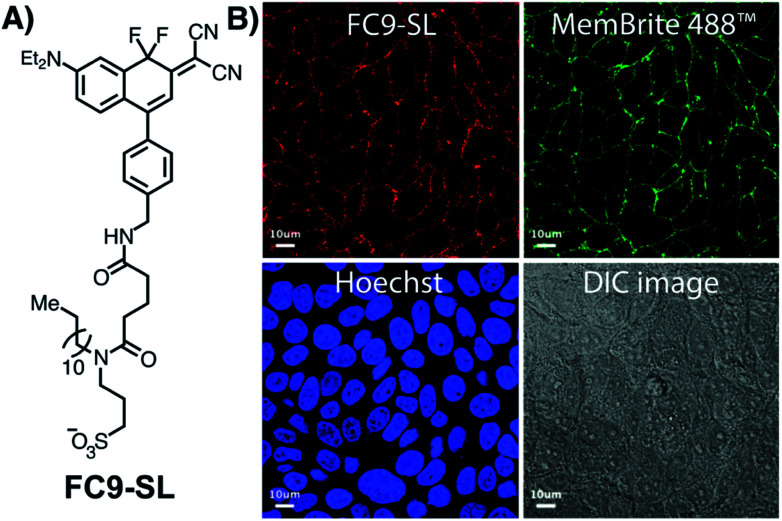
(A) Structure of **FC9-SL**. (B) Laser scanning confocal microscopy of live MCF-7 cells incubated for 30 min (at 37 °C) in phenol red free DMEM in the presence of **FC9-SL** (15 μM) and the co-stains MemBrite 488 and Hoechst dyes. Blue channel: excitation at 405 nm, emission collected: 410–481 nm. Red channel: excitation at 594 nm, emission collected: 650–759 nm. Scale bar is 10 μm.

The lipid droplet labeling of untargeted FC dyes prompted us to generate a plasma-membrane targeted variant. Existing plasma membrane dyes are often difficult to apply in live-cell and tissue settings due to their poor physical properties.^51^ Noting recent studies by Klymchenko,^52^ we chose to use an anionic sulfonate/lipophilic alkyl chain targeting group. The sulfo-lipid derivative **FC9-SL** was constructed over six steps from **FC2** (Scheme S1†). Live-cell imaging studies, conducted in MCF-7 cells by co-incubating **FC9-SL** with MemBrite 488 in HBSS for 30 min, demonstrated clear plasma membrane targeting (Fig. 4). These results demonstrate that FC dyes can be actively targeted to discrete lipid-rich environments.

## Conclusions

In summary, we report the impact of installing an electron-withdrawing difluoro-methylene functional group on the coumarin core. Substituted FC dyes exhibit large Stokes shifts and *λ*^abs^_max_ and *λ*^em^_max_ that span the far-red to NIR range. These dyes display excellent brightness in hydrophobic solvents, with untargeted variants exhibiting high specificity for lipid droplets and the nucleus. Active targeting through functionalization with a sulfo-lipid derivative provides plasma membrane-specific labeling with emission in the NIR range. Given that fluorination is often employed to improve small molecule pharmacokinetics, these FC dyes have excellent potential for *in vivo* applications.^53^ Furthermore, as electrophilic fluorination is an efficient strategy for the preparation of radiolabelled reagents,^54,55^ the direct incorporation of the ^18^F isotope creates the possibility of using this scaffold as a multi-modal contrast agent. We also envision that the effect of difluoro-substitution can be translated to other scaffolds leading to similarly dramatic bathochromic shifts. Currently, the development of related dyes and long wavelength photocleavable protecting groups is underway.

## Conflicts of interest

There are no conflicts to declare.

## Supplementary Material

SC-011-D0SC02566F-s001
