# Supplementary material for: Core remodeling leads to long wavelength fluoro-coumarins
Source: Chem Sci. 2020 Jul 3;11(28):7302–7. doi: 10.1039/d0sc02566f (PMC8159424; doi:10.1039/d0sc02566f)
Supplement: SC-011-D0SC02566F-s001 [file SC-011-D0SC02566F-s001.pdf]

Electronic Supplementary Material (ESI) for Chemical Science.
This journal is © The Royal Society of Chemistry 2020

Core Remodeling Leads to Long Wavelength Fluoro-Coumarins

Siddharth S. Matikonda, Joseph Ivanic, Miguel Gomez, Gabrielle Hammersley, Martin J. Schnermann

Table of Contents:

Section S1: General Materials and Methods 2

Section S2: Synthesis 3

Section S3: Optical Characterization of FC dyes and R1–R3 17

S3.1 Hammett Analysis 18

S3.2 Discussion of optical properties of FC dyes 20

Section S4: Photostability studies 31

Section S5: Cell Culture 32

Section S6: Confocal Cellular Imaging 32

S6.1 General procedure for Passive organelle staining experiments 32

S6.2 Confocal imaging experiments 32

S6.3 General procedure for Membrane Staining Experiments 34

Section S7: Computational Analysis 35

Section S8: NMR Spectra 41

Section S9: Raw Data from Computational Analysis 68

Section S10: References 84

Section S1: General Materials and Methods

Unless stated otherwise, reactions were conducted in oven- dried glassware under an atmosphere of nitrogen or argon using anhydrous solvents (passed through activated alumina columns). All commercially obtained reagents were used as received. Dulbecco's modified Eagle's Medium (DMEM), fetal bovine serum (FBS), Hank's balanced salt solution (HBSS), phenol red-free DMEM, and phosphate-buffered saline (PBS) was purchased from Quality Biologicals. Organelle stains, To-Pro™-3 iodide (642/661) and BODIPY 493/503 were purchased from Thermo Fisher and used as advised. MemBrite Fix 488/515 was purchased from Biotium and used according to the protocol provided. Flash column chromatography was performed using reversed phase (100 Å, 20-40-micron particle size, RediSep® Rf Gold® Reversed-phase C18 or C18Aq) and silica on a CombiFlash® Rf 200i (Teledyne Isco, Inc.). High-resolution LC/MS analyses were conducted on a Thermo-Fisher LTQ-Orbitrap-XL hybrid mass spectrometer system with an Ion MAX API electrospray ion source in negative ion mode. Analytical LC/MS was performed using a Shimadzu LC/MS-2020 Single Quadrupole utilizing a Kinetex 2.6 µm C18 100 Å (2.1 × 50 mm) column obtained from Phenomenex, Inc. Runs employed a gradient of 0→90% MeCN/0.1% aqueous formic acid over 4.5 min at a flow rate of 0.2 mL/min. ^{1}H NMR and ^{13}C NMR spectra were recorded on Bruker spectrometers (at 400 or 500 MHz or at 100 or 125 MHz, respectively) and are reported relative to deuterated solvent signals (and $CDFCl_2$ for ^{19}F NMR). Data for ^{1}H NMR spectra are reported as follows: chemical shift (δ ppm), multiplicity, coupling constant (Hz), and integration. Data for ^{13}C, ^{1}H and ^{19}F NMR spectra are reported in terms of chemical shift. Absorption curves for quantum yield measurements were performed on a Shimadzu UV-2550 spectrophotometer operated by UVProbe 2.32 software. Fluorescence traces were recorded on a PTI QuantaMaster steady state spectrofluorometer operated by FelixGX 4.2.2 software, with 5 nm excitation and emission slit widths, 0.1 s integration rate, and enabled emission correction. Data analysis and curve fitting were performed using MS Excel 2011 and GraphPad Prism 7. Light intensity measurements were performed with a Thorlabs PM200 optical power and energy meter fitted with an S120VC standard Si photodiode power sensor (200-1100 nm, 50 mW). Cellular imaging was performed using a Zeiss LSM 810 confocal laser-scanning microscope. Cells were plated on CELLview™ slides, advanced TC™ Surface and sterile (VWR). Image processing was conducted using Icy software.

Section S2: Synthesis

Synthesis of 1,1-difluoro-7-hydroxynaphthalen-2(1H)-one (FC1)[1]:

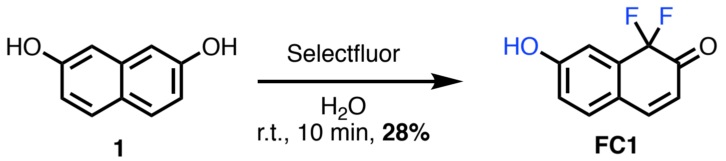

This compound was prepared through a modified version of the original procedure, which provides more reproducible yields. A solution of naphthalene-2,7-diol (2.0 g, 12.4 mmol) and Selectfluor (8.84 g, 24.9 mmol) dissolved in water (12.5 mL) was allowed to stir at room temperature. The progress of the reaction was monitored using thin-layer chromatography (TLC) and upon completion (10 min), the reaction mixture was quenched using sat. NH_4Cl (20 mL), neutralized with sat. $NaHCO_3$ (50 mL) and extracted using DCM (~400 mL). The organic fraction was washed with H_2O (2 × 100 mL) and brine (50 mL) then dried (Na_2SO_4) and concentrated *in vacuo*. The crude mixture was purified using normal-phase column chromatography (24 g silica; 0→20% EtOAc: Hexane) to result in **FC1** (687 mg, 28%) as an amorphous yellow powder, which was spectroscopically similar to that previously reported.[1] ^{1}H NMR (400 MHz, MeOD) δ 7.56 (d, *J* = 10.0 Hz, 1H), 7.35 (d, *J* = 8.4 Hz, 1H), 7.20 (m, 1H), 6.95 (m, 1H), 6.01 (dt, *J* = 10.0, 2.8 Hz, 1H).

Synthesis of 7-(diethylamino)naphthalen-2-ol (2):

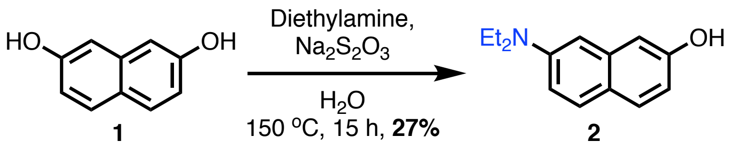

In a pressure tube, a solution of naphthalene-2,7-diol **1** (10.0 g, 62.48 mmol) and sodium metabisulfite (23.7 g, 0.124 mol) in water (32 mL) was prepared. To this solution, diethylamine (22.8 g, 0.31 mol) was added and the tube was sealed. The contents in the pressure tube were heated to 150 °C for 15 h. After this time, the cooled reaction mixture was transferred to a separating funnel and basified to a pH = 12 using 1M NaOH. The organics were extracted using EtOAc (300 mL), washed with brine (100 mL), dried (Na_2SO_4) and concentrated *in vacuo.* The crude mixture was purified using normal-phase column chromatography (330 g silica; 0→25% EtOAc in Hexane) to result in **2** (3.7 g, 27%) as a brown viscous liquid. ^{1}H NMR (500 MHz, $CDCl_3$) δ 7.67 (d, *J* = 9.0 Hz, 2H), 7.62 (d, *J* = 9.0 Hz, 1H), 7.03 (dd, *J* = 9.0, 2.5 Hz, 1H), 6.99 (s, 1H), 6.91 (dd, *J* = 9.0, 2.0 Hz, 1H), 6.79 (d, *J* = 2.5 Hz, 1H), 6.54 (s, 1H) 3.46 (q, *J* = 7.0 Hz, 4H), 1.25 (t, *J* = 7.0 Hz, 6H). ^{13}C {^{1}H} NMR (125 MHz, $CDCl_3$) δ 154.0, 146.3, 136.6, 129.3, 128.8,

122.1, 114.1, 113.8, 108.1, 105.3, 44.6, 12.5. HRMS (ESI) m/z: $[M+H]^+$ Calcd. for $C_{14}H_{17}NO$ 216.1310; Found 216.1306.

Synthesis of 7-(diethylamino)-1,1-difluoronaphthalen-2(1H)-one (FC2):

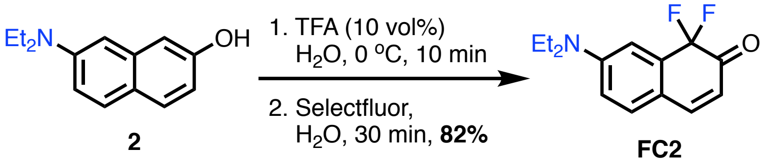

Acidic conditions: To a cooled suspension (ice bath) of 7-(diethylamino)naphthalen-2-ol **2** (1.7 g, 7.90 mmol) in H_2O (36 mL) was added TFA (4 mL, 10 vol%) and allowed to stir for 10 min. To this solution, Selectfluor (6.2 g, 17.4 mmol) dissolved in H_2O (15 mL) was added dropwise over 20 min. The reaction mixture was allowed to warm to 0 ºC and stirred for 30 min. The progress of the reaction was monitored using LC/MS and TLC (every aliquot collected for analysis was quenched with a sat. NH_4Cl and neutralized with sat. $NaHCO_3$). Upon completion (1 h), the reaction mixture was quenched using sat. NH_4Cl (50 mL) and transferred to a conical flask and cooled to 0 ºC in an ice bath. Sat. $NaHCO_3$ was slowly added to set the pH of the aqueous mixture between 7 – 8, after which the organics were extracted using EtOAc (2 × 150 mL). The organic fraction was washed with H_2O (2 × 150 mL) and brine (2 × 100 mL) then dried (Na_2SO_4) and concentrated *in vacuo*. The crude mixture was purified using normal-phase column chromatography (40 g silica; 0→15% EtOAc: Hexane) to result in **FC2** (1.63 g, 82%) as a thick viscous dark red liquid: ^{1}H NMR (400 MHz, $CDCl_3$) δ 7.42 (d, J = 10.0 Hz, 1H), 7.21 (d, J = 8.8 Hz, 1H), 7.10 – 7.03 (m, 1H), 6.71 (dd, J = 8.8, 2.4 Hz, 1H), 5.83 (dt, J = 9.6, 3.2 Hz, 1H), 3.43 (q, J = 7.2 Hz, 4H), 1.14 (t, J = 6.8 Hz, 6H). $^{13}C\{^1H\}$ NMR (100 MHz, CD_3CN) δ 188.3 (t, $J_{C\text{-}F}$ = 24 Hz), 150.8 (t, $J_{C\text{-}F}$ = 1 Hz), 148.6, 136.3 (t, $J_{C\text{-}F}$ = 22 Hz), 133.4, 117.8 (t, $J_{C\text{-}F}$ = 5 Hz), 117.4 (t, $J_{C\text{-}F}$ = 2 Hz), 113.8, 111.8 (t, $J_{C\text{-}F}$ = 4 Hz), 107.3 (t, $J_{C\text{-}F}$ = 243 Hz), 45.3, 12.7. ^{19}F NMR (376 MHz, CD_3CN) –99.25. HRMS (ESI) m/z: $[M+H]^+$ Calcd. for $C_{14}H_{16}F_2NO$ 252.1194; Found 252.1191.

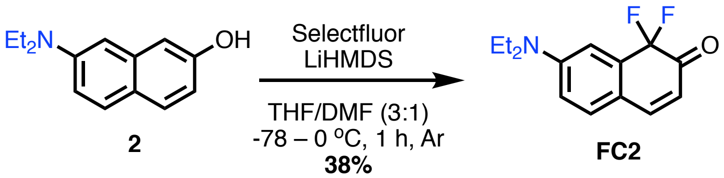

Optimal basic conditions: A solution of 7-(diethylamino)naphthalen-2-ol **2** (696 mg, 2.77 mmol) in THF was cooled to –78 ºC. To this solution, LiHMDS (3.81 mL of a 1M solution, 638 mg, 3.81 mmol) was added dropwise over 10 min at –78 ºC and allowed to stir for 30 min under argon. In

a separate flask, a solution of Selectfluor (1.38 g, 3.90 mmol) was dissolved in DMF and added to the first mixture dropwise over 20 min at –78 ºC under argon. The reaction mixture was allowed to warm to 0 ºC and stirred for 1 h. The progress of the reaction was monitored using LC/MS and TLC (every aliquot collected for analysis was quenched with a sat. NH_4Cl). Upon completion (1 h), the reaction mixture was quenched using sat. NH_4Cl (50 mL) and extracted using EtOAc (200 mL). The organic fraction was washed with H_2O (2 × 50 mL) and brine (2 × 30 mL) then dried (Na_2SO_4) and concentrated *in vacuo*. The crude mixture was purified using normal-phase column chromatography (120 g silica; 0→15% EtOAc: Hexane) to result in **FC2** (311 mg, 38%) as a thick viscous dark red liquid.

Synthesis of 7-(diethylamino)-1,1-difluoro-3-(trifluoromethyl)naphthalen-2(1H)-one (FC3):

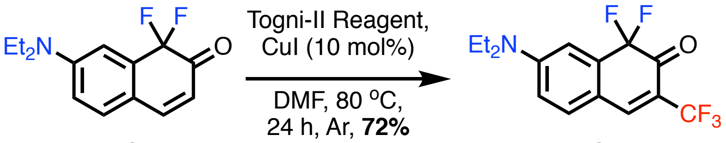

A solution of 7-(diethylamino)-1,1-difluoronaphthalen-2(1H)-one **FC2** (100 mg, 0.40 mmol), 1-Trifluoromethyl-1,2-benziodoxol-3(1H)-one [Togni-II reagent][1] (378 mg, 1.19 mmol) and copper iodide (7.6 mg, 10 mol%) in DMF (1 mL, 0.4 M), was heated to 80 ºC for 24 h under argon. The progress of the reaction was monitored using LC/MS and TLC. Upon completion (24 h), the reaction was quenched by the addition of water (5 mL) and the organics were extracted using EtOAc (100 mL). The organic fraction was washed with H_2O (2 × 100 mL) and brine (100 mL) then dried (Na_2SO_4) and concentrated *in vacuo*. The crude mixture was purified using normal-phase column chromatography (24 g silica; 0→10% EtOAc: Hexane) to result in **FC3** (92 mg, 72%) as a thick viscous dark red liquid: ^{1}H NMR (400 MHz, CD_3CN) δ 7.95 (s, 1H), 7.43 (d, J = 8.8 Hz, 1H), 7.19 – 7.14 (m, 1H), 6.82 (dd, J = 8.8, 2.8 Hz, 1H), 3.51 (q, J = 6.8 Hz, 4H), 1.19 (t, J = 6.8 Hz, 6H). ^{13}C {^{1}H} NMR (125 MHz, CD_3CN) δ 183.8 (t, $J_{C\text{-}F}$ = 26 Hz), 152.9 (t, $J_{C\text{-}F}$ = 1 Hz), 148.9 (q, $J_{C\text{-}F}$ = 10, 5 Hz), 137.7 (t, $J_{C\text{-}F}$ = 22 Hz), 136.5, 123.9 (q, $J_{C\text{-}F}$ = 269, 2 Hz), 116.1 (qt, $J_{C\text{-}F}$ = 61, 31, 3 Hz), 114.6 (t, $J_{C\text{-}F}$ = 5 Hz), 114.1, 112.8 (t, $J_{C\text{-}F}$ = 4 Hz), 107.4 (t, $J_{C\text{-}F}$ = 243 Hz), 46.0, 13.0. ^{19}F NMR (376 MHz, CD_3CN) –63.13, –98.68. HRMS (ESI) m/z: $[M+H]^+$ Calcd. for $C_{15}H_{15}F_5NO$ 320.1062; Found 320.1062.

Synthesis of 3-bromo-7-(diethylamino)-1,1-difluoronaphthalen-2(1H)-one (FC4):

[1] The reaction was higher yielding when Togni-II reagent was used compared to Togni-I reagent.

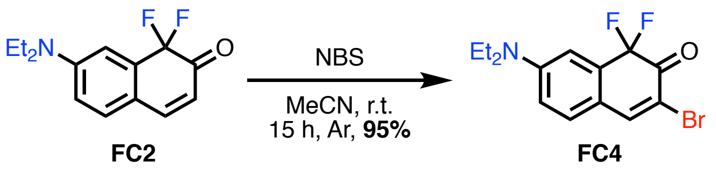

A solution of 7-(diethylamino)-1,1-difluoronaphthalen-2(1H)-one **FC2** (10 mg, 0.04 mmol) and N-bromosuccinamide (8.5 mg, 0.05 mmol) in MeCN (300 µL), was stirred at r.t. for 15 h under argon. The progress of the reaction was monitored using LC/MS and TLC. Upon completion (15 h), the reaction was quenched by the addition of water (5 mL) and the organics were extracted using EtOAc (30 mL). The organic fraction was washed with H_2O (2 × 10 mL) and brine (10 mL) then dried (Na_2SO_4) and concentrated *in vacuo*. The crude mixture was purified using normal-phase column chromatography (4 g silica; 0→7.5% EtOAc: Hexane) to result in **FC4** (13 mg, 95%) as a thick viscous maroon liquid: ^{1}H NMR (500 MHz, $CDCl_3$) δ 7.70 (s, 1H), 7.12 (d, J = 8.5 Hz, 1H), 7.07 – 7.04 (m, 1H), 6.62 (dd, J = 9.0, 2.5 Hz, 1H), 3.45 (q, J = 7.5 Hz, 4H), 1.23 (t, J = 7.0 Hz, 6H). ^{13}C {^{1}H} NMR (125 MHz, $CDCl_3$) δ 181.8 (t, $J_{C\text{-}F}$ = 25 Hz), 150.0, 148.6, 135.4 (t, $J_{C\text{-}F}$ = 23 Hz), 132.1, 116.8 (t, $J_{C\text{-}F}$ = 5 Hz), 112.6 (t, $J_{C\text{-}F}$ = 5 Hz), 111.8 (t, $J_{C\text{-}F}$ = 4 Hz), 110.7 (t, $J_{C\text{-}F}$ = 3 Hz), 106.7 (t, $J_{C\text{-}F}$ = 246 Hz), 44.8, 12.5. ^{19}F NMR (376 MHz, $CDCl_3$) –97.53. HRMS (ESI) m/z: $[M+H]^+$ Calcd. for $C_{14}H_{15}{}^{81}BrF_2NO$ 332.0380; Found 332.0371.

Synthesis of 7-(diethylamino)-1,1-difluoro-4-phenylnaphthalen-2(1H)-one (FC5):

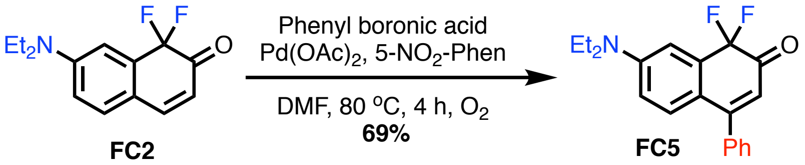

7-(diethylamino)-1,1-difluoronaphthalen-2(1H)-one (**FC2**) (95 mg, 0.38 mmol), phenyl boronic acid (52 mg, 0.43 mmol), palladium acetate (81 mg, 0.36 mmol) and 5-NO_2-1,10-phenanthroline (81 mg, 0.36 mmol) were sealed in a microwave vial.[2] This reaction vessel was purged with O_2, following which DMF (4.5 mL) was added. The solvent was further purged with O_2 for 10 minutes and then the reaction mixture was heated to 80 °C. The progress of the reaction was monitored using LC/MS and TLC. Upon completion (4 h), water (5 mL) was added to the reaction mixture and the organics were extracted using EtOAc (100 mL). The organic fraction was washed with H_2O (2 × 50 mL) and brine (50 mL) then dried (Na_2SO_4) and concentrated *in vacuo*. The crude mixture was purified using normal-phase column chromatography (24 g silica; 0→7.5% EtOAc: Hexane) to result in **FC5** (86 mg, 69%) as a thick viscous dark red liquid: ^{1}H NMR (400 MHz,

$CDCl_3$) δ 7.52 – 7.35 (m, 5H), 7.17 (dt, J = 3.2, 1.6 Hz, 1H), 7.09 (dt, J = 8.8, 1.2 Hz, 1H), 6.56 (dd, J = 9.2, 3.2 Hz, 1H), 5.91 (t, J = 2.8 Hz, 1H), 3.45 (q, J = 7.2 Hz, 4H), 1.22 (t, J = 6.8 Hz, 6H). ^{13}C {^{1}H} NMR (125 MHz, $CDCl_3$) δ 187.4 (t, J_{C-F} = 24 Hz), 159.2, 149.5, 137.8, 136.2 (t, J_{C-F} = 23 Hz), 131.8, 129.4, 128.7, 128. 3, 117.7 (t, J_{C-F} = 2 Hz), 117.4 (t, J_{C-F} = 4 Hz), 112.0, 111.5 (t, J_{C-F} = 4 Hz), 106.6 (t, J_{C-F} = 243 Hz), 44.8, 12.7. ^{19}F NMR (376 MHz, $CDCl_3$) –100.48 (d, J_{C-F} = 4 Hz). HRMS (ESI) m/z: $[M+H]^+$ Calcd. for $C_{220}F_2NO$ 328.1507; Found 328.1503.

6-(diethylamino)-4,4-difluoro-3-oxo-3,4-dihydronaphthalene-1-carbonitrile (FC6):

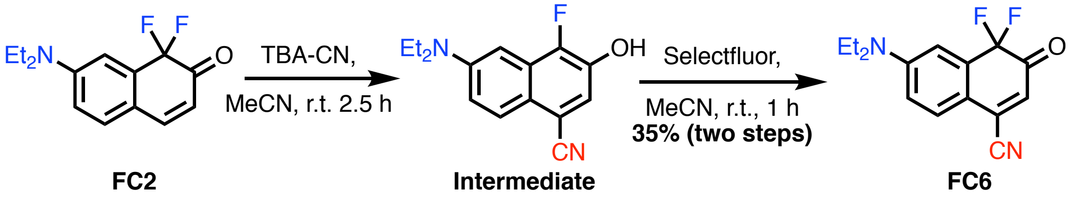

Step 1: 7-(diethylamino)-1,1-difluoronaphthalen-2(1H)-one (**FC2**) (165 mg, 0.66 mmol) was reacted with tert-butylammonium cyanide (353 mg, 1.31 mmol) in MeCN (3 mL) for 2.5 h. The progress of the reaction was monitored by LC/MS and after the starting material was consumed resulting in the formation of a peak at m/e = 259.1, the reaction was quenched by the addition of water (5 mL). The organics were extracted into EtOAc (100 mL) and washed with water (2 × 50 mL), brine (50 mL), dried (Na_2SO_4) and concentrated *in vacuo*. The crude concentrate was purified using normal-phase column chromatography (24 g silica; 0→10% EtOAc: Hexane) to result in 115 mg (yield = 68%) of the intermediate which was subjected to fluorination.

Step 2: To a solution of the previous intermediate (115 mg, 0.45 mmol) in MeCN (5 mL), Selectfluor (158 mg, 0.45 mmol) was added in small batches. The reaction was allowed to stir at r.t. for 1 h and the progress of the reaction was monitored using LC/MS. Upon completion (1 h), the reaction was quenched by the addition of sat. NH_4Cl (20 mL) and the organics were extracted using EtOAc (50 mL). The organic fraction was washed with H_2O (2 × 25 mL) and brine (25 mL) then dried (Na_2SO_4) and concentrated *in vacuo*. The crude mixture was purified using normal-phase column chromatography (24 g silica; 0→5 % EtOAc: Hexane) to result in **FC6** (63 mg, 35% [two steps]) as a thick viscous purple liquid.^{1}H NMR (400 MHz, CD_3CN) δ 7.53 (d, J = 8.8 Hz, 1H), 7.21 – 7.16 (m, 1H), 6.85 (dd, J = 8.8, 2.8 Hz, 1H), 6.36 (t, J = 2.8 Hz, 1H), 3.51 (q, J = 7.2 Hz, 4H), 1.19 (t, J = 7.2 Hz, 6H). ^{13}C {^{1}H} NMR (125 MHz, $CDCl_3$) δ 186.2 (t, J_{C-F} = 25 Hz), 150.7, 135.4 (t, J_{C-F} = 23 Hz), 130.9, 129.1, 122.3 (t, J_{C-F} = 3 Hz), 115.2, 113.0 (t, J_{C-F} = 5 Hz),

112.6, 112.3 (t, $J_{C\text{-}F}$ = 4 Hz), 105.7 (t, $J_{C\text{-}F}$ = 245 Hz), 45.2, 12.6. ^{19}F NMR (376 MHz, $CDCl_3$) –98.31. HRMS (ESI) m/z: $[M+H]^+$ Calcd. for $C_{15}H_{17}F_2N_2O_2$ 277.1147; Found 277.1144.

(E)-2-(7-(diethylamino)-1,1-difluoronaphthalen-2(1H)-ylidene)acetonitrile (FC7):

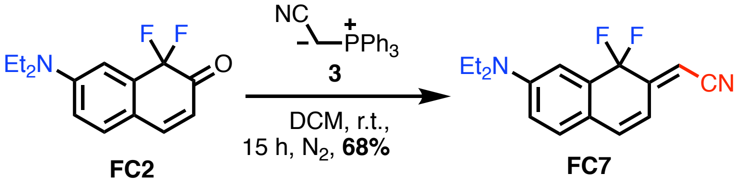

7-(diethylamino)-1,1-difluoronaphthalen-2(1H)-one (**FC2**) (50 mg, 0.19 mmol) and (Triphenylphosphoranylidene)acetonitrile **3** (90 mg, 0.30 mmol) were added to a 1-dram vial and purged with N_2. This mixture was dissolved in DCM (500 µL) under N_2 and allowed to stir at r.t. for 15 h. The progress of the reaction was monitored by LC/MS and after the starting material was consumed (15 h), the reaction mixture was concentrated to as minimum MeCN as possible. The organics were extracted into EtOAc (10 mL) and washed with water (2 × 10 mL), brine (10 mL), dried (Na_2SO_4) and concentrated *in vacuo*. The crude concentrate was purified using normal-phase column chromatography (4 g silica; 0→10% EtOAc: Hexane) to result in **FC7** (37 mg, 68%) as bright orange solid: ^{1}H NMR (400 MHz, $CDCl_3$) δ 7.12 (dt, J = 8.8, 1.6 Hz, 1H), 7.05 (m, 1H), 6.71 (d, J = 9.6 Hz, 1H), 6.67 (dd, J = 8.8, 2.8 Hz, 1H), 6.52 (dtd, J = 9.2f, 2.4, 0.8 Hz, 1H), 5.89 – 5.85 (m, 1H), 3.43 (q, J = 7.2 Hz, 4H), 1.21 (t, J = 7.2 Hz, 6H). ^{13}C {^{1}H} NMR (100 MHz, $CDCl_3$) δ 151.2 (t, $J_{C\text{-}F}$ = 21 Hz), 148.6, 133.6, 132.2 (t, $J_{C\text{-}F}$ = 23 Hz), 130.4, 119.0 (t, $J_{C\text{-}F}$ = 7 Hz), 116.0 (t, $J_{C\text{-}F}$ = 4 Hz), 115.4 (t, $J_{C\text{-}F}$ = 4 Hz), 113.0, 112.4 (d, $J_{C\text{-}F}$ = 238 Hz), 108.9 (t, $J_{C\text{-}F}$ = 4 Hz), 96.7 (t, $J_{C\text{-}F}$ = 21 Hz), 44.6, 12.5. ^{19}F NMR (376 MHz, $CDCl_3$) –78.92. HRMS (ESI) m/z: $[M+H]^+$ Calcd. for $C_{16}H_{17}F_2N_2$ 275.1354; Found 275.1349.

2-(7-(diethylamino)-1,1-difluoronaphthalen-2(1H)-ylidene)malononitrile (FC8):

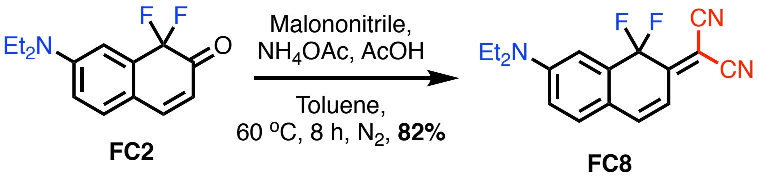

7-(diethylamino)-1,1-difluoronaphthalen-2(1H)-one (**FC2**) (50 mg, 0.19 mmol) was reacted with malononitrile (53 mg, 0.80 mmol), ammonium acetate (15 mg, 0.20 mmol) and glacial acetic acid (210 µL) in toluene (2.1 mL) under N_2 at 60 °C for 8 h. The progress of the reaction was monitored by LC/MS and after the starting material was consumed (8 h), water (5 mL) was added to the reaction mixture. The organics were extracted into EtOAc (50 mL) and washed with water (2 × 25

mL) and brine (25 mL), dried (Na_2SO_4) and concentrated *in vacuo*. The crude concentrate was purified using normal-phase column chromatography (24 g silica; 0→15% EtOAc: Hexane) to result in **FC8** (50 mg, 82%) as navy blue thick viscous liquid: ^{1}H NMR (400 MHz, $CDCl_3$) δ 7.18 (d, J = 8.8 Hz, 1H), 7.12 (s, 1H), 6.96 (d, J = 9.2 Hz, 1H), 6.66 (dd, J = 8.8, 2.8 Hz, 1H), 6.50 (dt, J = 9.2, 2.8 Hz, 1H), 3.48 (q, J = 7.2 Hz, 4H), 1.24 (t, J = 6.8 Hz, 6H). ^{13}C {^{1}H} NMR (100 MHz, $CDCl_3$) δ 158.6 (t, J_{C-F} = 21 Hz), 150.4, 141.2 (t, J_{C-F} = 2 Hz), 133.7 (t, J_{C-F} = 23 Hz), 132.6, 114.4 (t, J_{C-F} = 4 Hz), 112.9, 112.7 (t, J_{C-F} = 5 Hz), 112.5 (t, J_{C-F} = 3 Hz), 112.2 (t, J_{C-F} = 241 Hz), 117.7 (t, J_{C-F} = 4 Hz), 110.9 (t, J_{C-F} = 4 Hz), 80.3 (t, J_{C-F} = 5 Hz), 45.1, 12.7. ^{19}F NMR (376 MHz, $CDCl_3$) –79.03. HRMS (ESI) m/z: $[M+H]^+$ Calcd. for $C_{17}H_{16}F_2N_3$ 300.1307; Found 300.1300.

2-(7-(diethylamino)-1,1-difluoro-4-phenylnaphthalen-2(1H)-ylidene)malononitrile (FC9):

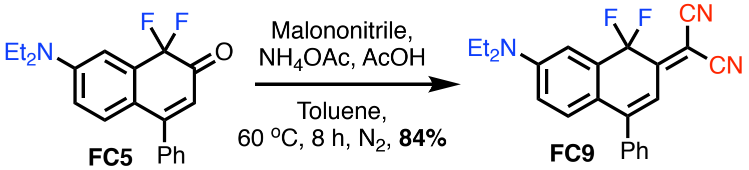

7-(diethylamino)-1,1-difluoro-4-phenylnaphthalen-2(1H)-one (**FC5**) (50 mg, 0.15 mmol) was reacted with malononitrile (72 mg, 0.61 mmol), ammonium acetate (12 mg, 0.15 mmol) and glacial acetic acid (210 µL) in toluene (2.1 mL) under N_2 at 60 ºC for 8 h. The progress of the reaction was monitored by LC/MS and after the starting material was consumed (8 h), water (5 mL) was added to the reaction mixture. The organics were extracted into EtOAc (100 mL) and washed with water (2 × 50 mL), brine (50 mL), dried (Na_2SO_4) and concentrated *in vacuo*. The crude concentrate was purified using normal-phase column chromatography (24 g silica; 0→15% EtOAc: Hexane) to result in **FC9** (48 mg, 84%) as navy blue thick viscous liquid: ^{1}H NMR (400 MHz, $CDCl_3$) δ 7.53 – 7.36 (m, 5H), 7.21 (m, 1H), 7.18 (m, 1H), 6.60 (dd, J = 9.2, 2.4 Hz, 1H), 6.50 (t, J = 2.8 Hz, 1H), 3.48 (q, J = 7.2 Hz, 4H), 1.24 (t, J = 7.2 Hz, 6H). ^{13}C {^{1}H} NMR (125 MHz, $CDCl_3$) δ 157.6 (t, J_{C-F} = 21 Hz), 153.9 (t, J_{C-F} = 3 Hz), 150.0, 137.5, 134.4 (t, J_{C-F} = 23 Hz), 132.1, 130.0, 128.9, 128.7, 117.3 (t, J_{C-F} = 5 Hz), 115.4 (t, J_{C-F} = 4 Hz), 113.1 (t, J_{C-F} = 4 Hz), 113.0 (t, J_{C-F} = 3 Hz), 112.5, 112.1 (t, J_{C-F} = 241 Hz), 111.1 (t, J_{C-F} = 4 Hz), 78.8 (t, J_{C-F} = 5 Hz), 45.1, 12.7. ^{19}F NMR (376 MHz, $CDCl_3$) –79.13 (d, J_{C-F} = 4 Hz). HRMS (ESI) m/z: $[M+H]^+$ Calcd. for $C_{23}H_{20}F_2N_3$ 376.1620; Found 376.1610.

2-(4-cyano-7-(diethylamino)-1,1-difluoronaphthalen-2(1H)-ylidene)malononitrile (FC10)[2]:

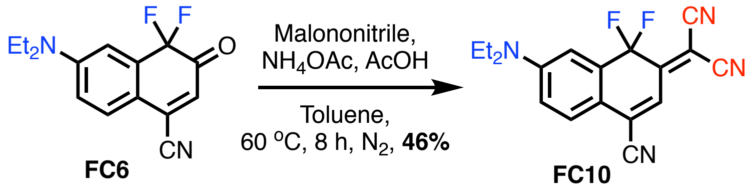

6-(diethylamino)-4,4-difluoro-3-oxo-3,4-dihydronaphthalene-1-carbonitrile (**FC6**) (12.5 mg, 0.045 mmol) was reacted with malononitrile (22 mg, 0.18 mmol), ammonium acetate (3.6 mg, 0.045 mmol) and glacial acetic acid (60 μL) in toluene (600 μL) under N_2 at 60 ºC for 8 h. The progress of the reaction was monitored by LC/MS and after the starting material was consumed (8 h), water (5 mL) was added to the reaction mixture. The organics were extracted into EtOAc (20 mL) and washed with water (2 × 10 mL), brine (10 mL), dried (Na_2SO_4) and concentrated *in vacuo*. The crude concentrate was purified using normal-phase column chromatography (4 g silica; 0→15% EtOAc: Hexane) to result in **FC10** (8 mg, 46%) as teal colored thick viscous liquid: ^1H NMR (500 MHz, $CDCl_3$) δ 7.55 (d, J = 9.0 Hz, 1H), 7.17 – 7.14 (m, 1H), 6.89 (t, J = 2.5 Hz, 1H), 6.75 (dd, J = 9.0, 3.0 Hz, 1H), 3.53 (q, J = 7.0 Hz, 4H), 1.27 (t, J = 7.0 Hz, 6H). ^{19}F NMR (376 MHz, $CDCl_3$) –76.66. HRMS (ESI) m/z: $[M+H]^+$ Calcd. for $C_{18}H_{15}F_2N_4$ 325.1259; Found 325.1254.

4-(8-methyl-6-nitroquinolin-3-yl)morpholine (5):

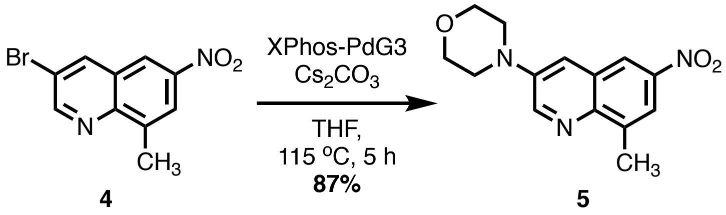

To a microwave vial containing 3-bromo-8-methyl-6-nitroquinoline **4** (2.2 g, 8.22 mmol), synthesized according to a previously reported literature procedure,[3] morpholine (2.1 g, 24.7 mmol) and Cs_2CO_3 (5.36 g, 16.4 mmol), was added XPhos-PdG3 (696 mg, 0.82 mmol) (in glove box) and sealed under N_2. To this mixture, THF (10 mL) was added under N_2 and heated at 115 ºC for 5 h. The progress of the reaction was monitored by TLC and after the starting material was consumed (5 h), the reaction mixture was filtered and washed with EtOAc (300 mL). The filtrate

[2] Characterization for this compound includes ^1H NMR, gCOSY and ^{19}F NMR and HRMS. Complex C-F splitting in the ^{13}C NMR precluded obtaining high quality spectra.

was then transferred to a separating funnel and washed with aq. sat. $NaHCO_3$ (2 × 200 mL), brine (100 mL), dried (Na_2SO_4) and concentrated *in vacuo*. The crude concentrate was purified using normal-phase column chromatography (120 g silica; 0→30% EtOAc: Hexane) to result in **5** (1.95 g, 87%) as yellow amorphous solid: ^{1}H NMR (500 MHz, CDCl3) δ 8.93 (d, J = 3.0 Hz, 1H), 8.45 (d, J = 3.0 Hz, 1H), 8.09 (dd, J = 2.5, 1.0 Hz, 1H), 7.40 (d, J = 3.0 Hz, 1H), 3.97 – 3.92 (m, 4H), 3.36 – 3.32 (m, 4H), 2.82 (s, 3H). ^{13}C {^{1}H} NMR (125 MHz, $CDCl_3$) δ 145.8, 145.7, 145.5, 143.7, 139.4, 127.9, 121.1, 119.6, 117.27, 66.5, 48.5, 18.2. HRMS (ESI) m/z: $[M+H]^+$ Calcd. for $C_{14}H_{16}N_3O_3$ 274.1186; Found 274.1181.

8-methyl-3-morpholinoquinolin-6-amine:

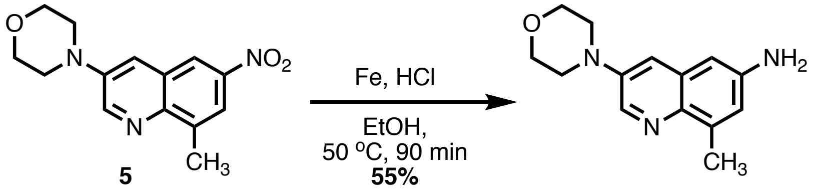

4-(8-methyl-6-nitroquinolin-3-yl)morpholine **5** (203 mg, 0.743 mmol), and iron powder (145 mg, 2.60 mmol) was dissolved in EtOH (6 mL) and 37% HCl (305 µL) was added dropwise. This mixture was heated to 50 ºC for 90 min. The progress of the reaction was monitored by TLC and after the starting material was consumed (90 min), the reaction mixture was quenched with $NaHCO_3$ to set the pH at 9. The crude was then diluted in EtOAc (100 mL) and washed with aq. sat. $NaHCO_3$ (2 × 100 mL), brine (50 mL), dried (Na_2SO_4) and concentrated *in vacuo*. The crude concentrate was purified using normal-phase column chromatography (40 g silica; 0→75% EtOAc: Hexane) to result in 100 mg (55%) of the title compound as beige colored solid: ^{1}H NMR (500 MHz, $CDCl_3$) δ 8.54 (d, J = 3.0 Hz, 1H), 7.09 (d, J = 3.0 Hz, 1H), 6.83 (m, 1H), 6.64 (d, J = 3.0 Hz, 1H), 3.95 – 3.89 (m, 4H), 3.83 (s, 2H), 3.27 – 3.21 (m, 4H), 2.68 (s, 3H). ^{13}C {^{1}H} NMR (125 MHz, $CDCl_3$) δ 144.9, 144.8, 139.4, 138.0, 137.6, 130.4, 119.1, 115.3, 104.9, 66.8, 49.4, 18.0. HRMS (ESI) m/z: $[M+H]^+$ Calcd. for $C_{14}H_{18}N_3O$ 244.1444; Found 244.1440

5,5-difluoro-8-methyl-3-morpholinoquinolin-6(5H)-one (FC11):

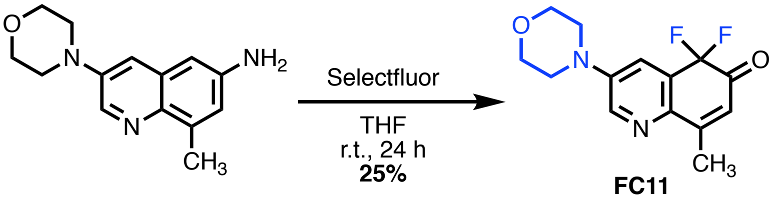

To a cooled (0 ºC, ice bath) solution of 8-methyl-3-morpholinoquinolin-6-amine (100 mg, 0.41 mmol) dissolved in THF (2 mL), Selectfluor (146 mg, 0.41 mmol) was added in small batches. The reaction mixture was allowed to warm to r.t. and left to stir for 24 h. The progress of the reaction was monitored by LC/MS and TLC (every aliquot collected for analysis was quenched with a sat. NH_4Cl and washed with sat. $NaHCO_3$). 24 h were found to be result in an optimum conversion to the desired product and upon completion, the reaction was quenched with aq. sat. NH_4Cl (25 mL) and transferred into a conical flask and cooled to 0 ºC. To this cold biphasic mixture, aq. sat. $NaHCO_3$ (50 mL) was slowly added and the organics were extracted into EtOAc (2 × 100 mL) washed with brine (50 mL), dried (Na_2SO_4) and concentrated *in vacuo*. The crude concentrate was purified using normal-phase column chromatography (40 g silica; 0→30% EtOAc: Hexane) to result in **FC11** (28 mg, 25%) as yellow viscous liquid: ^{1}H NMR (500 MHz, $CDCl_3$) δ 8.36 (d, *J* = 3.0 Hz, 1H), 7.47 (dt, *J* = 3.5, 1.5 Hz, 1H), 6.19 (m, 1H), 3.94 – 3.85 (m, 4H), 3.40 – 3.32 (m, 4H), 2.43 (d, *J* = 1.5 Hz, 3H). ^{13}C {^{1}H} NMR (125 MHz, $CDCl_3$) δ 186.0(t, $J_{C\text{-}F}$ = 24 Hz), 157.8 (t, $J_{C\text{-}F}$ = 3 Hz), 146.8, 139.7 (t, $J_{C\text{-}F}$ = 5 Hz), 138.0 (t, $J_{C\text{-}F}$ = 1 Hz), 130.1 (t, $J_{C\text{-}F}$ = 24 Hz), 121.7 (t, $J_{C\text{-}F}$ = 3 Hz), 118.2 (t, $J_{C\text{-}F}$ = 3 Hz), 105.6 (t, $J_{C\text{-}F}$ = 245 Hz), 66.3, 47.1, 19.6. ^{19}F NMR (376 MHz, $CDCl_3$) –101.65 (d, $J_{H\text{-}F}$ = 4 Hz). HRMS (ESI) m/z: $[M+H]^+$ Calcd. for $C_{14}H_{15}F_2N_2O_2$ 281.1096; Found 281.1093.

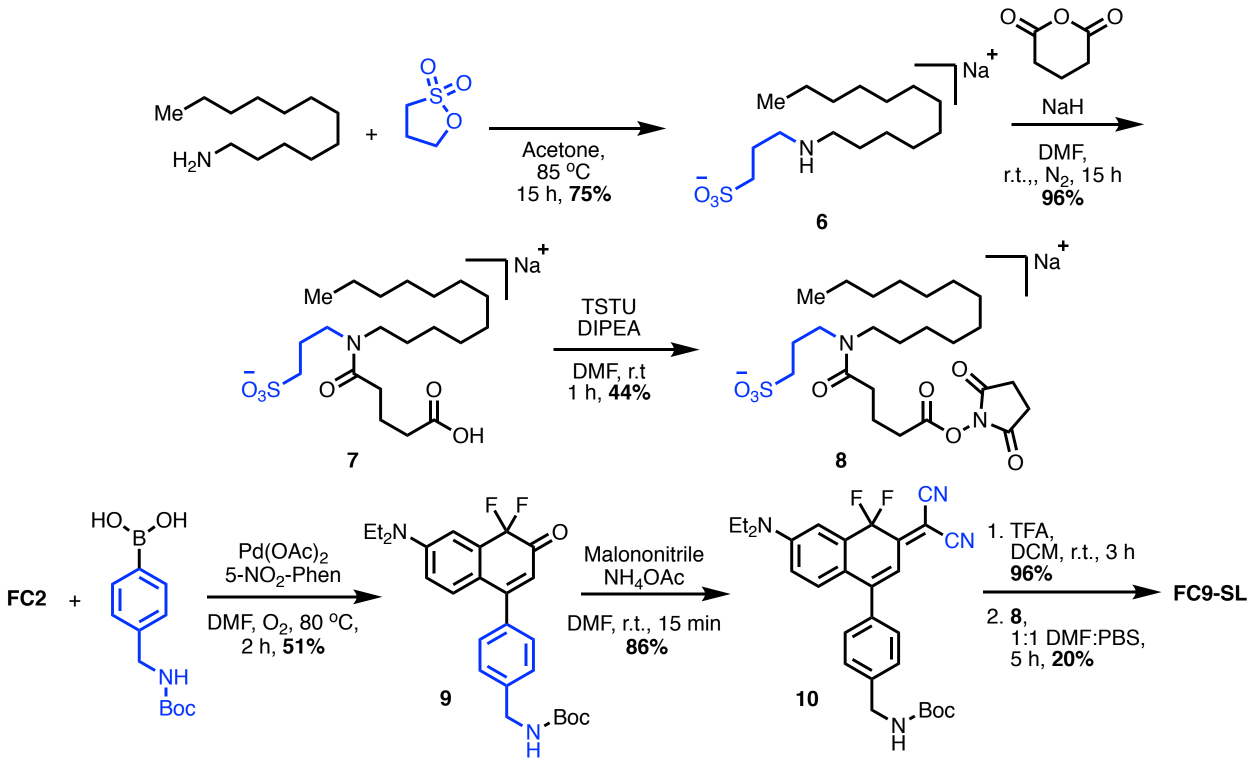

Scheme S1: Synthesis of **FC9-SL**

5-(dodecyl(3-sulfopropyl)amino)-5-oxopentanoic acid (7):

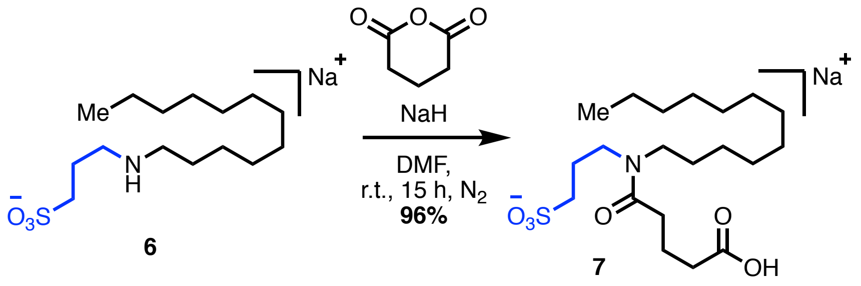

To a cooled (0 ºC in an ice bath) solution of 3-(dodecylamino) propane-1-sulfonic acid **6** (200 mg, 0.608 mmol), synthesized according to a previously reported literature procedure,[4] and glutaric anhydride (149 mg, 1.31 mmol) in DMF was added NaH (31.36 mg, 1.307 mmol) in ~ 5 batches under N_2. The reaction was then sealed under N_2 and allowed to stir at r.t. for 15 h. The progress of the reaction was monitored by LC/MS and a clean conversion to the desired product (m/e = 420.2 ($[M–H]^-$) was seen after 15 h. After this time, the reaction mixture was added to acetone (15 mL) in a centrifuge tube and a solid precipitate crashed out. This was then centrifuged, and supernatant was decanted, following which, the precipitate was washed with acetone (15 mL). This procedure was repeated twice and the solid was dried under high vaccum for at least 2 h to result in **7** (259 mg, 96%) as a beige colored solid: ^1H NMR (400 MHz, D_2O) δ 3.38 (m, 2H), 3.29 (m,

2H), 2.87 – 2.74 (m, 2H), 2.37 (m, 2H), 2.27 (t, J = 7.6 Hz, 2H), 2.01 – 1.86 (m, 2H), 1.80 (p, J = 7.2 Hz, 2H), 1.48 (d, J = 22.0 Hz, 2H), 1.30 – 1.15 (m, 18H), 0.85 – 0.76 (m, 3H). HRMS (ESI) m/z: $[M+H]^+$ Calcd. for $C_{20}H_{39}NO_6S$ 422.2571; Found 422.2569.

5-(dodecyl(3-sulfopropyl)amino)-5-oxopentan-succinamidyl ester (8):

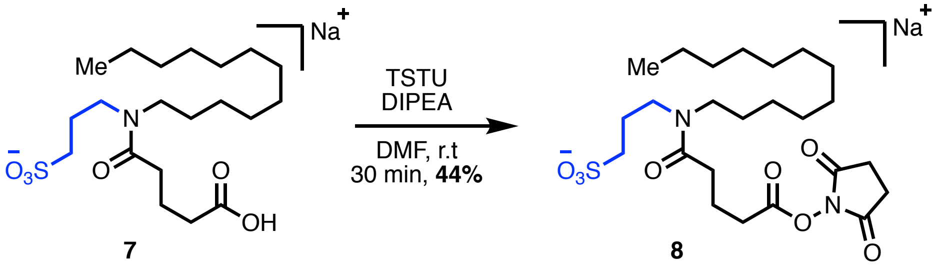

7
8

To a mixture of 5-(dodecyl(3-sulfopropyl)amino)-5-oxopentanoic acid **7** (65 mg, 0.147 mmol) and TSTU (88 mg, 0.293 mmol) dissolved in DMF (1 mL) was added DIPEA (38 mg, 0.293 mmol). After 1 h at room temperature complete conversion to the NHS ester was observed by LC/MS. The reaction mixture was then added to diethyl ether (2 mL) resulting in a pale brown solid precipitate crashing out. This was then centrifuged, and supernatant was decanted, following which, the precipitate was washed with ether (15 mL). This procedure was repeated twice and the solid was dried under high vaccum for at least 30 min to result in **8** (35 mg ,44%) as a pale brown colored solid that was immediately used without further purification. HRMS (ESI) m/z: $[M+H]^+$ Calcd. for $C_{24}H_{43}NO_8S$ 519.2735; Found 519.2736.

Tert-butyl (4-(6-(diethylamino)-4,4-difluoro-3-oxo-3,4-dihydronaphthalen-1-yl)benzyl)carbamate (9):

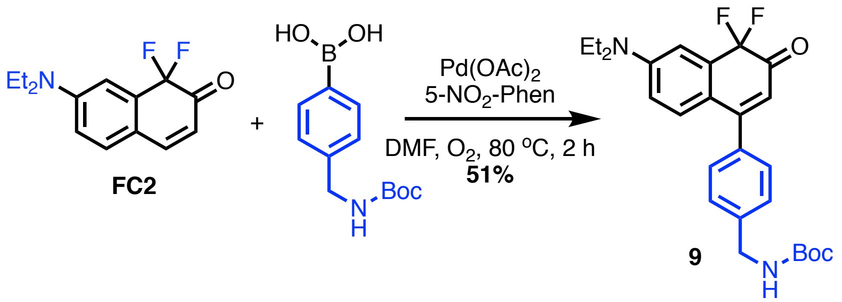

FC2
9

A modified literature procedure was followed.[2] 7-(diethylamino)-1,1-difluoronaphthalen-2(1H)-one (**FC2**) (48 mg, 0.19 mmol) , phenyl boronic acid (26 mg, 0.38 mmol), palladium acetate (41 mg, 0.18 mmol) and 5-NO_2-1,10-phenanthroline (41 mg, 0.18 mmol) were sealed in a microwave vial. This reaction vessel was purged with O_2, following which DMF (2.5 mL) was added. The solvent was further purged with O_2 for 10 minutes and then the reaction mixture was heated to 80

°C. The progress of the reaction was monitored using LC/MS and TLC and upon completion (2 h), water (5 mL) was added to the reaction mixture. The organics were extracted using EtOAc (20 mL). The organic fraction was washed with H_2O (2 × 20 mL) and brine (2 × 20 mL) then dried (Na_2SO_4) and concentrated *in vacuo*. The crude mixture was purified using normal-phase column chromatography (12 g silica; 0→5 – 10% EtOAc: Hexane) to result in **9** (44 mg, 51%) as a thick viscous dark red liquid: HRMS (ESI) m/z: $[M+H]^+$ Calcd. for $C_{26}H_{30}F_2N_2O_3$ 457.2297; Found 457.2295.

Tert-butyl (4-(3-(dicyanomethylene)-6-(diethylamino)-4,4-difluoro-3,4-dihydronaphthalen-1-yl) benzyl)carbamate (10):

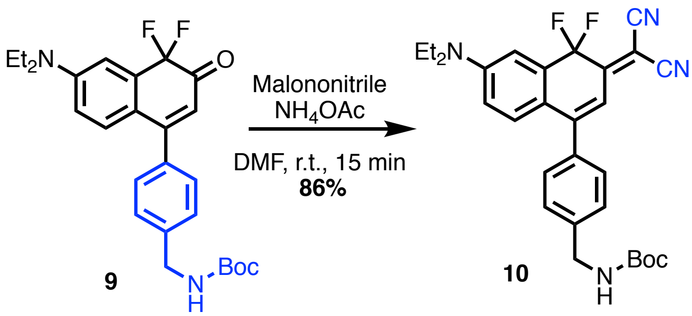

To a mixture of tert-butyl (4-(6-(diethylamino)-4,4-difluoro-3-oxo-3,4-dihydronaphthalen-1-yl)benzyl)carbamate **9** (40 mg, 0.088 mmol) and malononitrile (23 mg, 0.35 mmol) in DMF was added NH_4OAc (7 mg, 0.088 mmol), in one batch, and stirred at r.t. for 15 min. The progress of the reaction was monitored by LC/MS and after the complete consumption of the product, the reaction as quenched with $NaHCO_3$ (10 mL). The contents were transferred into a separating funnel and the organics were extracted using EtOAc (2 × 20 mL). The organic fraction was washed with H_2O (2 × 20 mL) and brine (2 × 20 mL) then dried (Na_2SO_4) and concentrated *in vacuo*. The crude mixture was purified using normal-phase column chromatography (4 g silica; 0→2% MeOH: DCM) to result in **10** (38 mg, 86%) as a navy-blue amorphous powder: ^1H NMR (400 MHz, $CDCl_3$) δ 7.41 – 7.38 (m, 4H), 7.20 (m, 1H), 7.20 – 7.16 (m, 1H), 6.59 (dd, J = 9.2, 2.8 Hz, 1H), 6.48 (t, J = 3.2 Hz, 1H), 4.95 (s, 1H), 4.40 (d, J = 6.0 Hz, 2H), 3.48 (q, J = 7.2 Hz, 4H), 1.49 (s, 9H), 1.23 (d, J = 7.2 Hz, 6H). ^{13}C {^1H} NMR (100 MHz, $CDCl_3$) δ 157.4 (t, $J_{C\text{-}F}$ = 21 Hz), 156.0, 153.5, 149.8, 141.2, 134.2 (t, $J_{C\text{-}F}$ = 23 Hz), 131.9, 130.1, 128.9, 127.6, 117.0 (m, $J_{C\text{-}F}$), 115.2 (m, $J_{C\text{-}F}$), 113.0 (m, $J_{C\text{-}F}$), 112.9 (m, $J_{C\text{-}F}$), 112.3, 112.0, 111.0 (m, $J_{C\text{-}F}$), 78.5 (m, $J_{C\text{-}F}$), 44.9, 44.3, 29.7, 28.4, 12.5. ^{19}F NMR (376 MHz, $CDCl_3$) –80.96. HRMS (ESI) m/z: $[M+H]^+$ Calcd. for $C_{29}H_{30}F_2N_4O_2$ 505.2410; Found 505.2402.

Synthesis of FC9-SL:

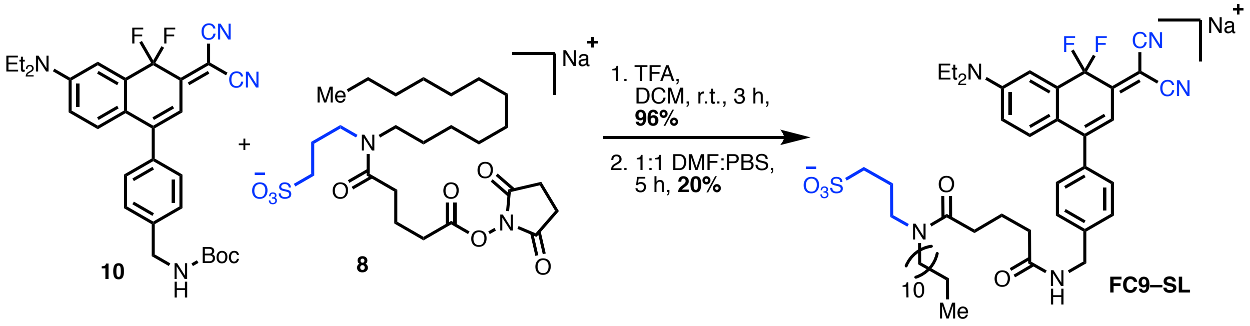

Step-1 (Boc deprotection):

To a solution of tert-butyl (4-(3-(dicyanomethylene)-6-(diethylamino)-4,4-difluoro-3,4-dihydronaphthalen-1-yl)benzyl) carbamate **10** (35 mg, 0.069 mmol) in DCM (1 mL) was added trifluoroacetic acid (100 µL) and allowed to stir at r.t. for 3 h. The progress of the reaction was monitored by LC/MS to find a clean conversion. Upon completion (3 h), the contents were concentrated using N_2 flush and dried *in vacuo* to result in boc-deprotected analog **10** (27 mg, 96%) as a viscous blue liquid, which was taken forward without further purification.

Step-2 (Conjugating the sulfo lipid 8)[c]:

The boc-deprotected analog (27 mg, 0.067 mmol) from step-1 and 5-(dodecyl(3-sulfopropyl)amino)-5-oxopentan -succinamidyl ester **8** (34 mg, 0.063 mmol) was dissolved in a solution of 1:1 PBS (pH = 7.4):DMF (2 mL). The reaction was allowed to stir at r.t. for 5 h, after which time LC/MS analysis showed a clean conversion to the desired product. The reaction mixture was concentrate down (using N_2 flush) to as minimum solvent as possible, dissolved in DCM (~1 mL), and the crude concentrate was purified using normal-phase column chromatography (4 g silica; 0→20% MeOH: DCM) to result in **FC9-SL** (11 mg, 20%). HRMS confirmed the identify of **FC9-SL.** HRMS (ESI) m/z: $[M+H]^+$ Calcd. for $C_{44}H_{59}F_2N_5O_5S$ 808.4278; Found 808.4273.

[c] NMR spectroscopy was not useful due complexity by the amide rotamers and C-F splitting.

Section S3: Optical Characterization of FC dyes and R1–R3

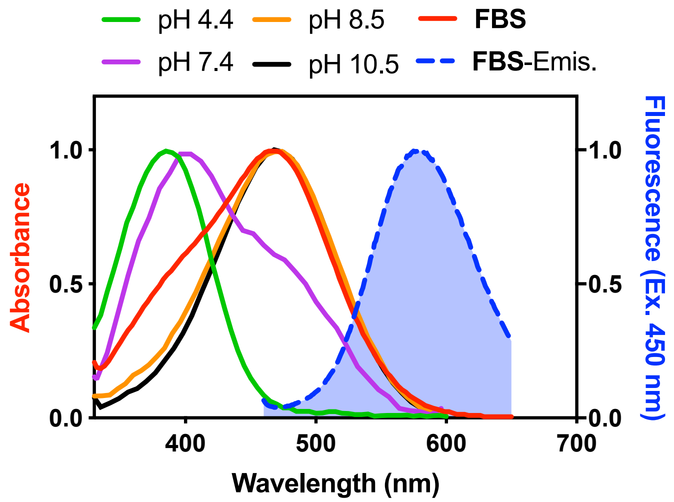

Figure S1: Absorbance of **FC1** in pH 4.4, 7.4, 8.5, 10.5 and FBS and the emission of **FC1** in FBS, while only negligible emission was seen in other solvents

S3.1 Hammett Analysis[d]

We hypothesized that introducing electron withdrawing functionalities would lead to red-shifted **FC** dyes. Surprisingly, a modest red-shift was observed in the case of **FC3** (22 nm) in both hydrophilic and hydrophobic solvents. **FC4** also showed a small red shift (~32 nm) in its spectra which could be attributed to the relatively weak inductive property of the bromo substituent. While, expectedly, the introduction of a –Ph group (**FC5**) did not result in any significant changes compared to **FC2**, **FC6** with –CN group at position-4 resulted in a significantly red-shifted absorption (~70 nm) and emission (~70 nm) spectra in all the solvents investigated. Interestingly, our Hammett analysis (**Figure S2**) shows that this observation was partially correlatable with Hammett constants (except **FC3**) demonstrating the importance of electron-withdrawing groups at position-4.[5] The anomaly found in the case of **FC3** suggests that the modifications at position-3 is not crucial for red-shifting the **FC** dyes.

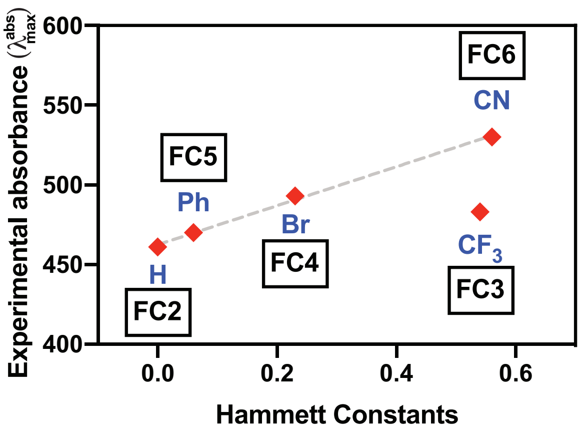

Figure S2. Correlation between Hammett constants of substituents at position -3 and -4 on **FC2** and the corresponding absorbance maxima in 10% FBS

---

[d] Drawing similarities of FC dye scaffold with naphthalene core, σ_p and σ_m Hammett constants were used for substituents at 3- and 4-positions respectively.

Table S1: Optical data of the synthesized **FC** dyes and the reference dyes **R1–R3**

Entry	λ_{max}^{abs} **(nm)** MeCN	λ_{max}^{em} **(nm)** MeCN	**ϵ ($M^{-1}cm^{-1}$)** MeCN	**Stokes shift**		
				in Toluene	in MeCN	in 10% FB[illegible]
**FC1**	378	516	7,900	109	138	111
**FC 2**	476	609	12,500	104	133	84
**FC 3**	496	607	15,500	84	111	72
**FC 4**	507	635	13,600	104	128	84
**FC 5**	481	616	8,900	100	135	92
**FC 6**	542	683	10,700	106	141	70
**FC 7**	470	634	14,100	111	164	85
**FC 8**	611	780	9,800	144	169	110
**FC 9**	620	779	8,400	135	159	117
**FC 10**	680	902	10,900	102	222	113
**FC 11**	416	592	14,500	143	176	109
**R1**	364	436	25,100	55	72	62
**R2**	368	433	24,500	48	65	63
**R3**	475	504	34,000	57	29	35

S3.2 Discussion of optical properties of FC dyes

In the best interest of the reader, we have limited our discussion on the optical properties of FC dyes in the main manuscript to only toluene (mimicking hydrophobic cellular compartments) and 10% FBS (biologically relevant media). Below, we summarize other notable observations that were made.

1. **FC10** exhibited the largest (Δ λ_{max}^{abs} = 205 nm) bathochromic shift in λ_{max}^{abs} relative to its reference dye (**R3**), while **FC7** showed the least (Δ λ_{max}^{abs} = –5 nm) (Figure S3). Only **FC1** had a significant difference in bathochromic shift of λ_{max}^{abs} (cf. **R1**) within the solvents analyzed. In the case of λ_{max}^{em}, all dyes (except **FC1**) demonstrated higher bathochromic shifts in MeCN with **FC10** being the most striking FC dye with Δ λ_{max}^{em} between **FC10** and **R3** 398 nm (Figure S4). This observation signifies the importance of combining the effect of 2-malonodinitrile and 4-nitrile on the FC-scaffold.
2. While the effect of solvent polarity on their λ_{max}^{abs} with **FC2–FC5** and **FC11** (Δ λ_{max}^{abs} < 10 nm shift) was insignificant, **FC1**, **FC6–FC10** exhibit positive solvatochromism (toluene → MeCN → 10% FBS) in their λ_{max}^{abs} (Figure S5). This is indicative of a large asymmetric push-pull character introduced from the electron-withdrawing groups.
3. In the case of emission, positive solvatochromism was seen for all FC dyes going from toluene → MeCN (Figure S6). However, negative solvatochromism between MeCN → 10% FBS was recorded in all cases (except **FC1**). This trend has been reported to be significant in the case of Nile red[8] and, less dramatically, the case of COUPY dyes.[9]

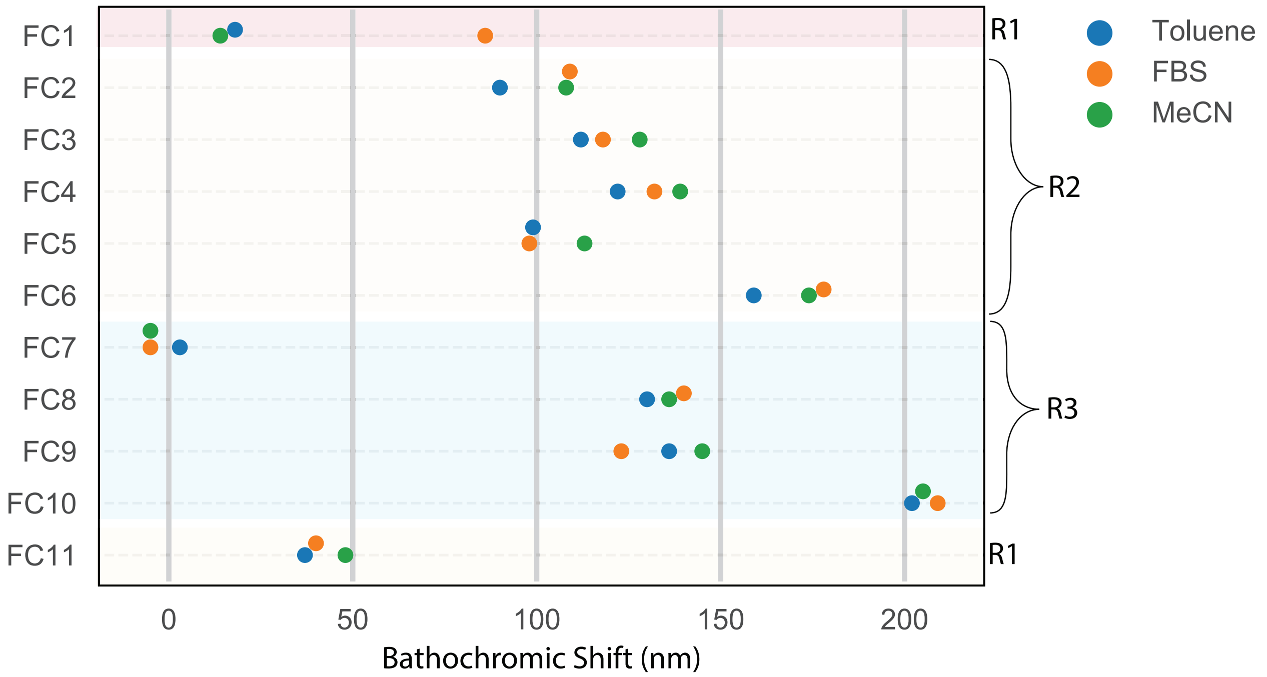

Figure S3. Comparison of bathochromic shift in λ_{max}^{abs} of FC dyes with respect to their corresponding reference dyes **R1–R3** in different solvents (shown)

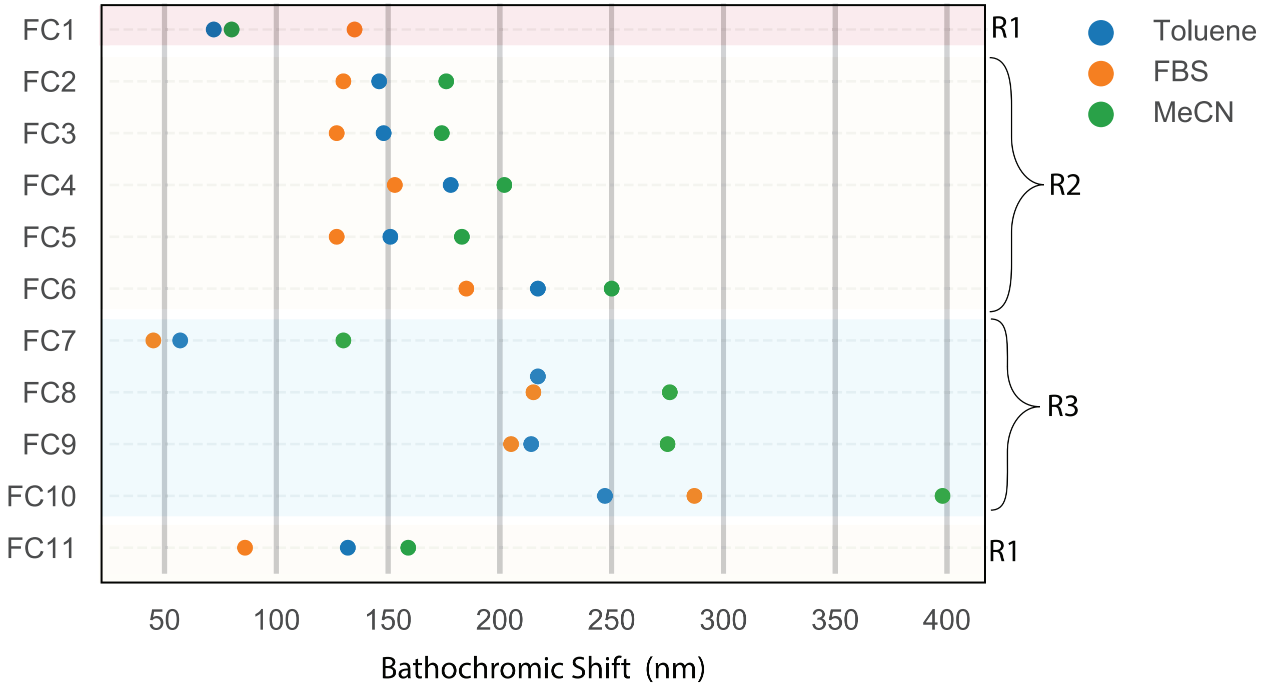

Figure S4. Comparison of bathochromic shift in λ_{max}^{em} of FC dyes with respect to their corresponding reference dyes **R1–R3** in different solvents (shown)

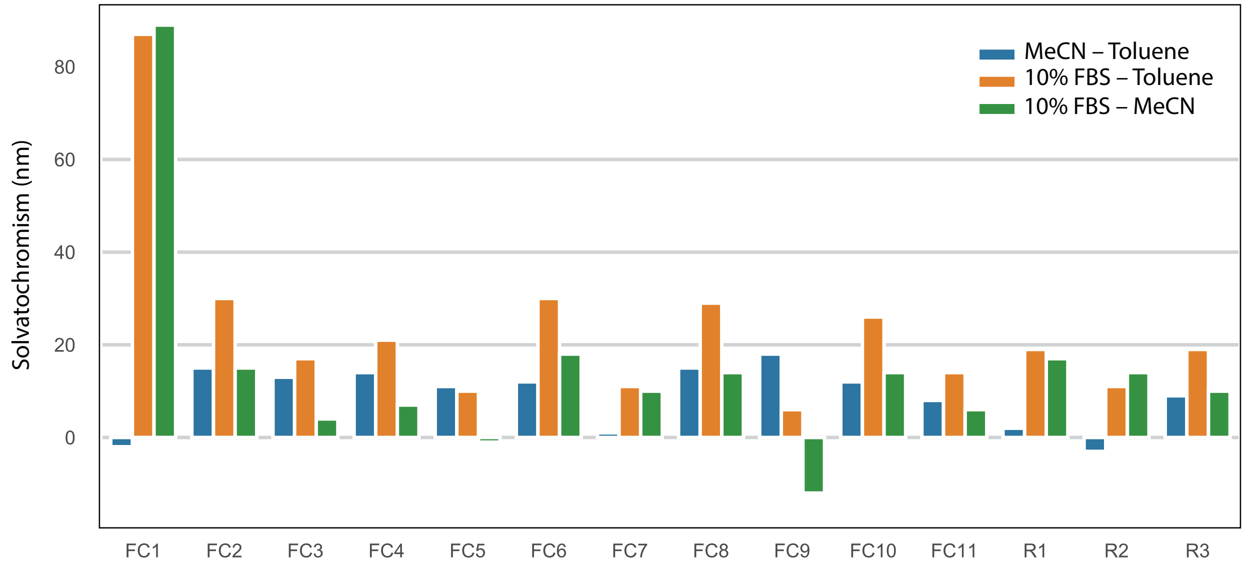

Figure S5. Solvatochromic behavior of the λ_{max}^{abs} of FC dyes upon indicated solvent transitions

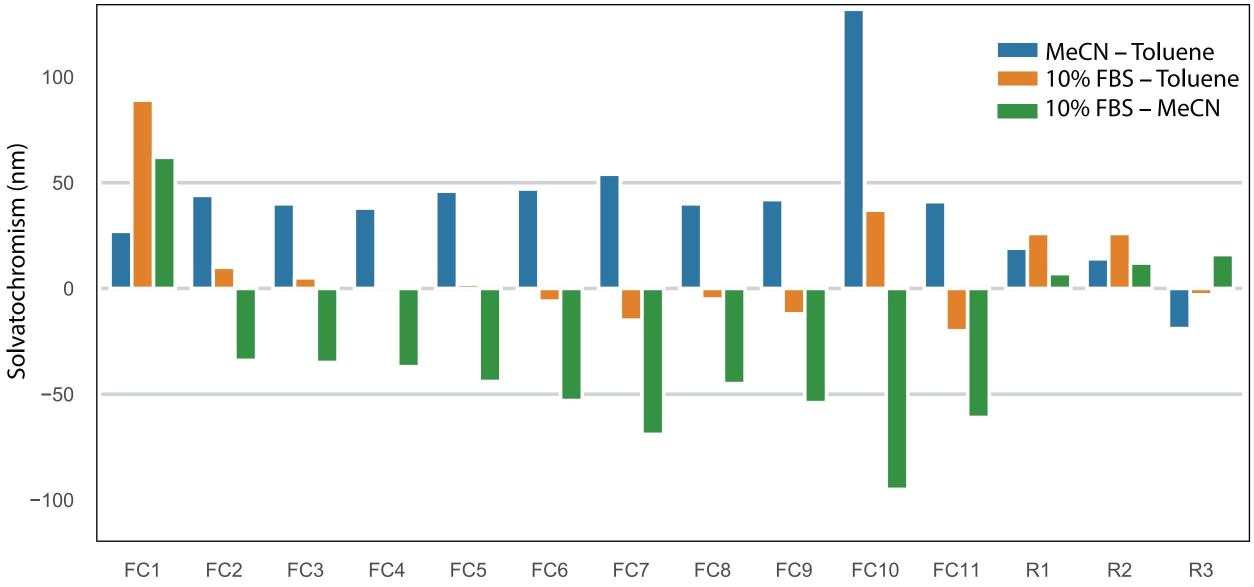

Figure S6. Solvatochromic behavior of the λ_{max}^{em} of FC dyes upon indicated solvent transitions

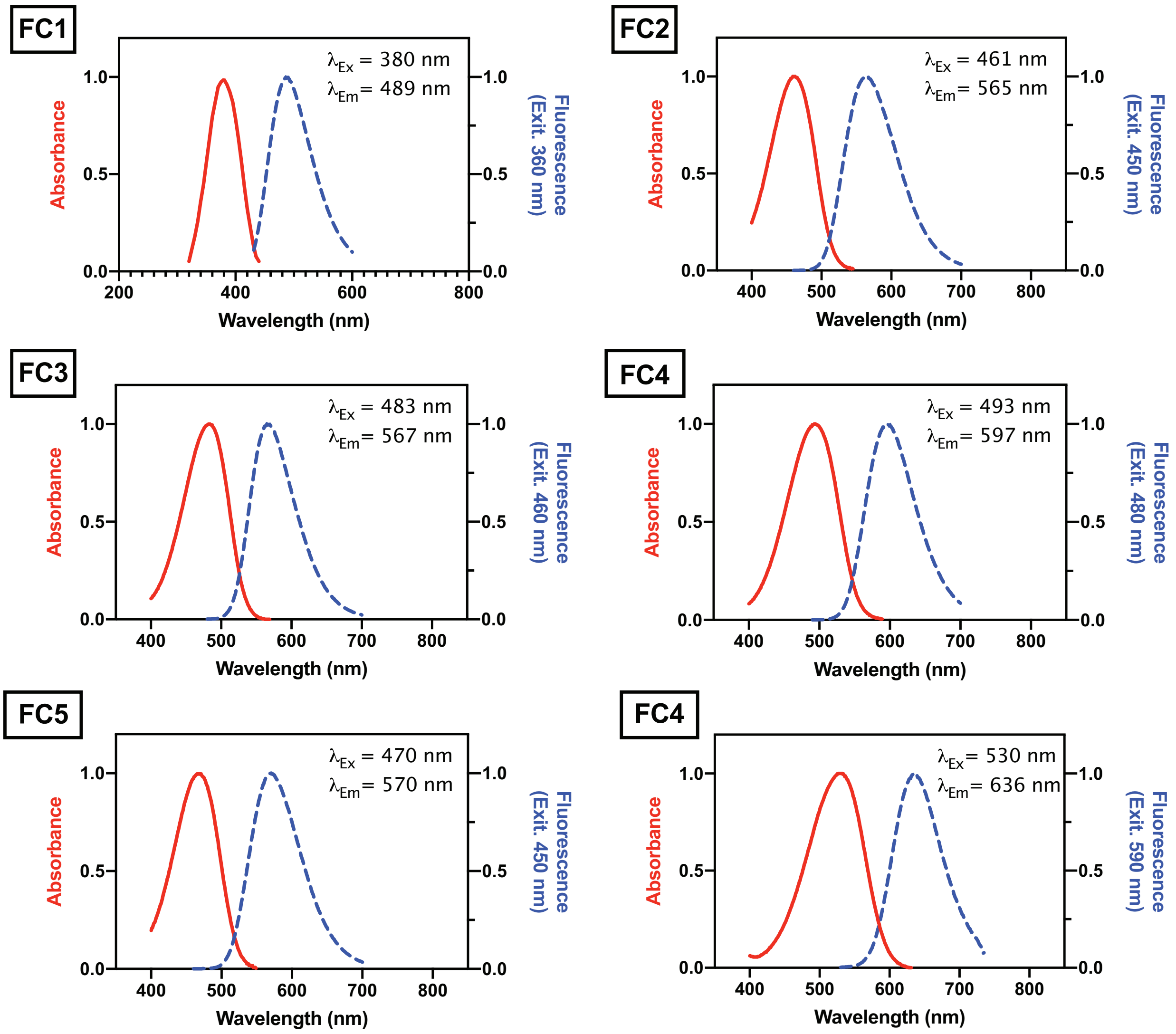

Figure S7: Absorption and emission spectra of dyes **FC1–FC6** in toluene

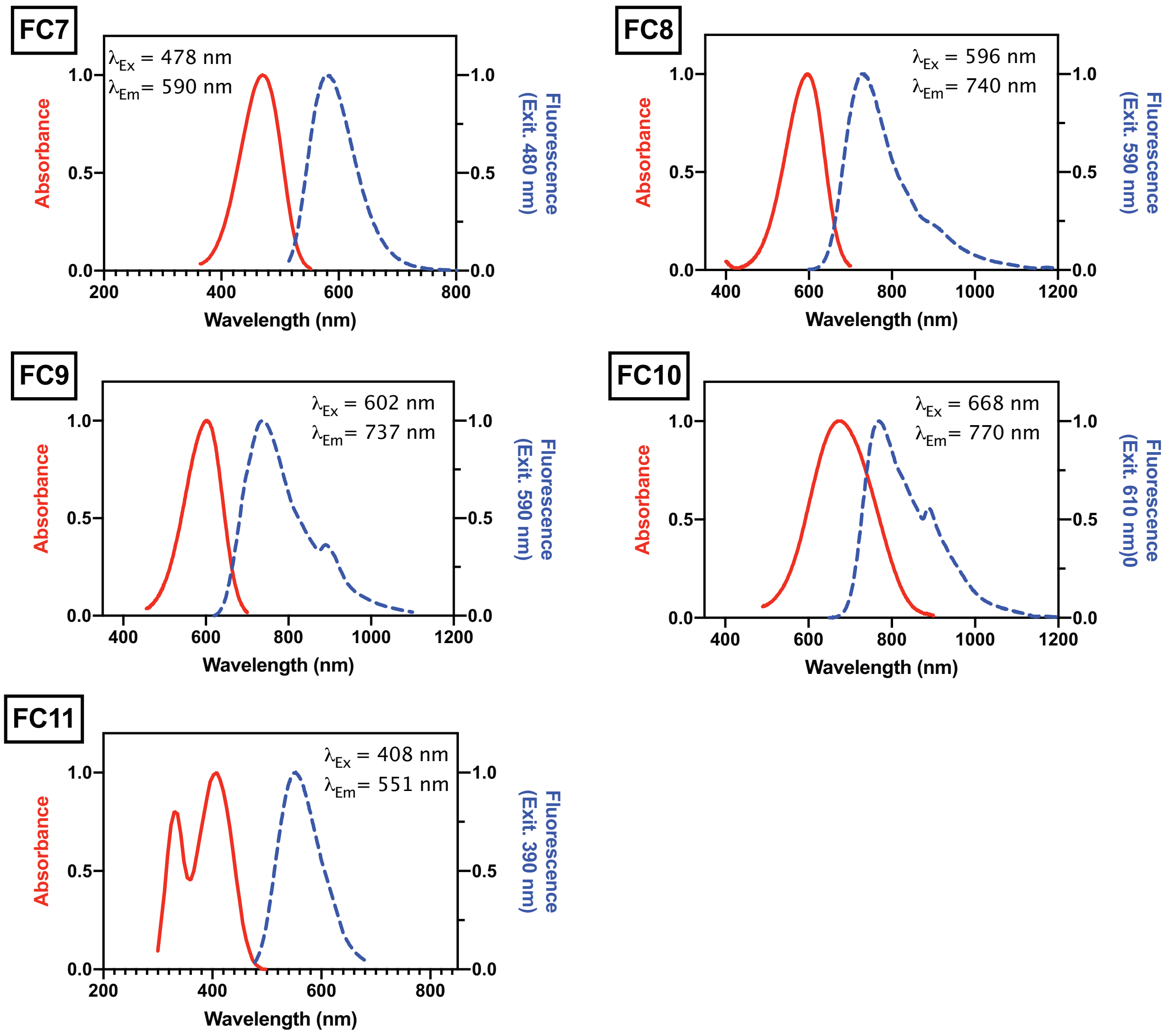

Figure S8: Absorption and emission spectra of dyes **FC7–FC11** in toluene

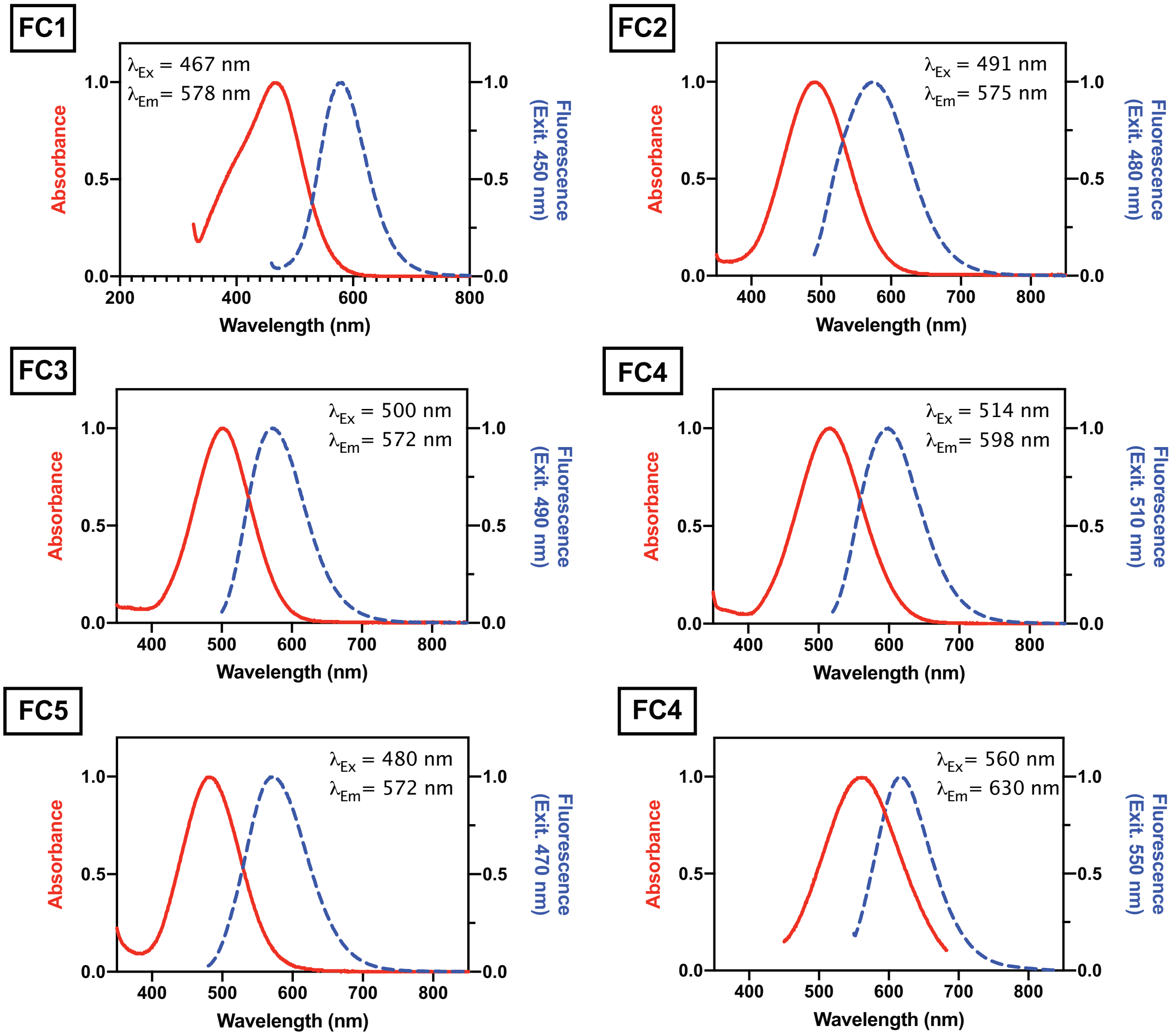

Figure S9: Absorption and emission spectra of dyes **FC1–FC6** in 10% FBS

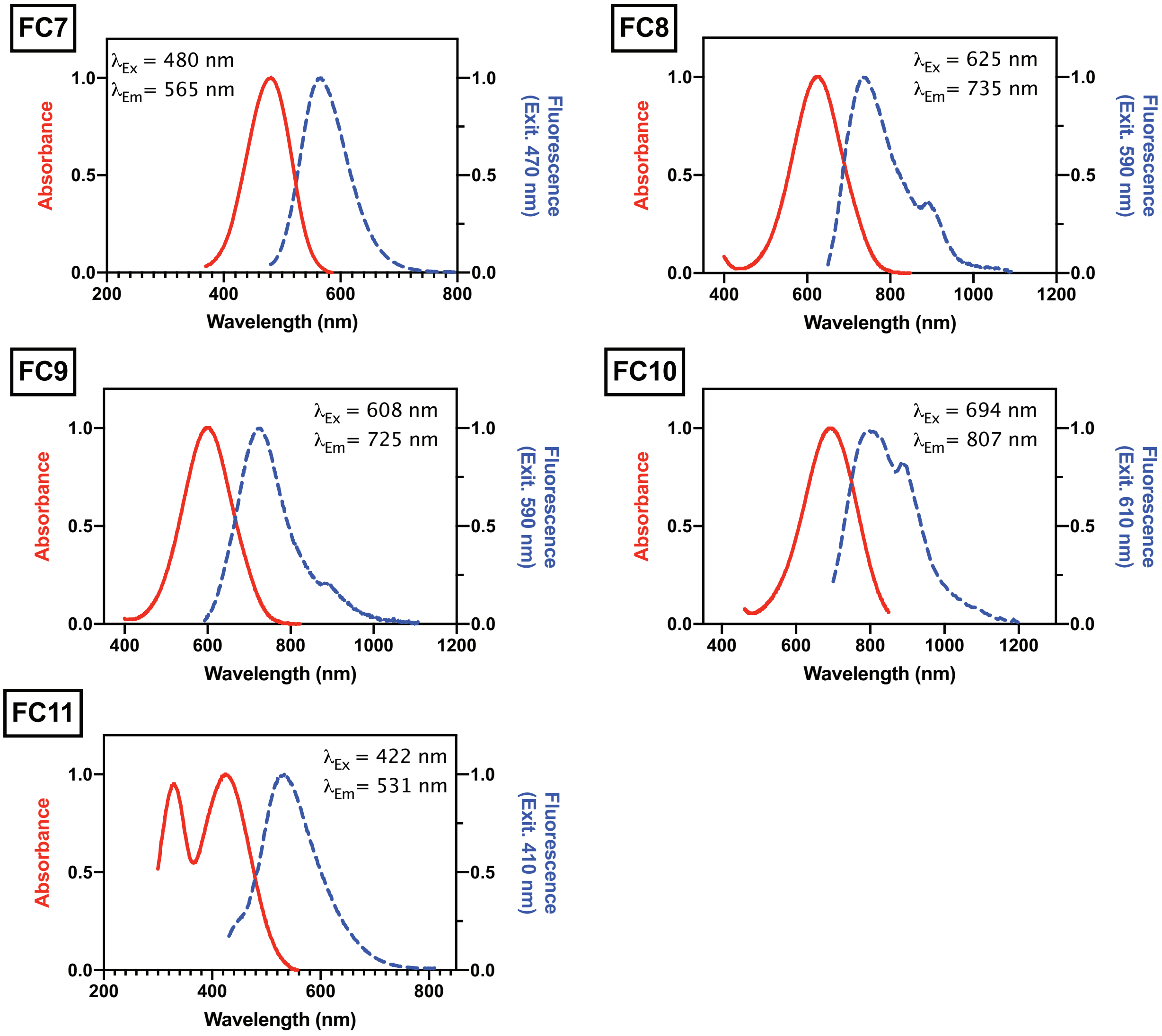

Figure S10: Absorption and emission spectra of dyes **FC7–FC11** in 10% FBS

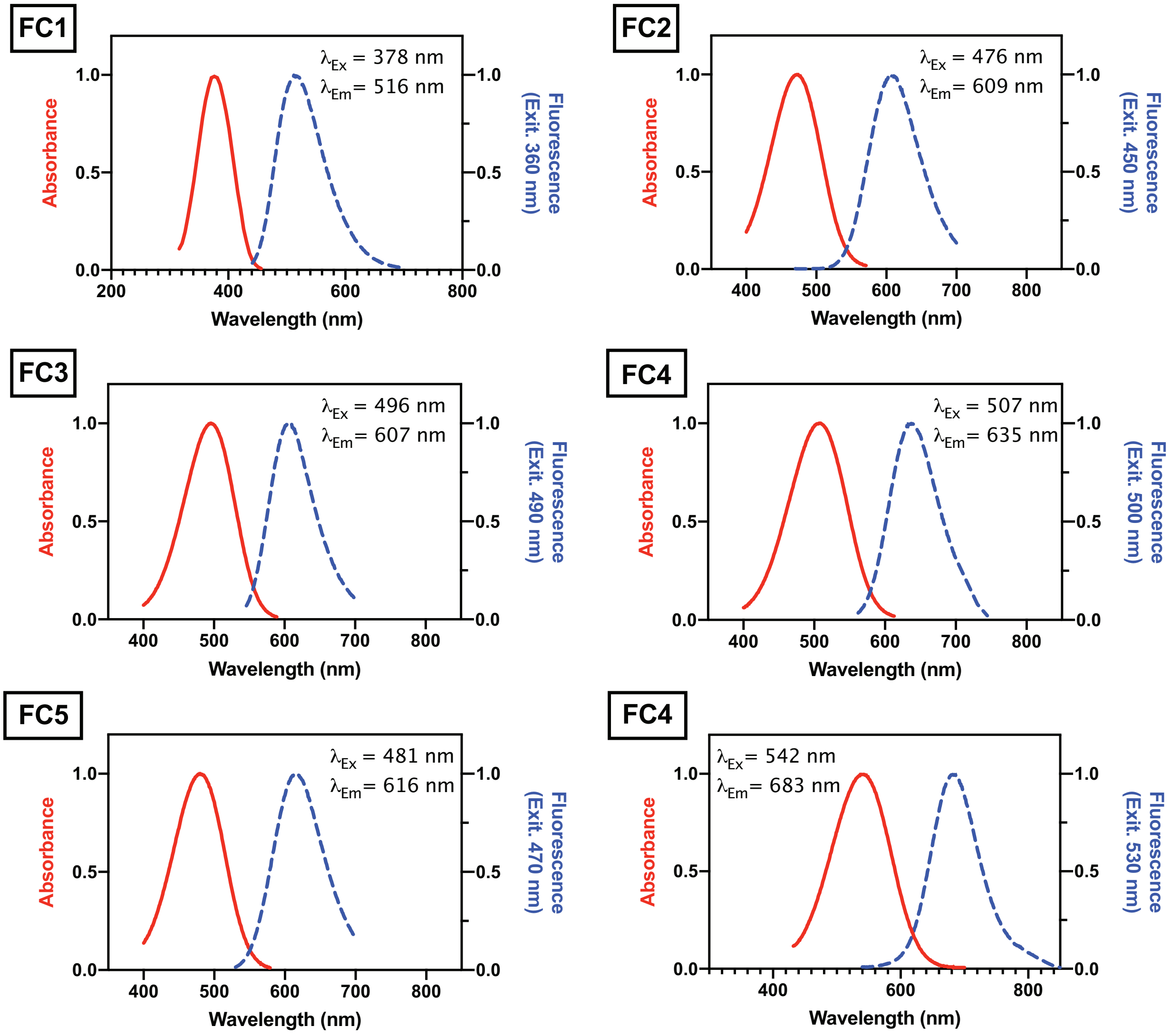

Figure S11: Absorption and emission spectra of dyes **FC1–FC6** in MeCN

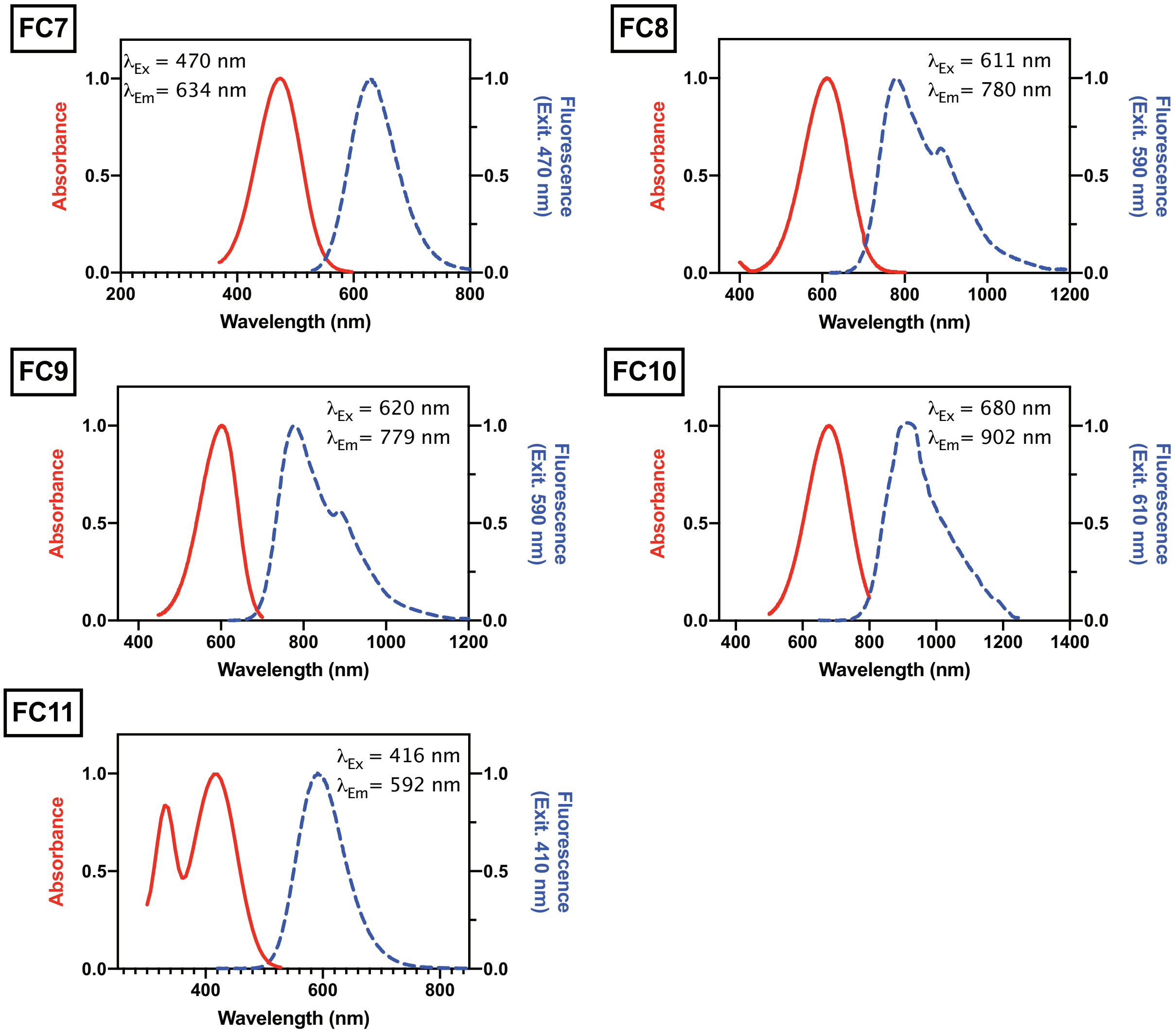

Figure S12: Absorption and emission spectra of dyes **FC7–FC11** in MeCN

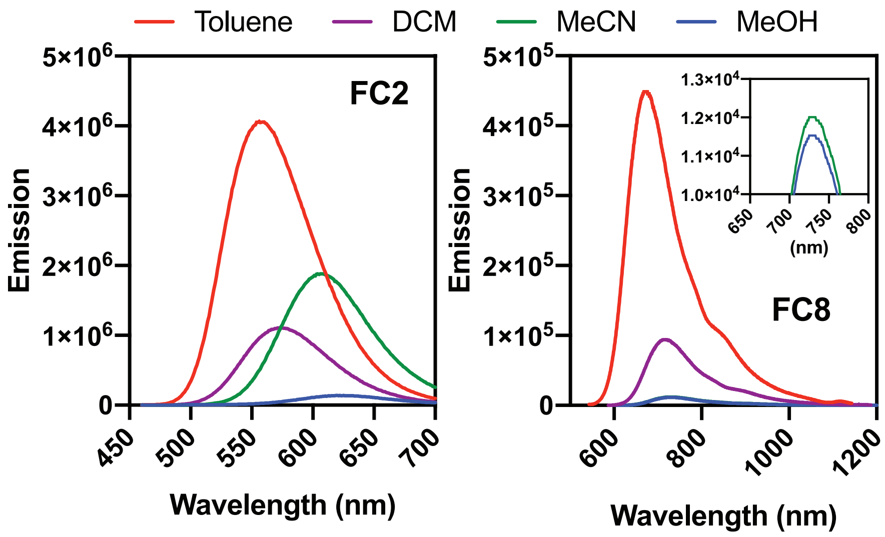

Figure S13: Environmental sensitivity of representative fluorocoumarins **FC2** and **FC8**

Table S2: List of selected small molecule fluorophores

Entry	Structure	Molecular weight	λ_{max}^{abs} (nm) (solvent)	λ_{max}^{em} (nm) (solvent)
1[a]	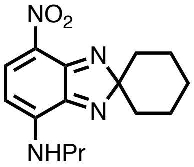	288.15	546 (EtOH)	650 (EtOH)
2	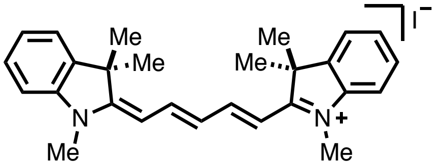	510.15	638 (MeOH)	657 (MeOH)
3	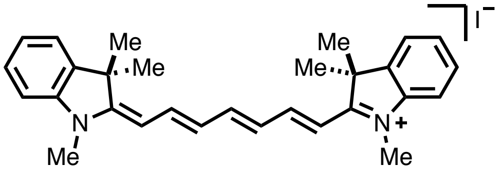	536.16	740 (MeOH)	772 (MeOH)
4	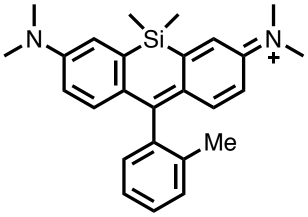	399.22	646 (PBS)	660 (PBS)
5	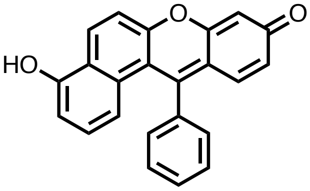	338	591 (PBS)	757 (PBS)
6[b] **(FC8)**	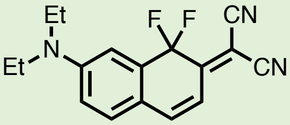	299.12	625 (10% FBS)	735 (10% FBS)
7[b] **(FC10)**	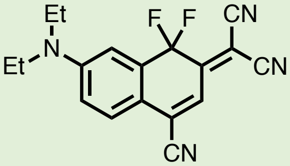	324.11	694 (10% FBS)	807 (10% FBS)

[a] The nitrobenzoxadiazole variant reported to be the smallest fluorophore emitting in the 650–900 nm range.[10]

[b] Smallest dyes with λ_{max}^{abs} over 600 nm and λ_{max}^{em} above 700 nm.

Section S4: Photostability studies: The photostability of the **FC** dyes was investigated in toluene and MeCN upon irradiation with an appropriate LED source set to an intensity of 10 mW/cm^2. The FC dyes were diluted to 25 µM, from a principal stock solution of 10 mM (in DMSO), in a HPLC vial. In general, all the **FC** dyes (except **FC1** and **FC7**) were photostable in toluene and MeCN (~ 30 – 50% degradation over 60 min; Figure S14). Dinitrile-substituted dyes (**FC8 – FC10)** were highly photostable upto 24 h of irradiation. Particularly, **FC10** upon irradiation with a 660 nm LED at an intensity of 50 mW/cm^2 for 8 h was found to be > 80% intact. Dark stability of these dyes was recorded, in parallel, to find that all the **FC** dyes were stable for the duration of the study in the absence of irradiation.

LEDs used:

- 365 nm: **FC1**, **FC11**
- 450 nm: **FC5**, **FC7**
- 480 nm: **FC2**
- 505 nm: **FC3**, **FC4**
- 525 nm: **FC6**
- 590 nm: **FC8**, **FC9**
- 660 nm: **FC10**

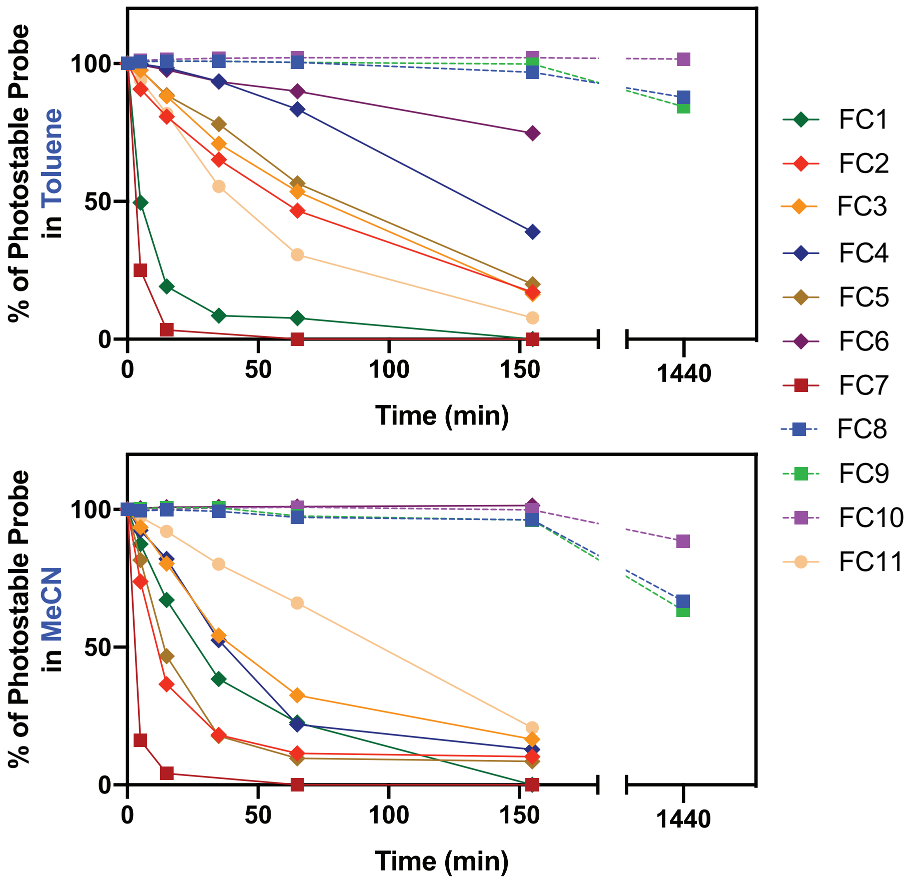

Figure S14. Photostability of the **FC** dyes in toluene and MeCN

Section S5: Cell Culture

MCF7 human breast cancer cell line was obtained from ATCC®. They were cultured at 37 °C in an atmosphere of 20% O_2 and 5% CO_2 in DMEM supplemented with 4 mM L-glutamine, 25 mM D-glucose, 44 mM sodium bicarbonate, and 10% heat-inactivated fetal bovine serum. Stock cultures were maintained in a continuous exponential growth by weekly passage of the appropriate number of cells following trypsinization with 0.25% Trypsin-EDTA (0.9 mM) in PBS.

Section S6: Confocal Cellular Imaging

S6.1 General procedure for Passive organelle staining experiments

Cells were seeded into 6-well plates at a density of 8×10^3 cells/well and allowed to reach near confluency overnight. The following day, media was replaced with phenol red-free DMEM containing 15 μM of FC dyes (diluted from a 10 mM stock) [and 2 μM BODIPY493/503 or To-Pro™-iodide for co-localization experiments, (see discussion below)], and incubated for 30 min. In the case of **FC2–FC10**, the media was then removed, and the cells were incubated in DMEM with Hoechst (1 μM) for 20 min, to stain the nuclei. After which time, removed the media and washed with HBSS (1×) and visualized in HBSS. The images were processed using Icy software. The Pearson's correlation coefficient was measure with the Colocalization Studio plugin[11] using Icy software.

S6.2 Confocal imaging experiments

Lipid droplet stains[e]: The preliminary study of **FC2–FC11** demonstrated **FC6** and **FC9** showed a selective and intense fluorescence in vesicles in the cellular cytoplasm which apparently looked like lipid droplets. In order to confirm our postulate, a colocalization experiment of **FC9**, was performed using BODIPY 493/503 as a reference LD marker. The results confirmed a clear colocalization between BODIPY 493/503 and **FC9** (Pearson's coefficient = 0.89). Due to an extensive cross talk between **FC6** and other commercial lipid droplet stains, BODIPY 493/503, we used **FC9** was used in combination with **FC6**. These colocalization experiments confirmed that the **FC6** and **FC9** selectively stain the LDs.

---

[e] Note: Red channel (laser: 594 nm, emission filter: 665 – 759 nm), Green Channel (laser: 488 nm, emission filter: 489 – 553 nm), Blue channel (laser: 405 nm, emission filter: 410 – 481 nm).

Nuclear stain[f]: The hydrophilic nature imparted to **FC11** due to the incorporation of the quinoline-like structure prompted us to check its nuclear staining ability. The general procedure was used with **FC11** and To-Pro™-iodide co-incubated with live MCF7 cells for 30 minutes. After which time, removed the media and washed with HBSS (1×) and visualized in HBSS. In the case of control experiment, the reference standard (TO-Pro™-iodide) was not added.

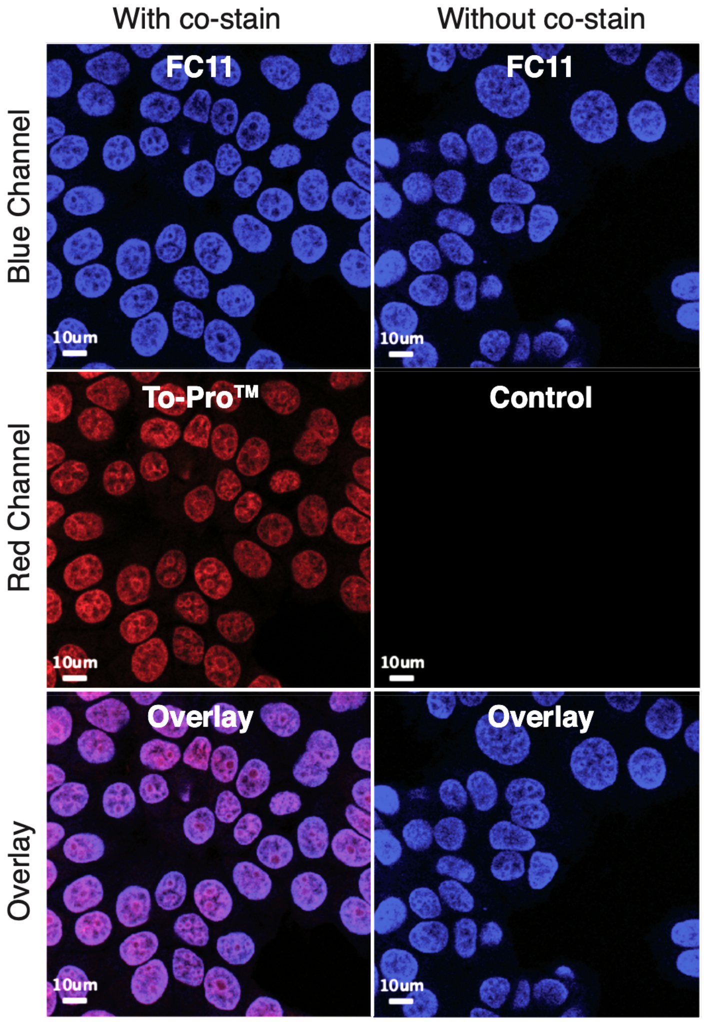

Figure S15: Laser scanning confocal microscopy of live MCF-7 cells incubated for 30 min in phenol red free DMEM in the presence of **FC11** (15 µM, blue channel) and the co-stains To-Pro™ 493/503 (red channel). Scale bar is 10 µm. Pearson's coefficient calculated: 0.88

[f] Note: Blue channel (laser: 405 nm, emission filter: 410 – 481 nm). Red Channel (laser: 594 nm, emission filter: 650 – 759 nm)

S6.3 General procedure for Membrane Staining Experiments[g]

Cells were seeded into 6-well plates at a density of 8 × 10^3 cells/well and allowed to reach near confluency overnight. The following day, media was replaced with phenol red–free DMEM containing the pre-stain (provided along with the MemBrite Fix 488/515 plasma membrane labelling kit from Biotium and prepared as directed) and allowed to incubate at 37 ºC for 5 min. The media was then removed and phenol red-free DMEM containing 15 µM of FC dyes (diluted from a 10 mM stock) and recommended (undisclosed concentration) amount of MemBrite Fix 488/515 was added for co-localization experiments and incubated for 30 min. The media was then removed, and the cells were incubated in DMEM with Hoechst (1 µM) for 20 min, to stain the nuclei. After which time, removed the media, washed with HBSS (1×) and visualized in HBSS. The images were processed using Icy software.

[g] Red channel (laser: 594 nm, emission filter: 665 – 759 nm), Green Channel (laser: 488 nm, emission filter: 489 – 553 nm), Blue channel; laser: 405 nm, emission filter: 410 – 481 nm.

Section S7: Computational Analysis

Quantum Chemical Analysis: The GAMESS package was utilized for all calculations[12, 13] and cc-pVTZ basis sets (spherical harmonics) were employed throughout.[14-16] Density functional theory (DFT)[17] and time-dependent density functional theory (TDDFT)[18-21] methods were used together with the B3LYP functional[22-24] and Euler-MacLaurin radial[25] and Lebedev angular grids[26] with respective dimensions (96, 302) and (48, 110). The polarizable continuum model (PCM)[27-31] approach was used to model water solvent effects and all calculations used a high density of tesserae (NTSALL=960 in $TESCAV). Vertical excitation, or absorption, energies and deexcitation, or emission, energies were computed at ground state and singlet excited state optimized geometries, respectively. Mulliken electronic population analysis[32] was used to examine B3LYP-PCM highest occupied molecular orbitals (HOMOs) and lowest unoccupied molecular orbitals (LUMOs). Molecular orbitals were illustrated with MacMolPlt.[33]

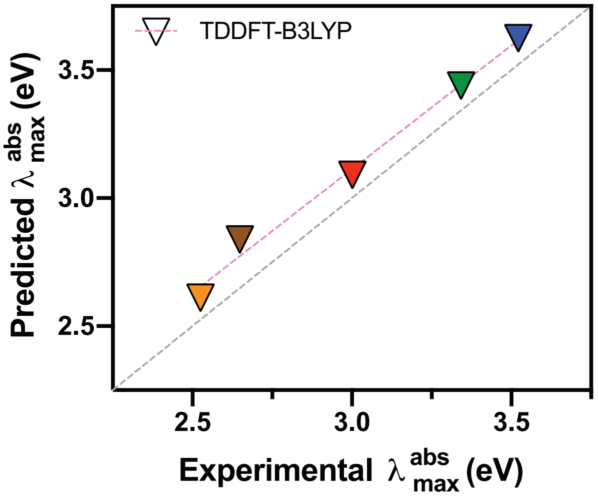

Figure S16: Correlation between predicted and experimental λ_{max}^{abs} using TDDFT-B3LYP-PCM(H_2O).

Table S3: Computed absorbance and emission energies (in eV with oscillator strengths in parentheses) vs. experimentally measured (where available). Calculations included water solvent effects via the Polarizable Continuum Model (PCM). See Methods for full details.

R-group	Computed absorbance	Experimental absorbance	Computed emission	Experimental emission
N(H)	3.624 (0.48)	3.522[34]	3.250 (0.71)	
O	3.439 (0.53)	3.342[34]	3.057 (0.71)	2.621[34]
$C(Me)_2$	3.091 (0.43)	3.002[35]	2.747 (0.61)	2.238[35]
$Si(Me)_2$	2.838 (0.39)[a]	2.649[36]	2.469 (0.48)[b]	1.977[36]
$S(O)_2$	2.622 (0.36)		2.058 (0.32)	
$C(F)_2$	2.611 (0.33)	2.525[c]	2.084 (0.32)	
P(OOEt)	2.603 (0.32)		2.162 (0.37)	

[a] At the ground state (S0) optimized geometry the S1 state has $n \rightarrow \pi^*$ character and this S0 → $S1^*$ transition has a near zero oscillator strength of 0.0016. The S2 state has $\pi \rightarrow \pi^*$ character and corresponding S0 → $S2^*$ oscillator strength of 0.39 so this absorption energy is given.

[b] During the geometry optimization of the S2 state ($\pi \rightarrow \pi^*$, starting from S0 optimized geometry and using small maximum step size of 0.05 Bohr) its energy dropped below the $n \rightarrow \pi^*$ excited singlet state in the very first step, thus becoming the first excited singlet state S1. Subsequent geometry optimization of this S1 $\pi \rightarrow \pi^*$ state led to it transforming to $n \rightarrow \pi^*$ character beginning at step 33, and thus quickly acquiring an essentially zero oscillator strength. Therefore, the $S1^* \rightarrow S0^*$ emission energy given is for step 32 which is the last point that true $\pi \rightarrow \pi^*$ character is retained and where the oscillator strength of 0.48 is still high. See **Figure S18** for more details.

[c] This work.

Table S4: Computed absorption energies (eV), solute dipole moments (Debye) and total R-group Mulliken electron populations in the LUMOs. Properties determined at ground state optimized geometries. Calculations included water solvent effects via the Polarizable Continuum Model (PCM). See Methods for full details. Correlation coefficients (R^2) for computed absorption vs. property are included in italic font at bottom of columns.

R-group	Computed absorbance	Solute dipole moment	LUMO Mulliken e^- populations on R-group
N(H)	3.624	10.05	−0.056[a]
O	3.439	10.35	−0.044[a]
$C(Me)_2$	3.091	9.76	0.058
$Si(Me)_2$	2.838	11.01	0.091
$S(O)_2$	2.622	14.80	0.088
$C(F)_2$	2.611	11.90	0.085
P(OOEt)	2.603	11.95	0.105
$\boldsymbol{R^2}$		***0.50***	***0.92***

[a] These Mulliken populations are penalized to be negative since each R-group introduces a node to the LUMO that passes between the ring-bound R-group atom (not in parentheses) and neighboring carbon atoms, i.e., in these systems the ring-bound R-group atom has a negative bond order with neighboring carbon atoms in the LUMO. For the other molecules, the ring-bound R-group atom has a positive bond order with neighboring carbon atoms in the LUMO. See **Table S5** for further details.

Table S5: Examinations of HOMOs and LUMOs in the coumarin series via Mulliken electronic population analysis and total polar surface area (tPSA). For each orbital, the bond order between C-0 and ring-bound R-group atom is provided along with the total electron population on the R group. Analyses were performed at ground state optimized geometries. tPSA was measured using Chemdraw.

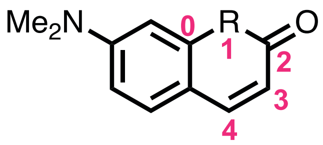

R-group	HOMO		LUMO		tPSA
	C-0−R bond order	R e^- population	C-0−R bond order	R e^- population	
(H)N	0.000	0.000	−0.038	0.056	29.54
O	0.001	0.000	−0.041	0.044	32.34
$C(Me)_2$	−0.002	0.002	0.045	0.058	20.31
$Si(Me)_2$	0.001	0.001	0.054	0.091	20.31
$S(O)_2$	0.000	0.001	0.042	0.088	54.45
$C(F)_2$	0.000	0.001	0.048	0.085	20.31
P(OOEt)	−0.001	0.001	0.050	0.105	46.61

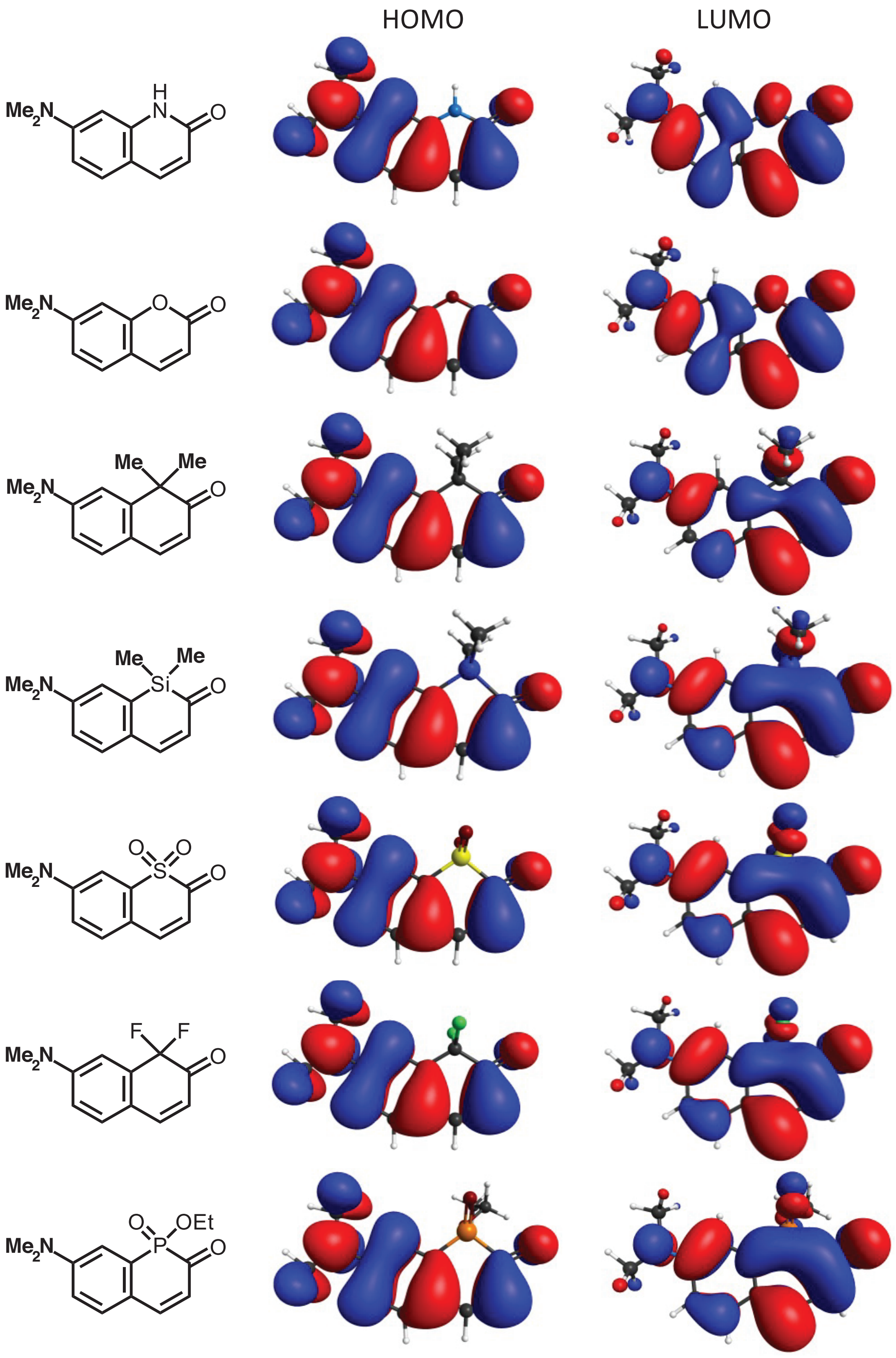

Figure S17: Illustrations of B3LYP-PCM/cc-pVTZ HOMOs (left) and LUMOs (right) for the coumarin systems studied in this work. All plots show contour value of 0.02 $(\text{electron/bohr}^3)^{1/2}$.

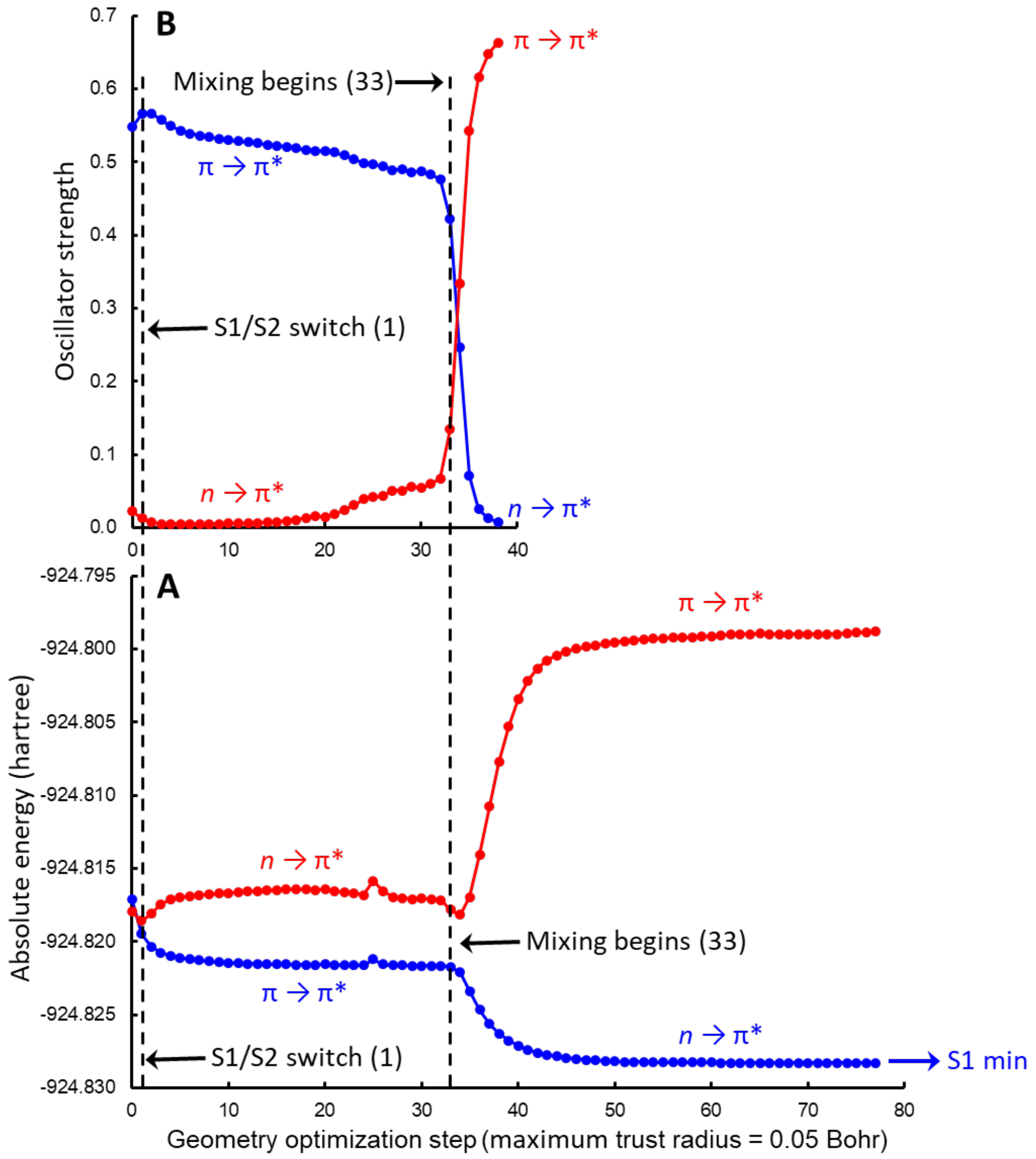

Figure S18: Absolute energies (A) and oscillator strengths (B) of R=$SiMe_2$ excited states S1 and S2 during geometry optimization of the $\pi \rightarrow \pi^*$ singlet excited state which starts out as S2 (at S0 minimum geometry) but on the first geometry step becomes S1. Subsequent geometry optimization of this S1 $\pi \rightarrow \pi^*$ state led to it transforming to $n \rightarrow \pi^*$ character beginning at step 33, and thus quickly acquiring an essentially zero oscillator strength. As such, emission data are reported for geometry step 32. Note that the geometry optimization had a small maximum step size of 0.05 Bohr.

Section S8: NMR Spectra

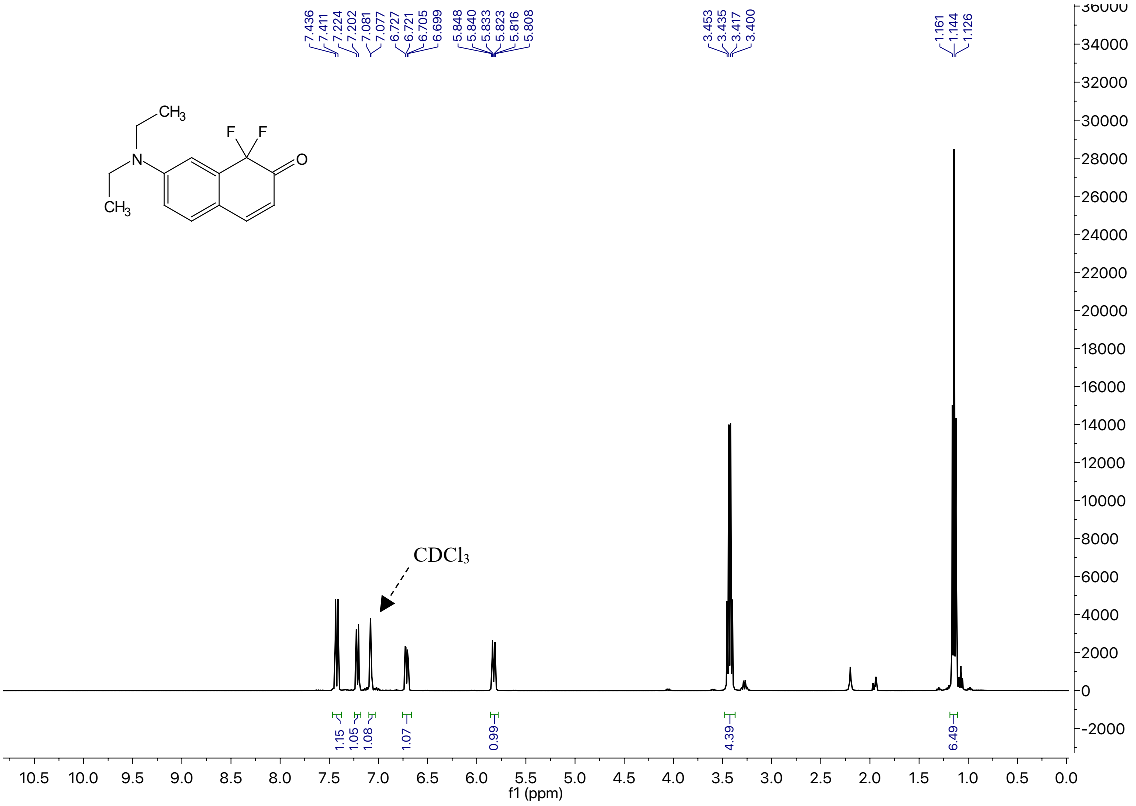

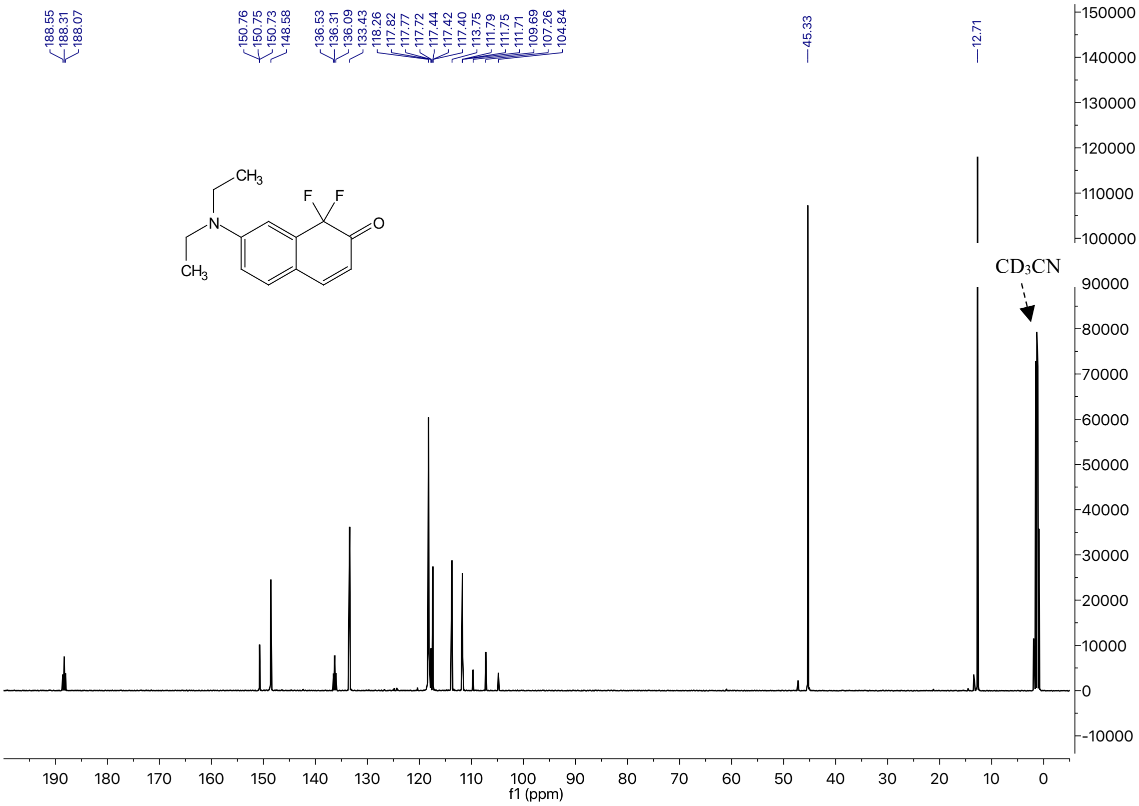

^{1}H (400 MHz) and ^{13}C (100 MHz) NMR spectra of **FC2**.

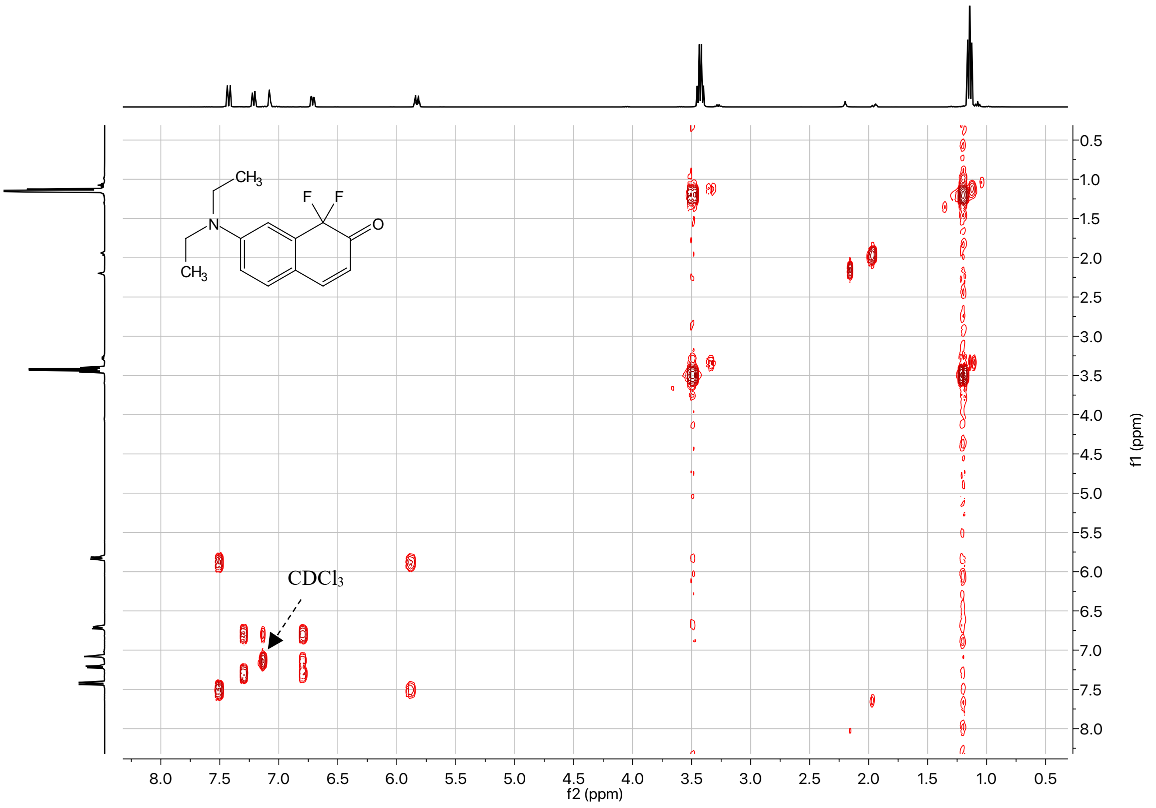

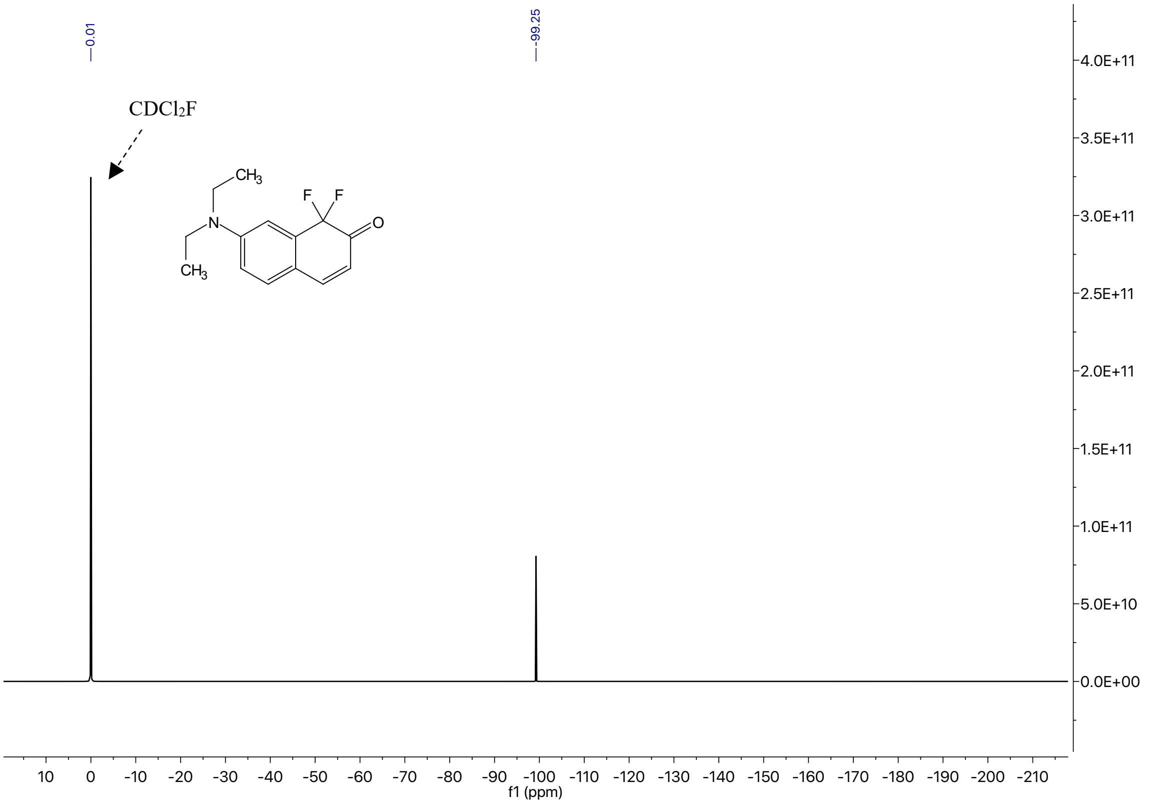

gCOSY (400 MHz) and ^{19}F (376 MHz) NMR spectra of **FC2**.

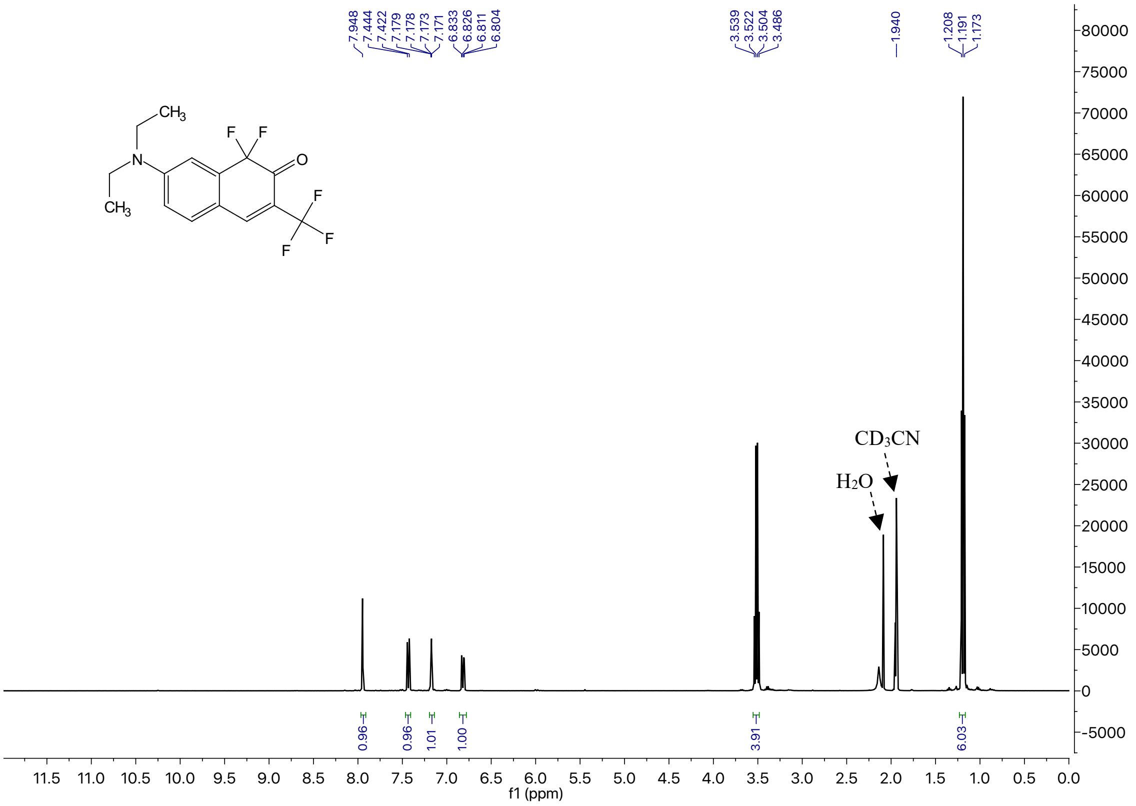

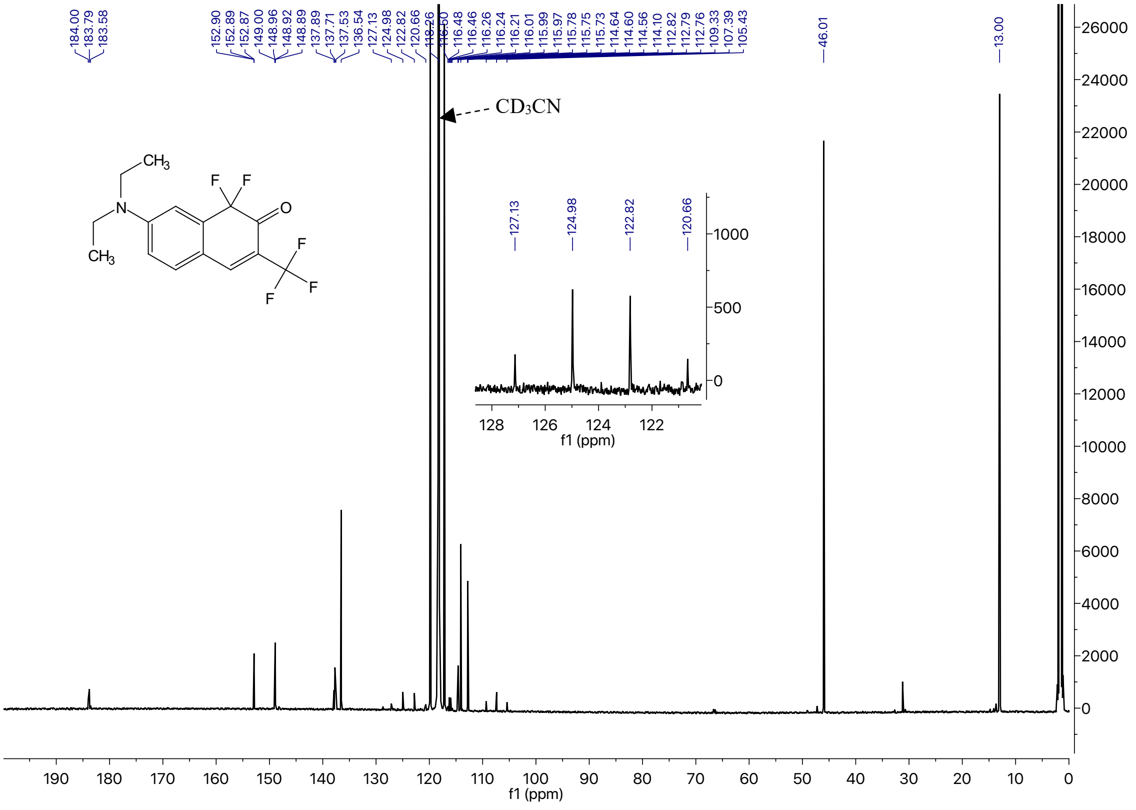

^{1}H (400 MHz) and ^{13}C (125 MHz) NMR spectra of **FC3**.

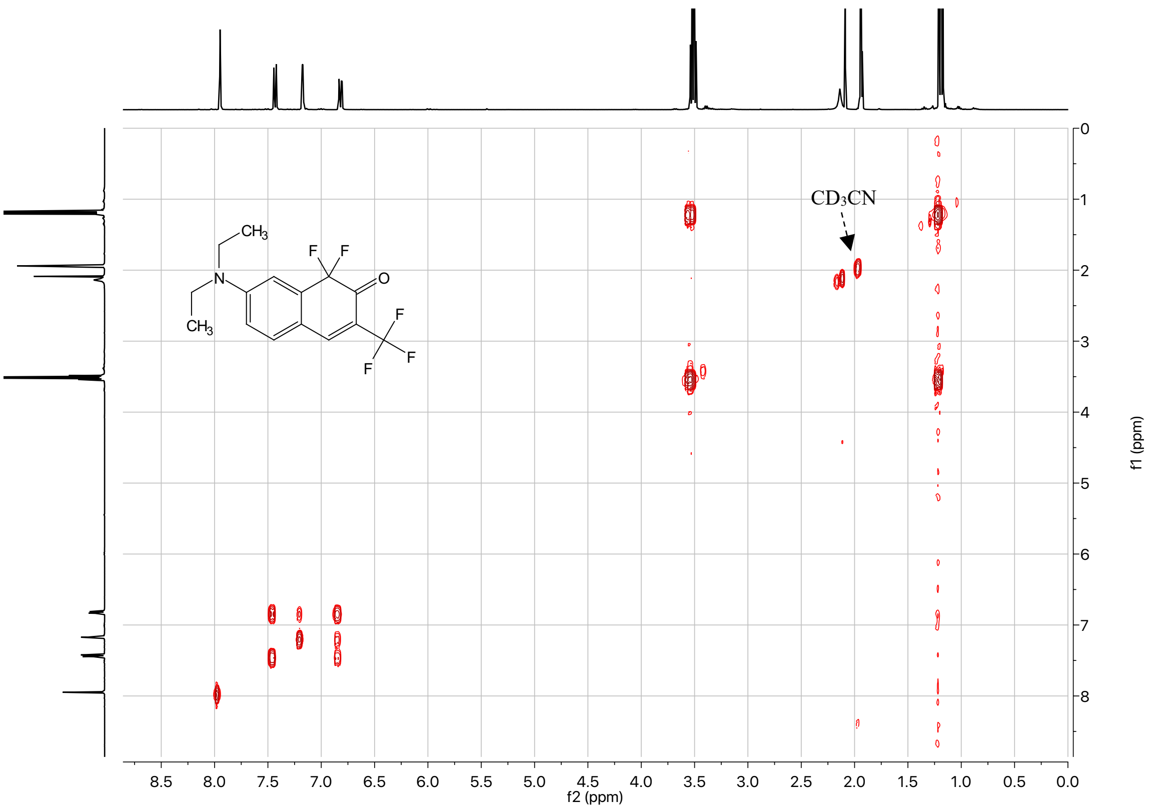

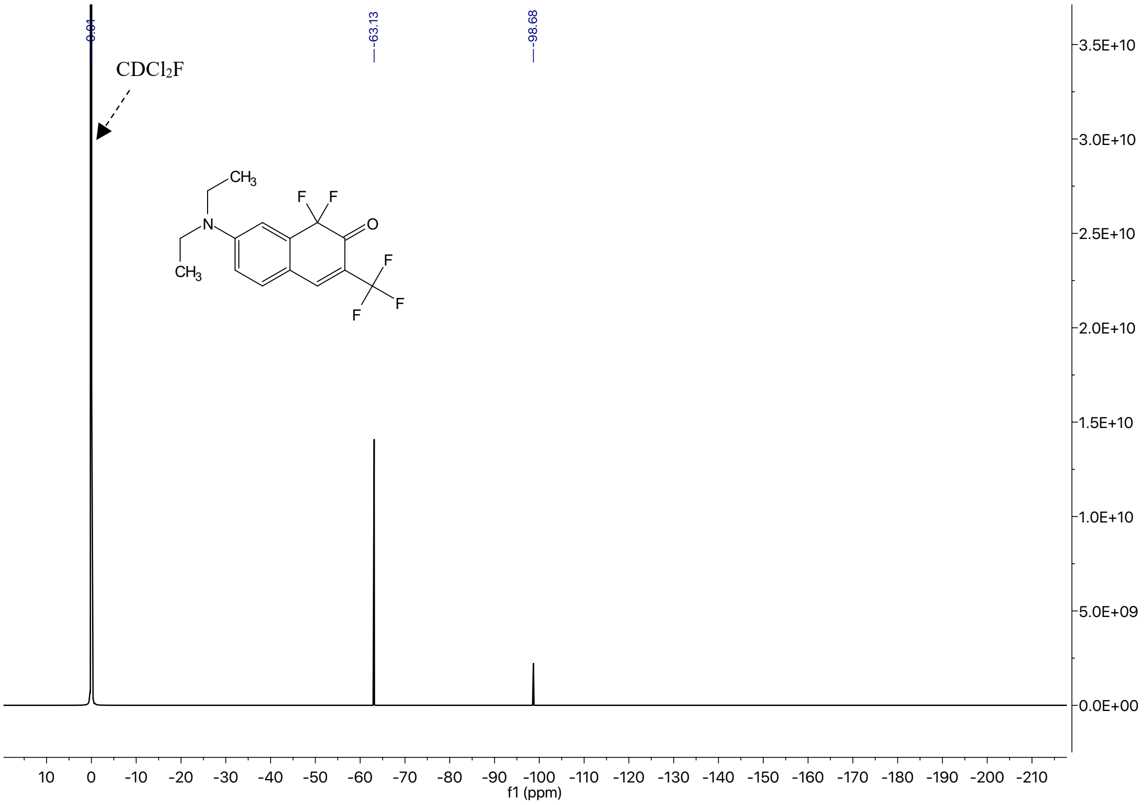

gCOSY (400 MHz) and ^{19}F (376 MHz) NMR spectra of **FC3**.

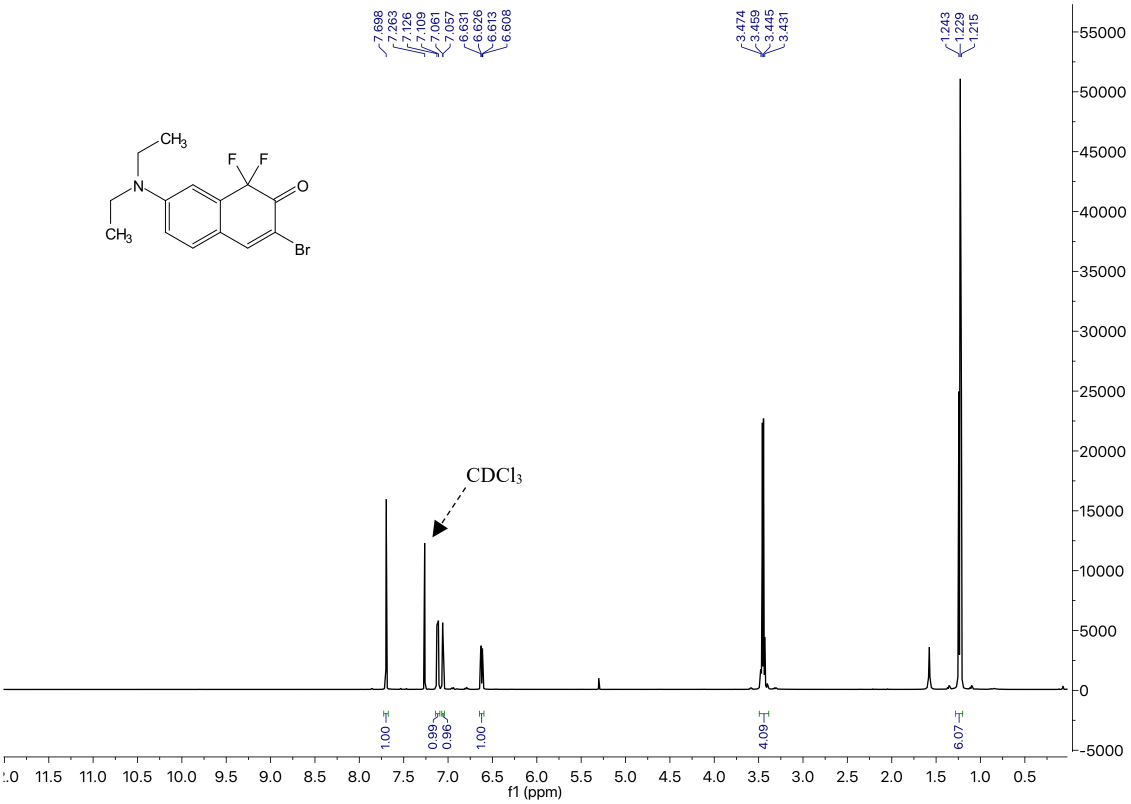

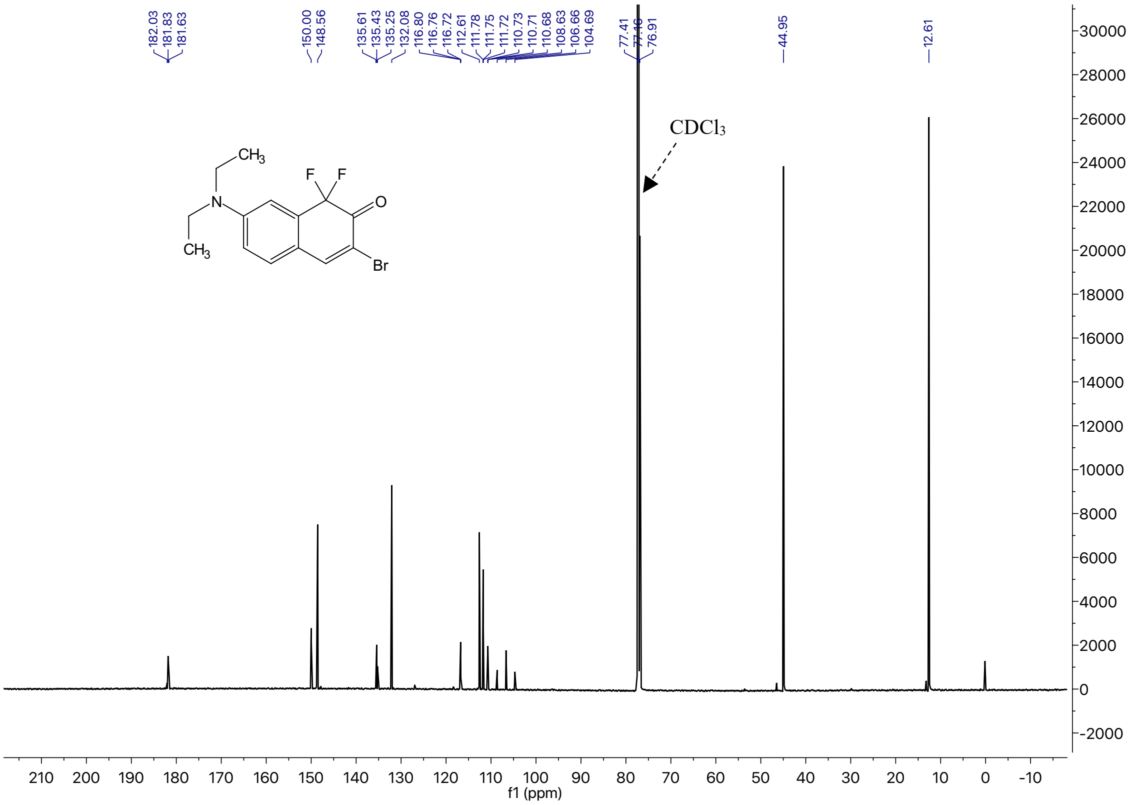

^{1}H (500 MHz) and ^{13}C (125 MHz) NMR spectra of **FC4**.

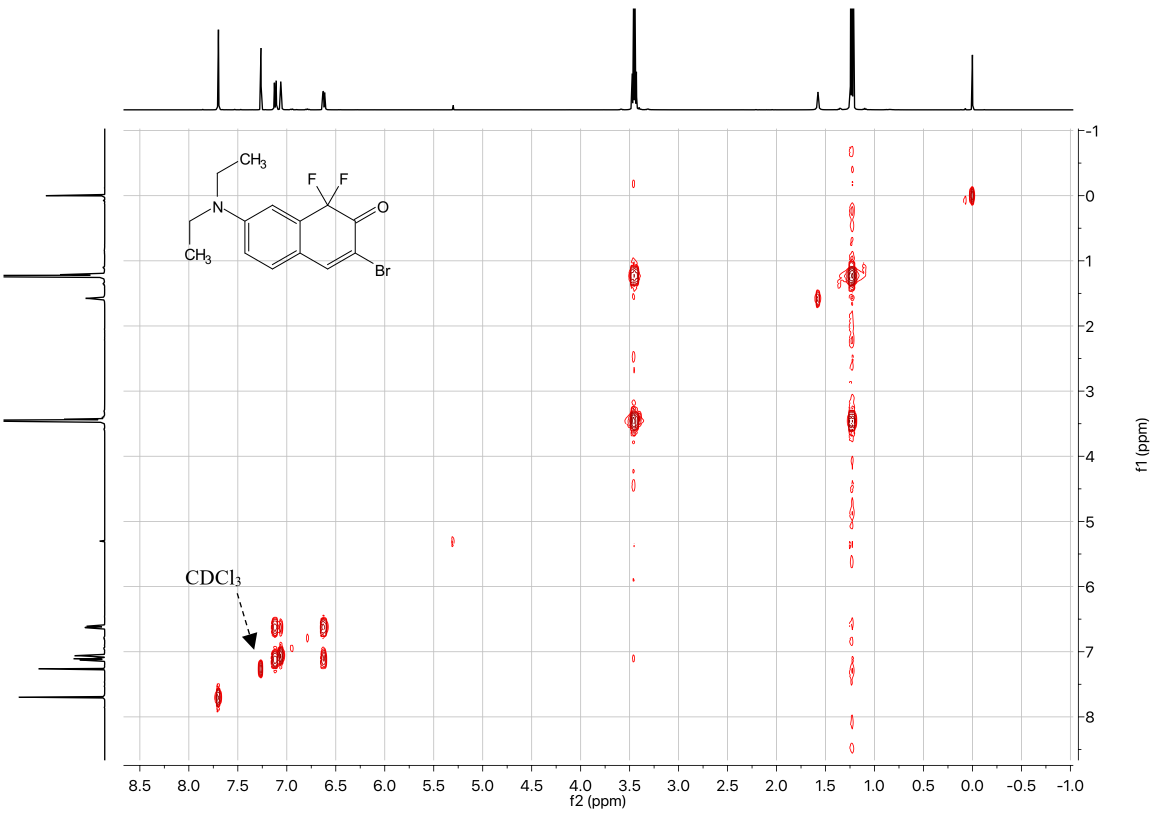

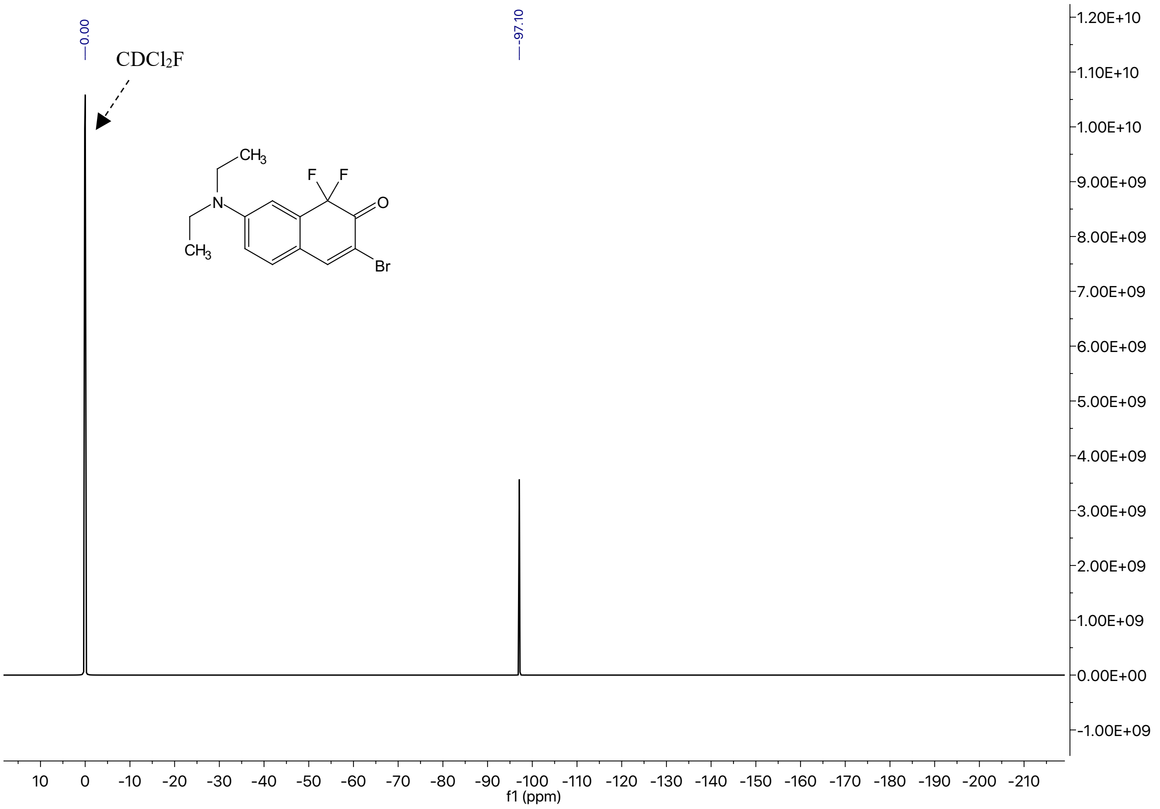

gCOSY (500 MHz) and ^{19}F (376 MHz) NMR spectra of **FC4**.

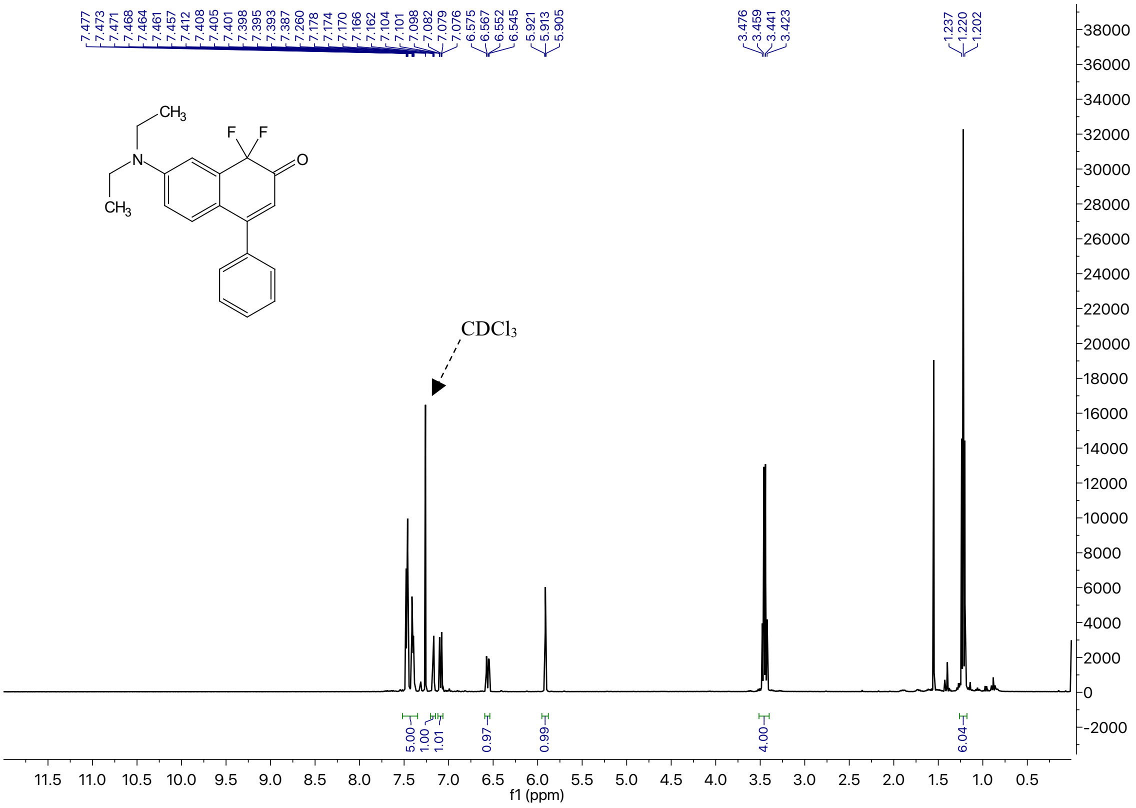

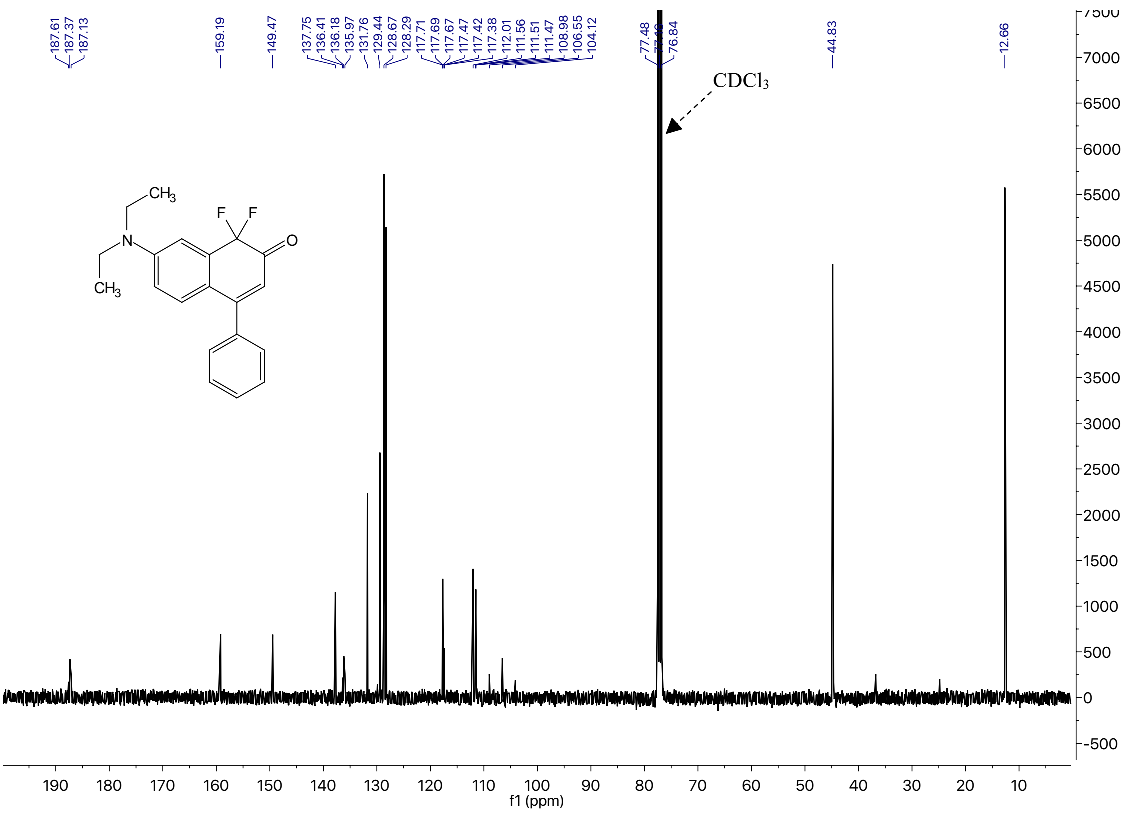

^{1}H (400 MHz) and ^{13}C (125 MHz) NMR spectra of **FC5**.

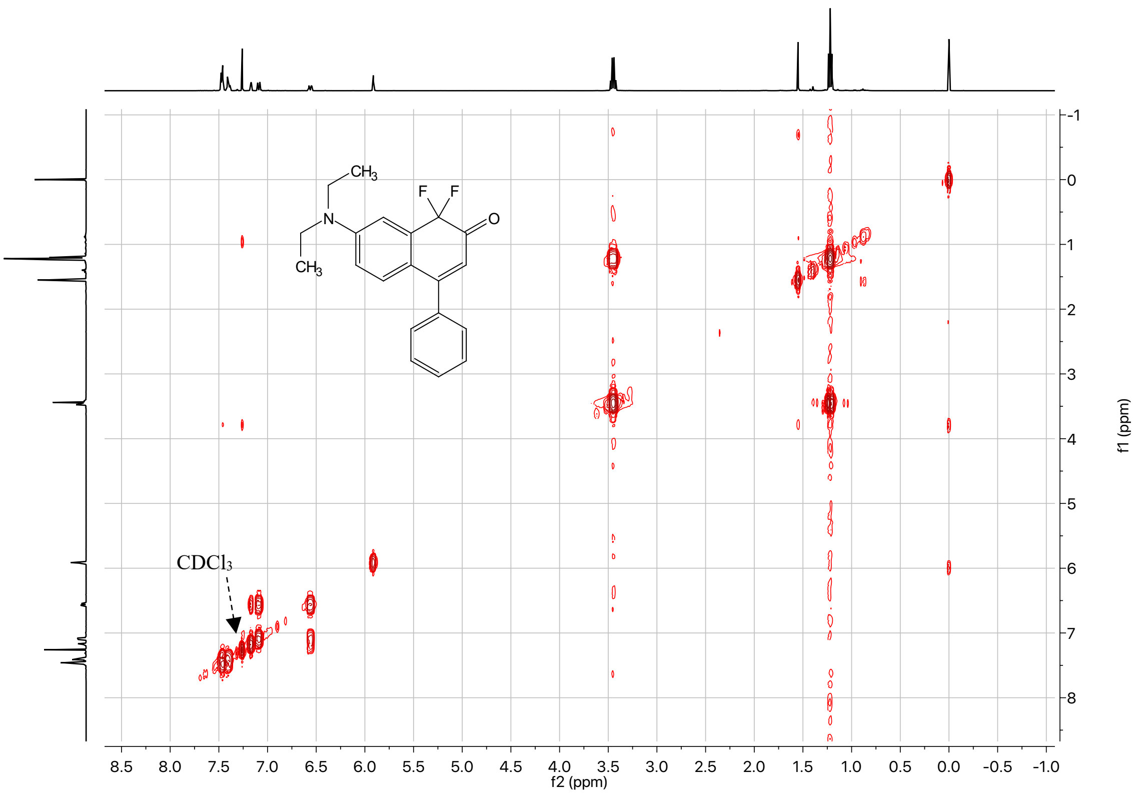

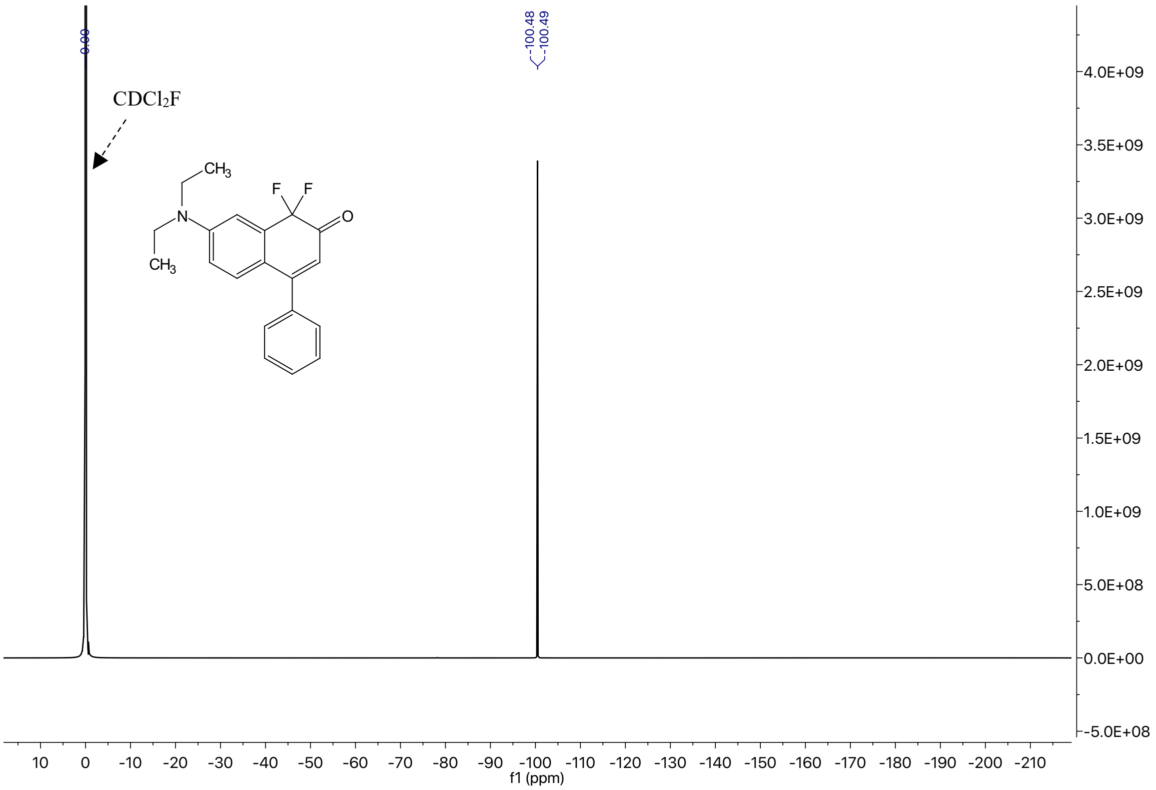

gCOSY (500 MHz) and ^{19}F (376 MHz) NMR spectra of **FC5**.

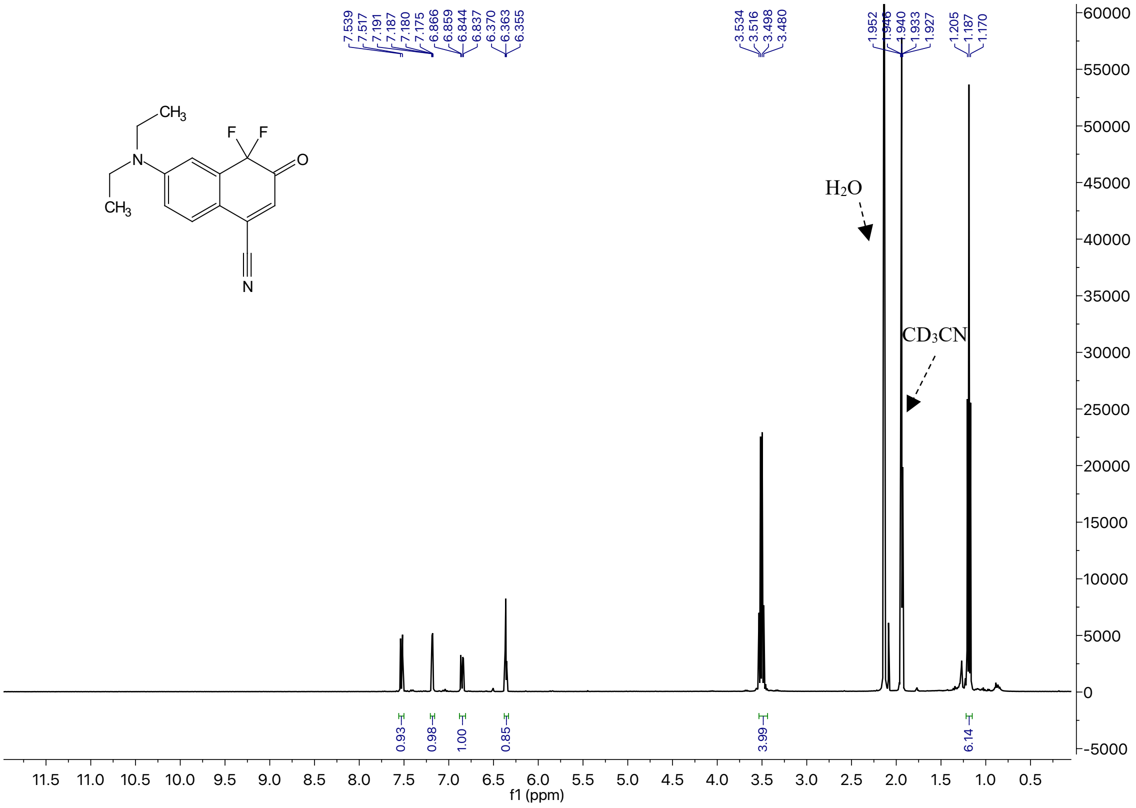

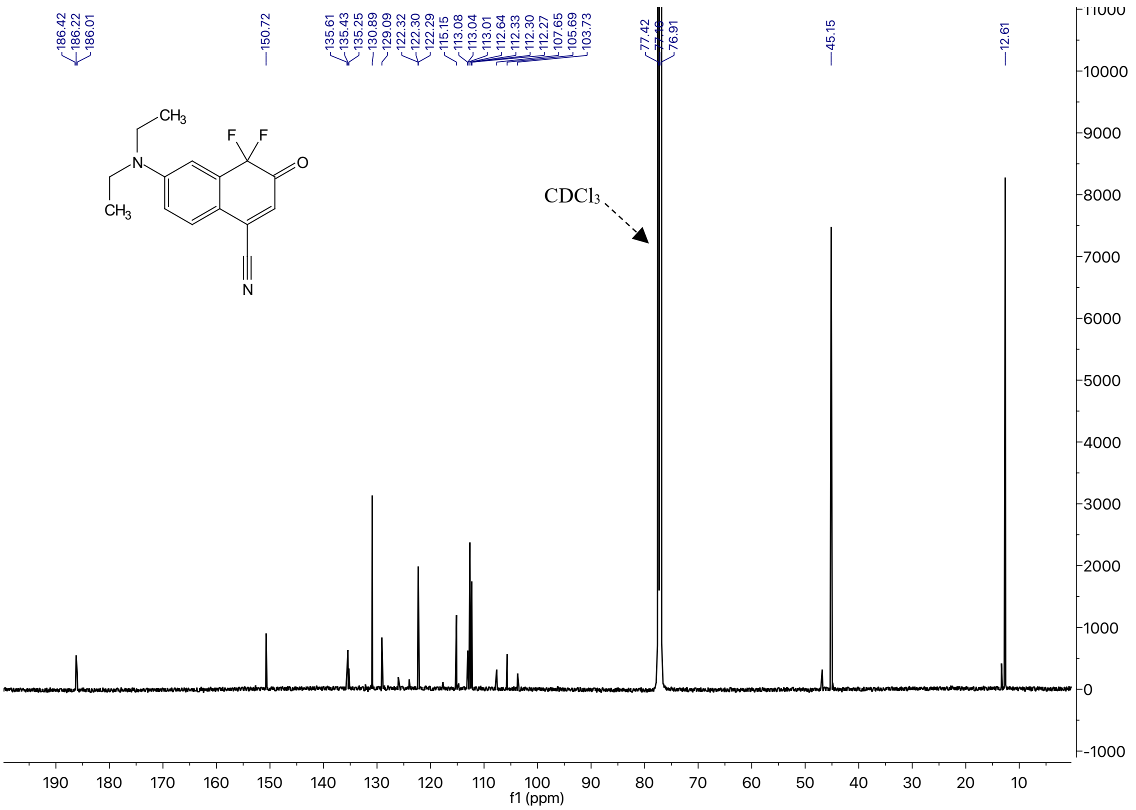

^{1}H (400 MHz) and ^{13}C (125 MHz) NMR spectra of **FC6**.

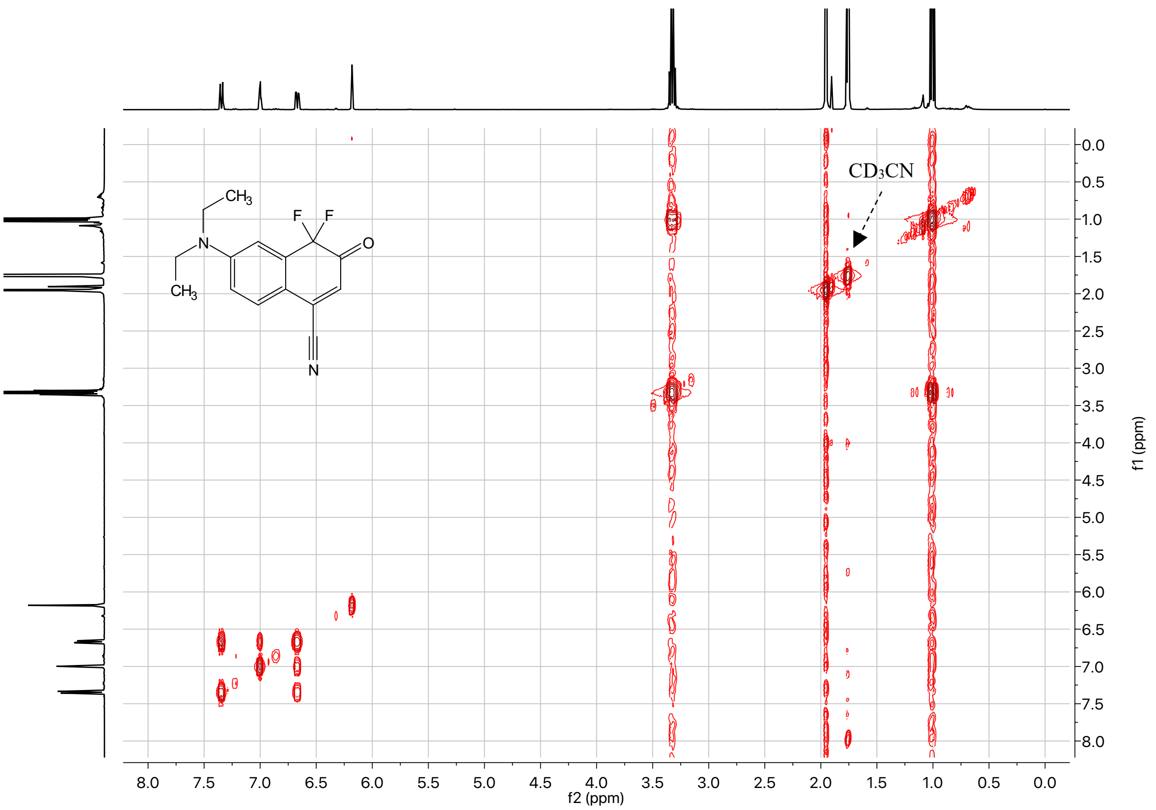

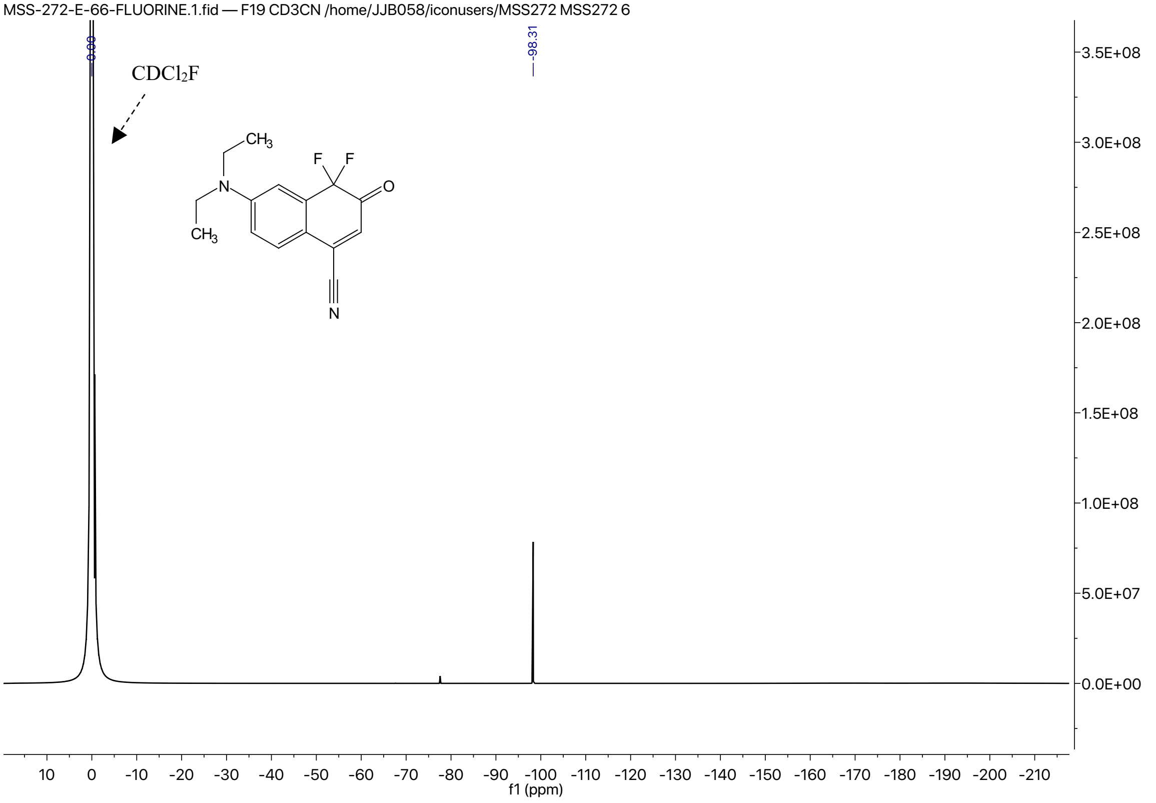

gCOSY (400 MHz) and ^{19}F (376 MHz) NMR spectra of **FC6**.

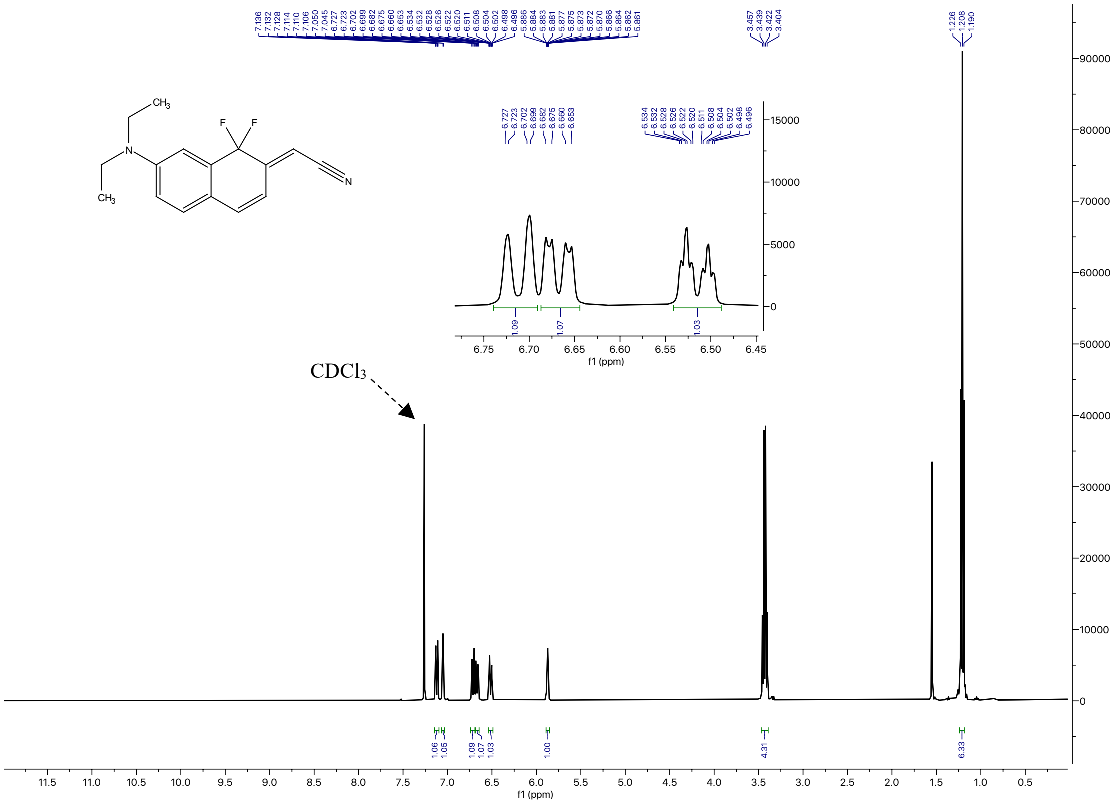

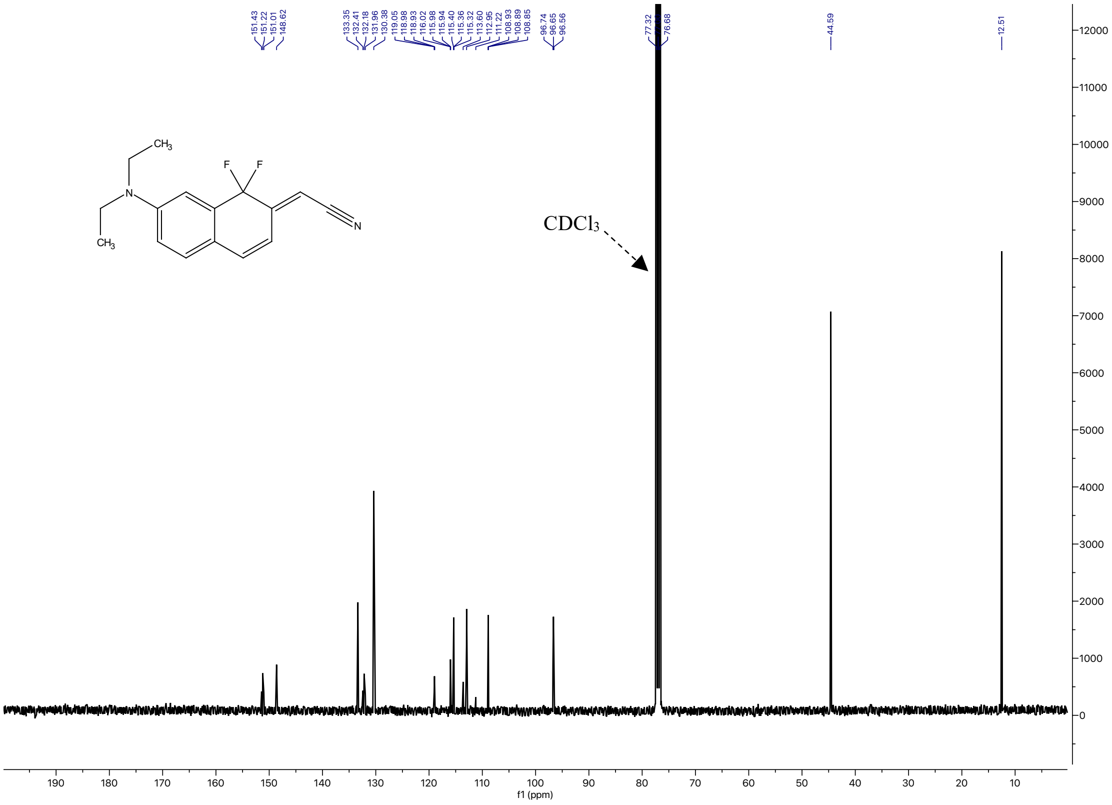

^{1}H (400 MHz) and ^{13}C (100 MHz) NMR spectra of **FC7**.

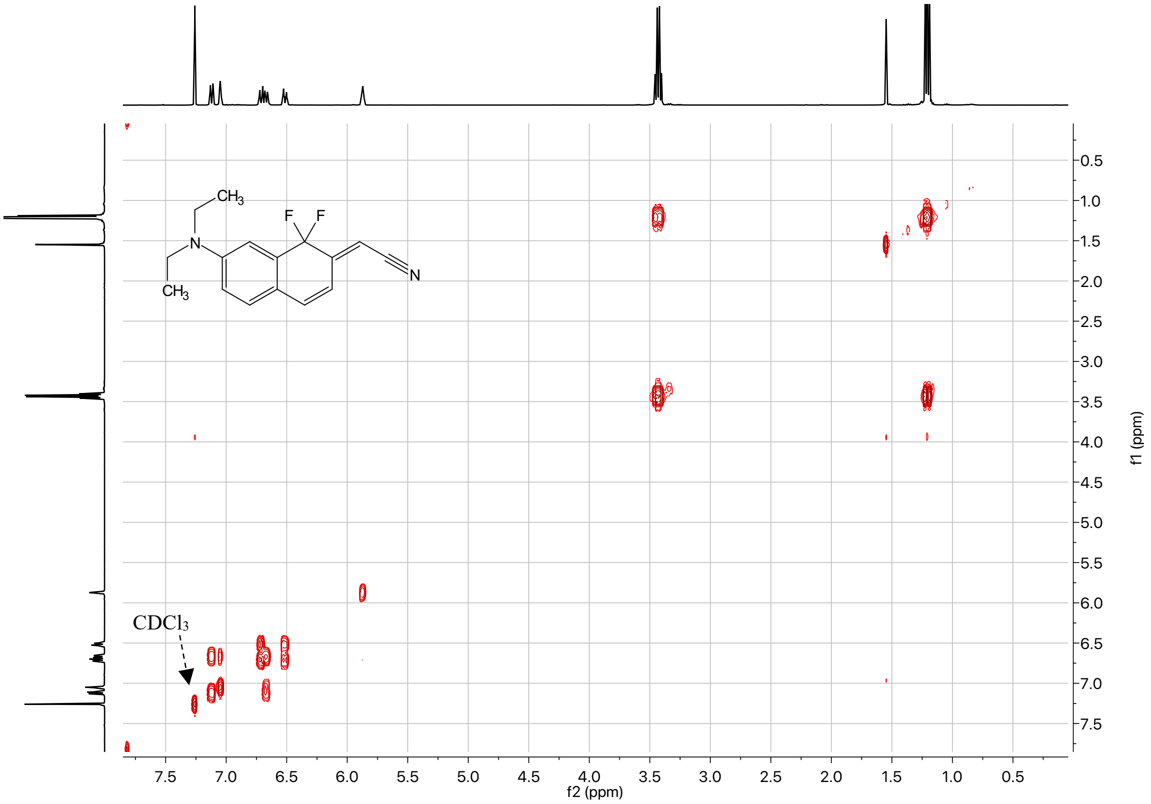

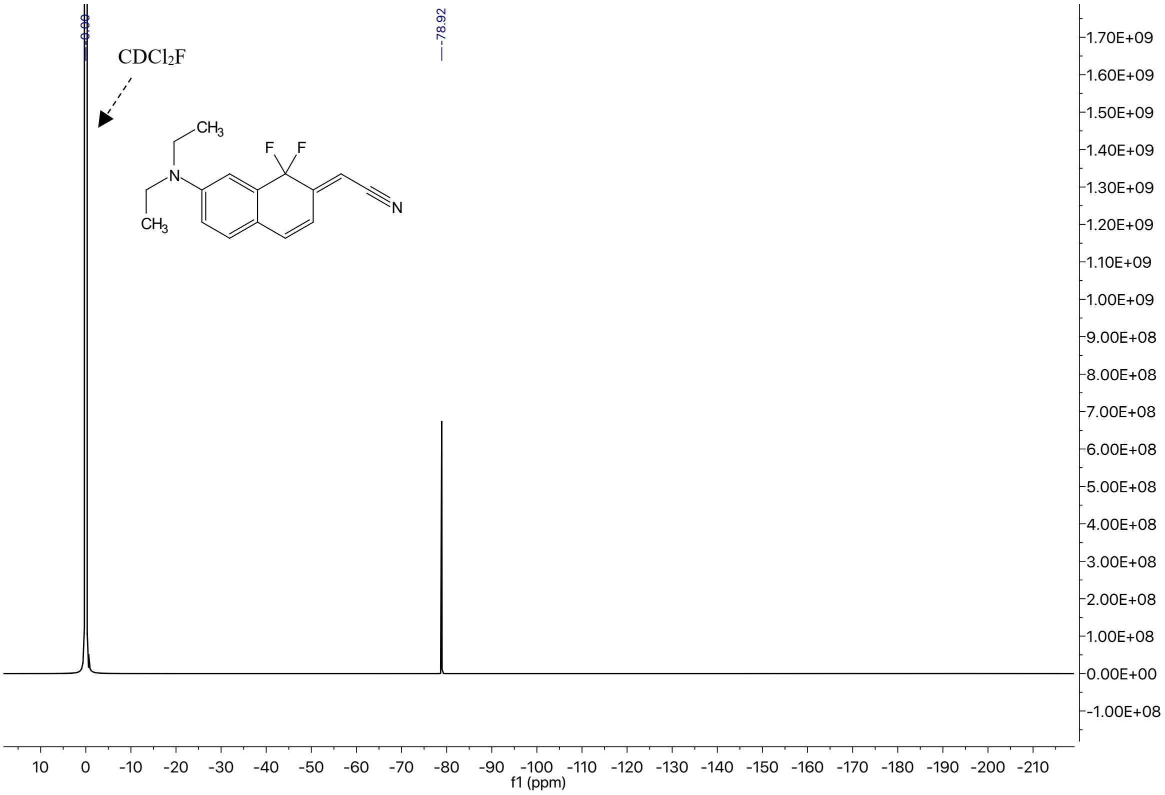

gCOSY (400 MHz) and ^{19}F (376 MHz) NMR spectra of **FC7**.

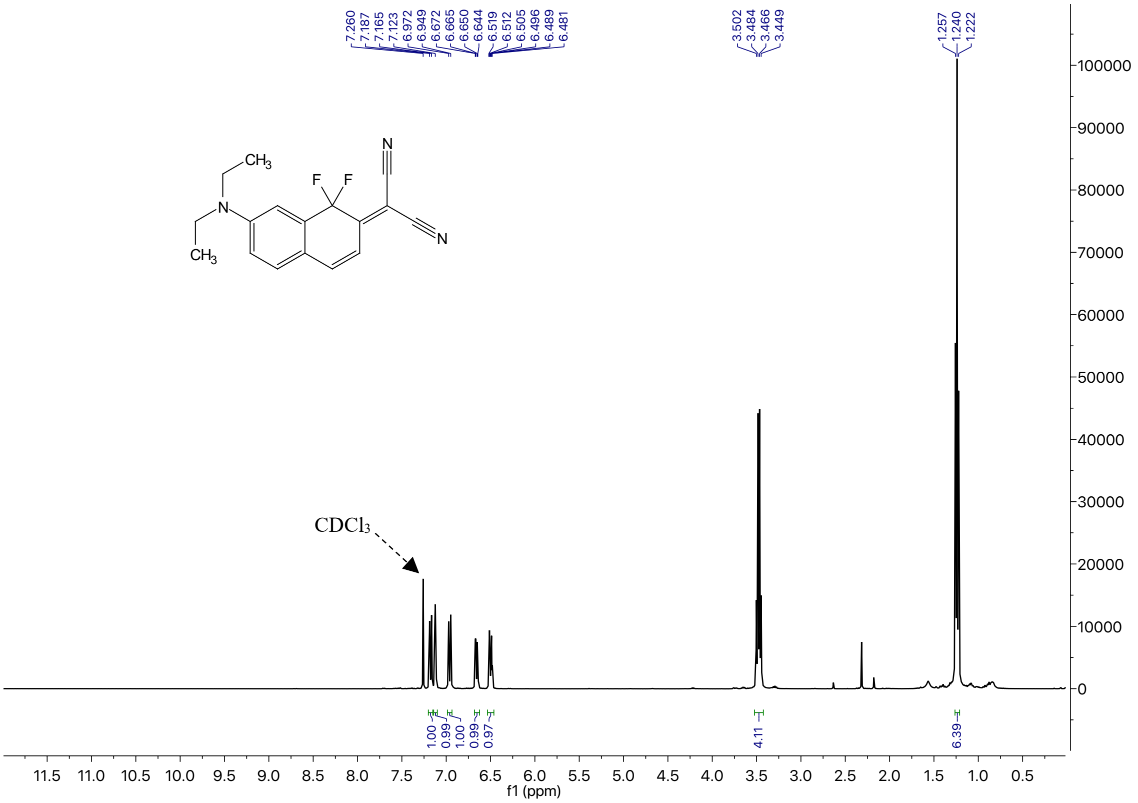

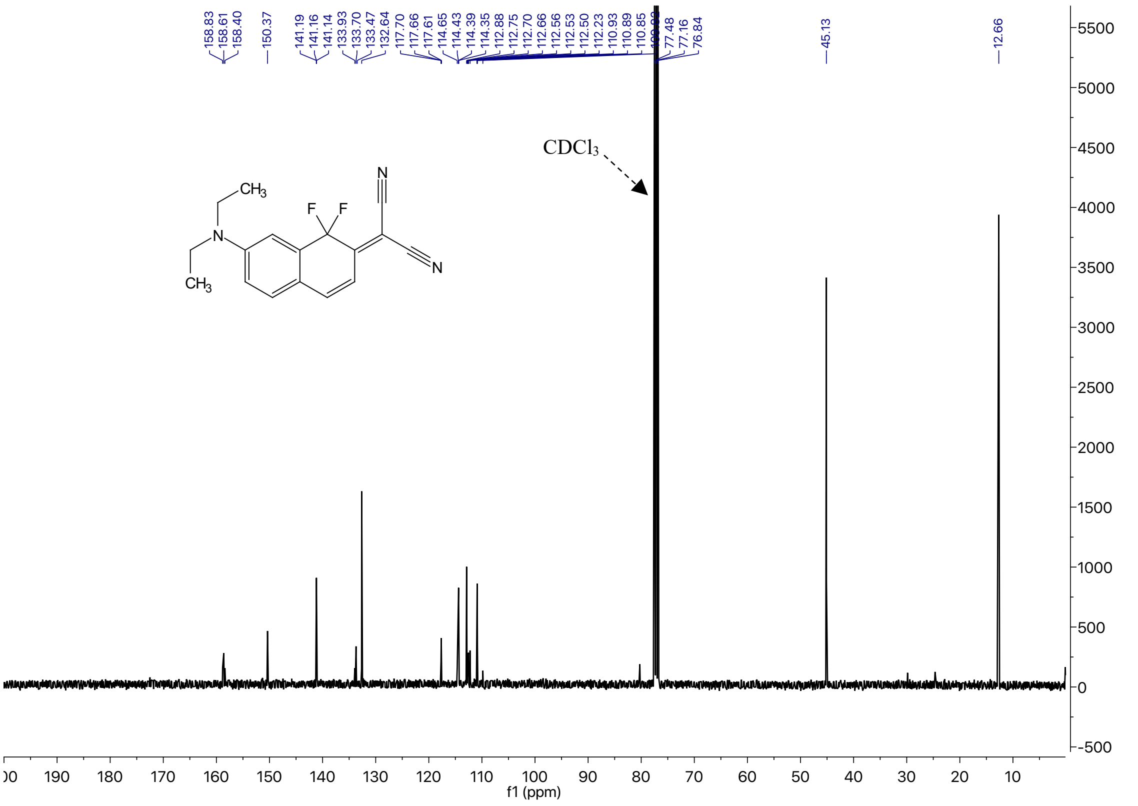

^{1}H (400 MHz) and ^{13}C (100 MHz) NMR spectra of **FC8**.

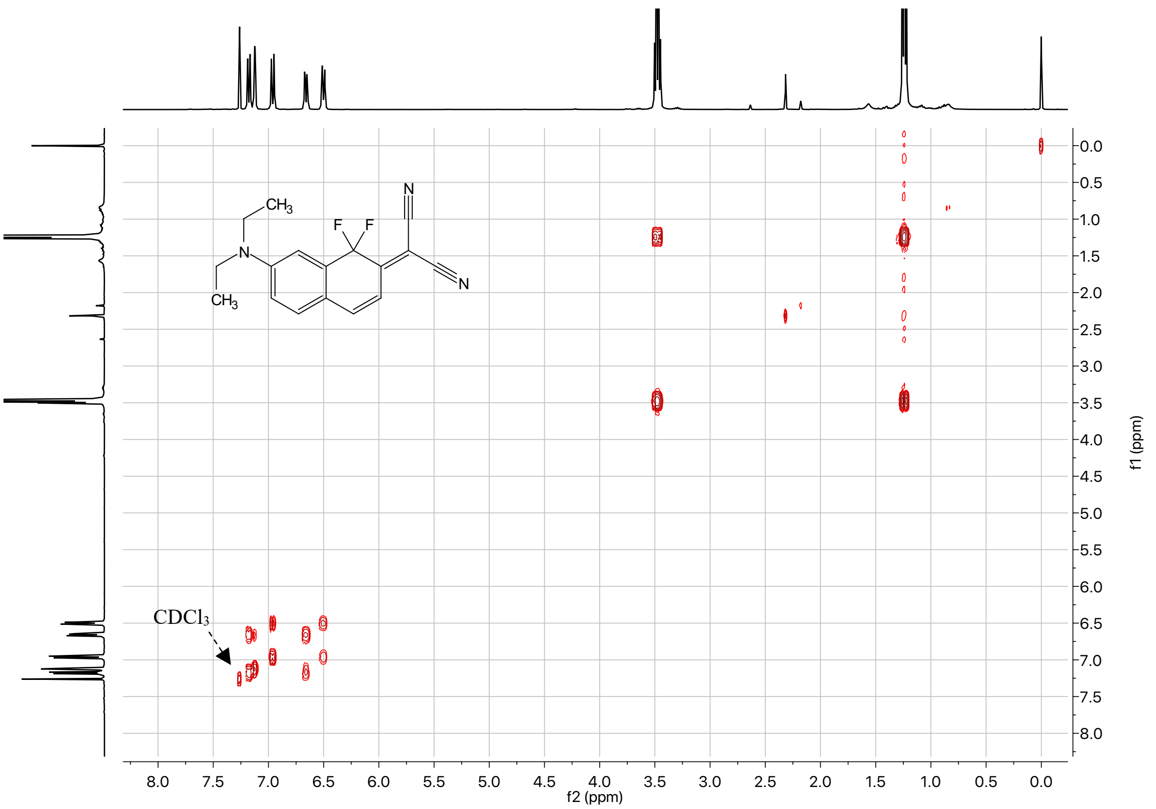

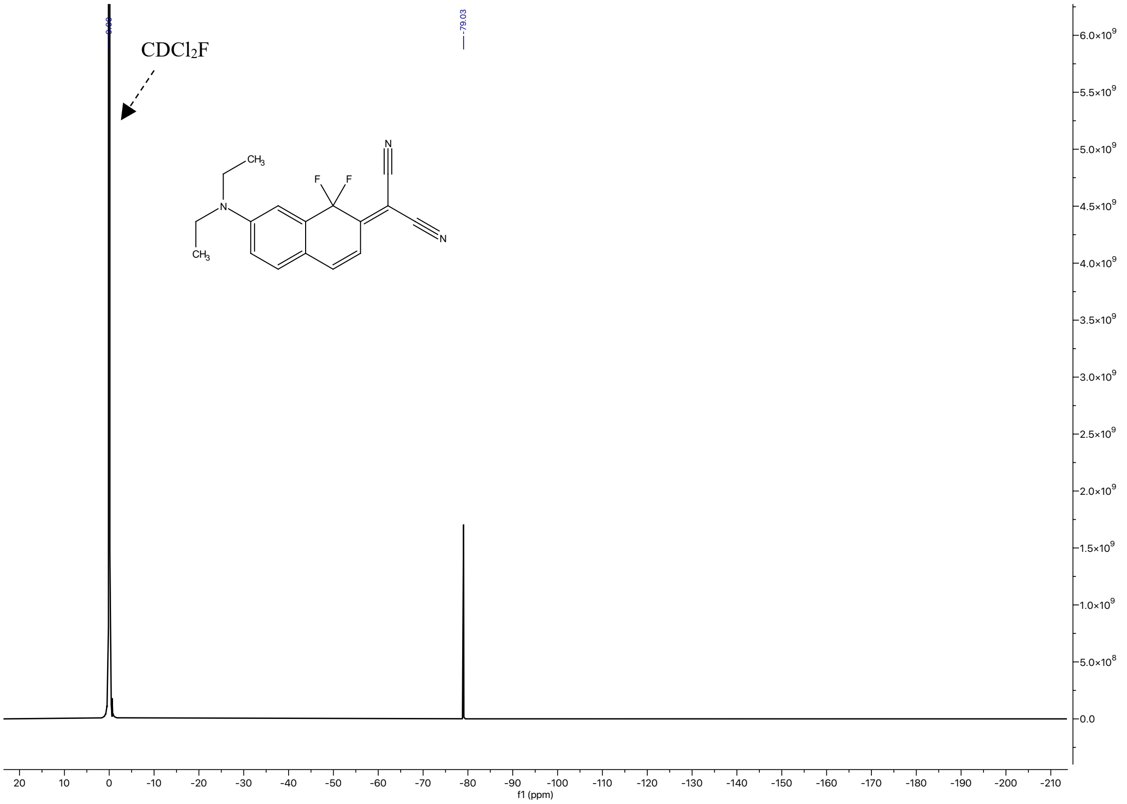

gCOSY (400 MHz) and ^{19}F (376 MHz) NMR spectra of **FC8**.

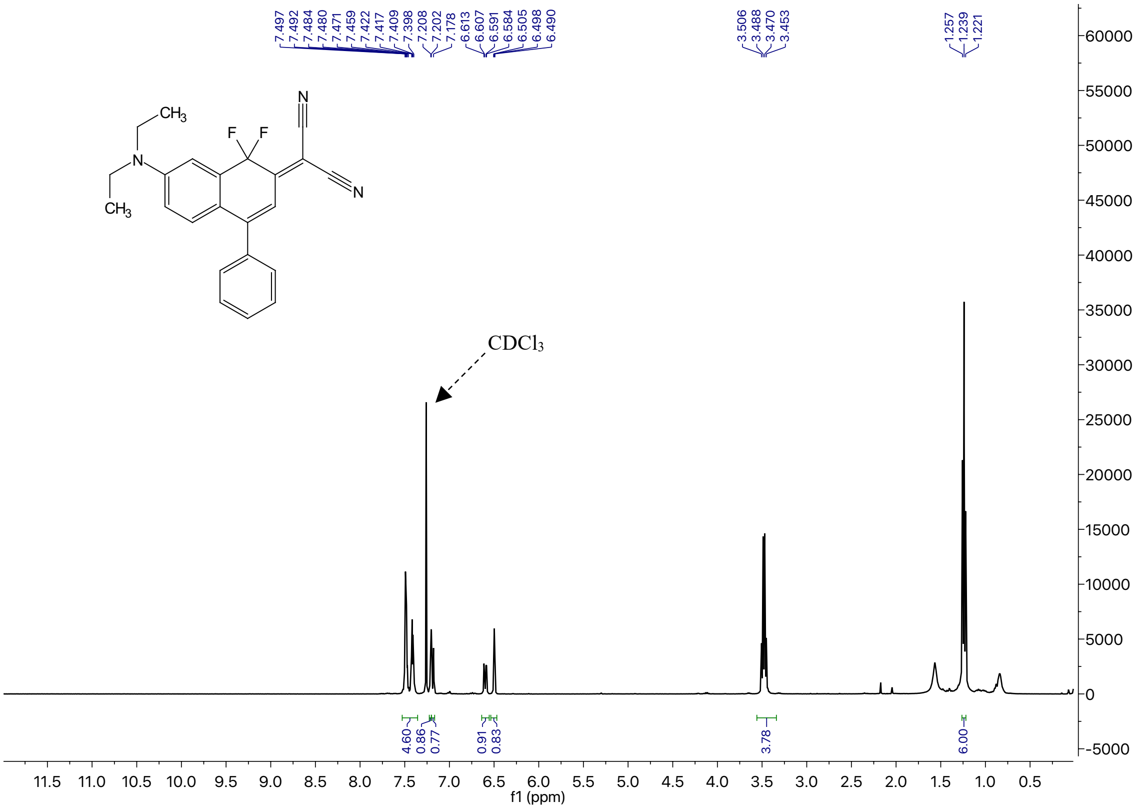

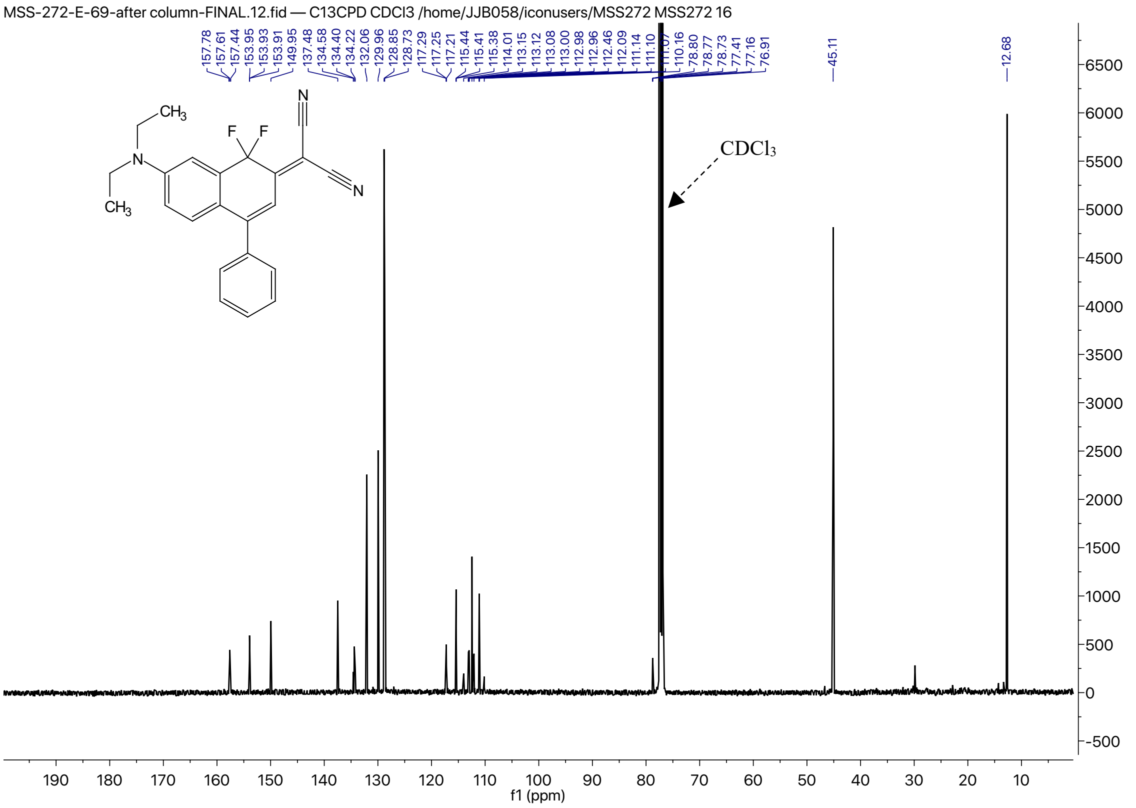

^{1}H (400 MHz) and ^{13}C (125 MHz) NMR spectra of **FC9**.

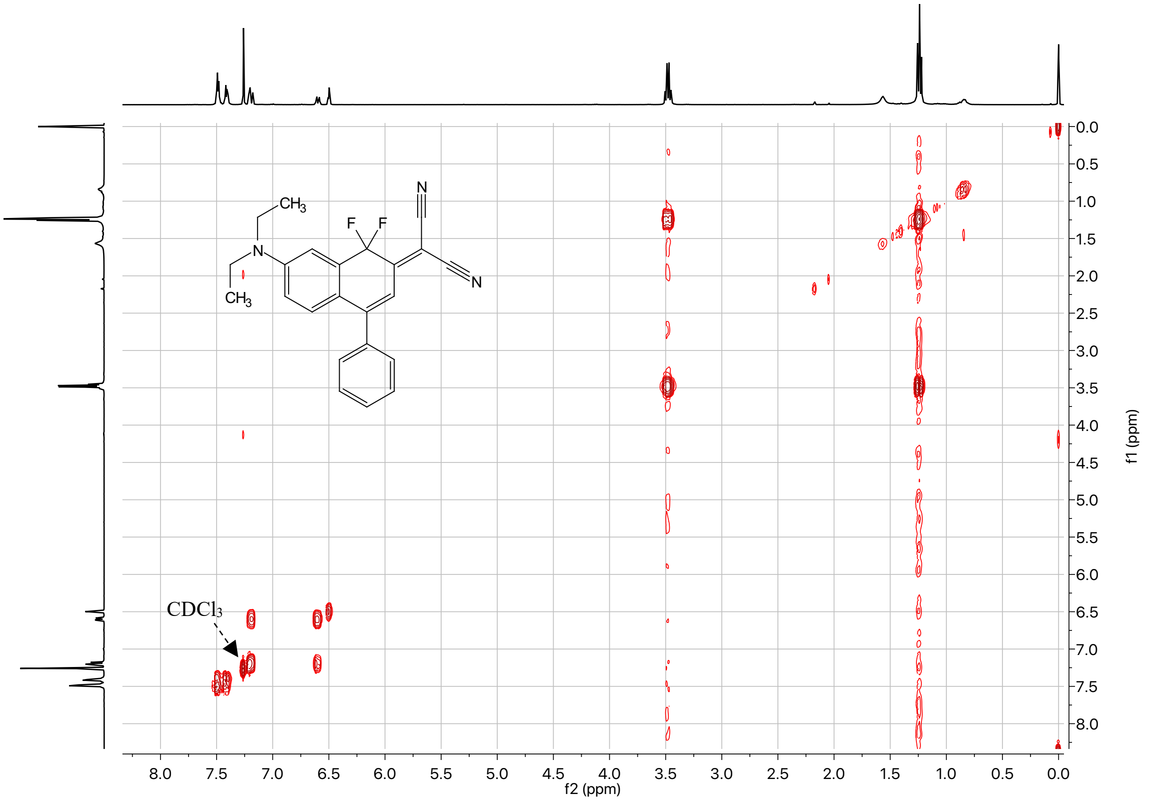

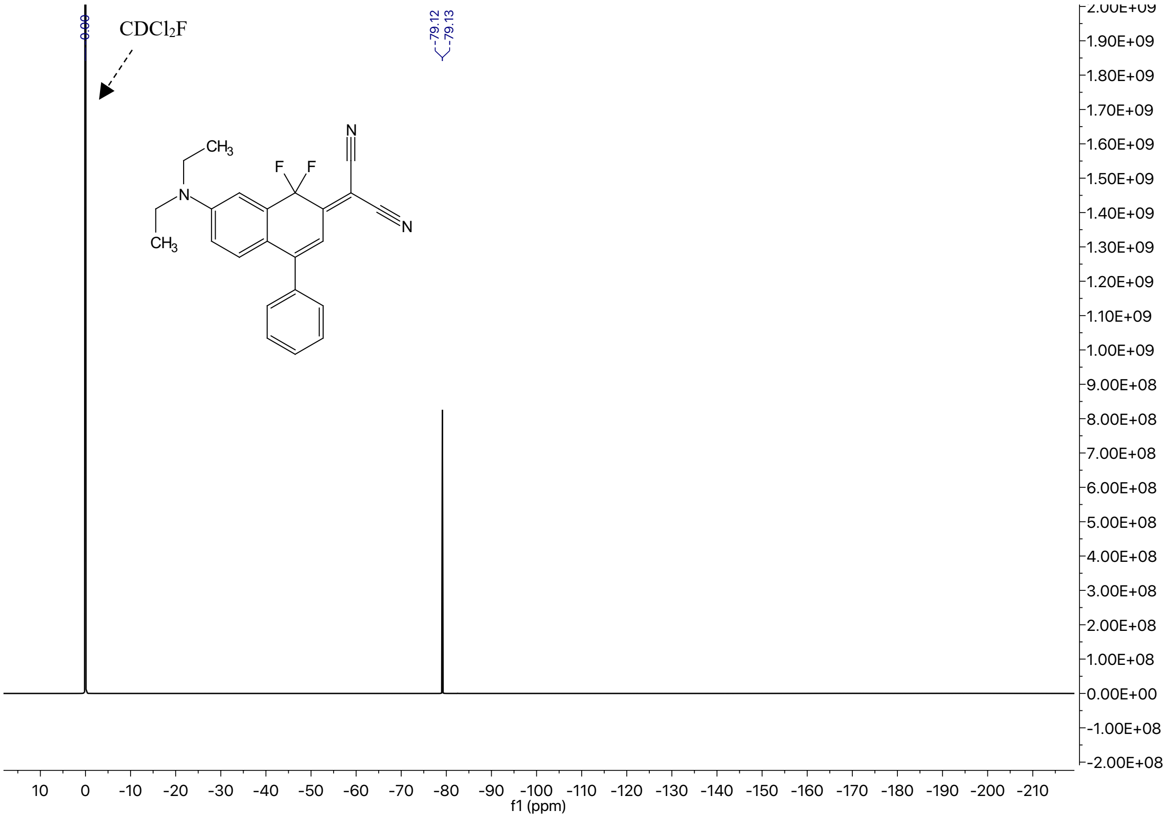

gCOSY (400 MHz) and ^{19}F (376 MHz) NMR spectra of **FC9**.

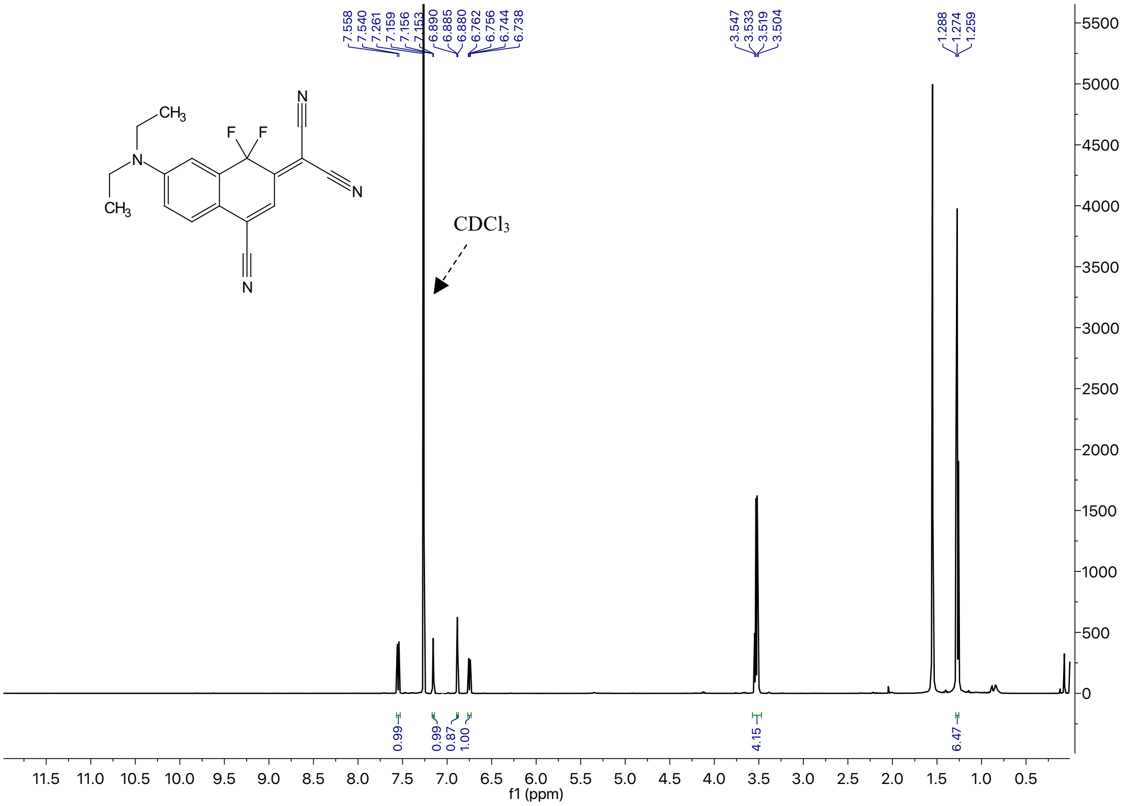

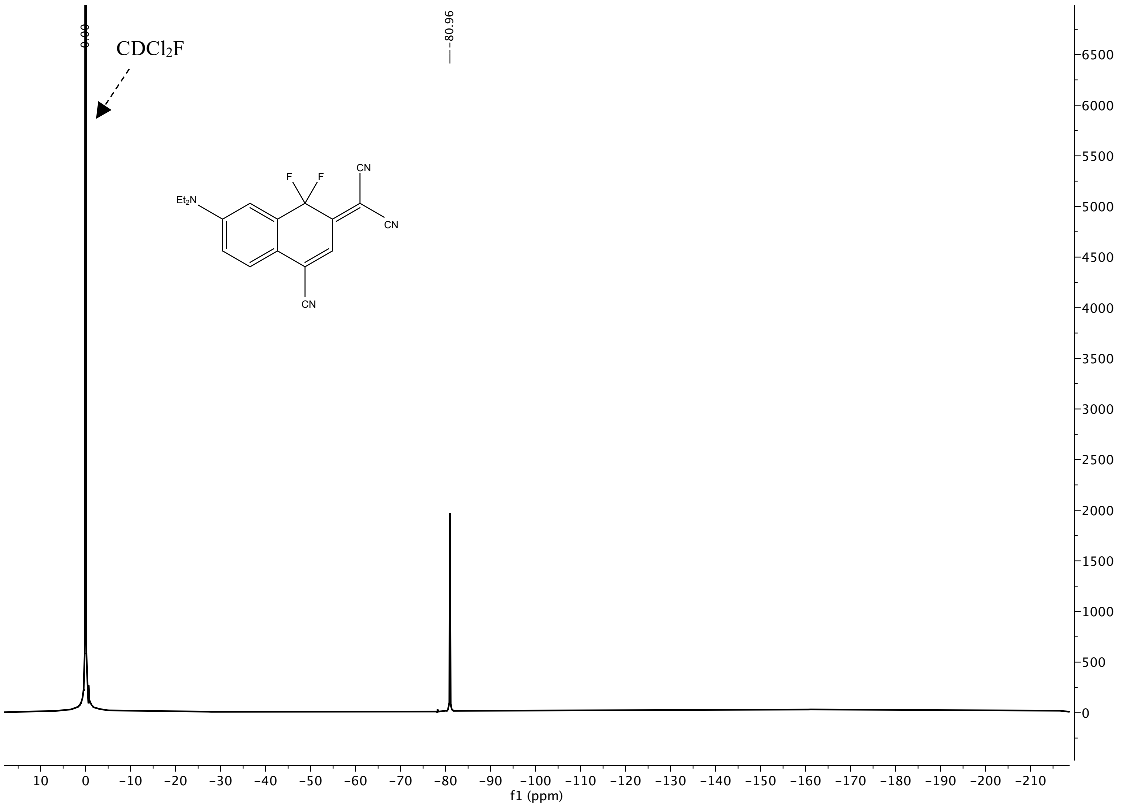

^{1}H (500 MHz) and ^{19}F (376 MHz) NMR spectra of **FC10**.

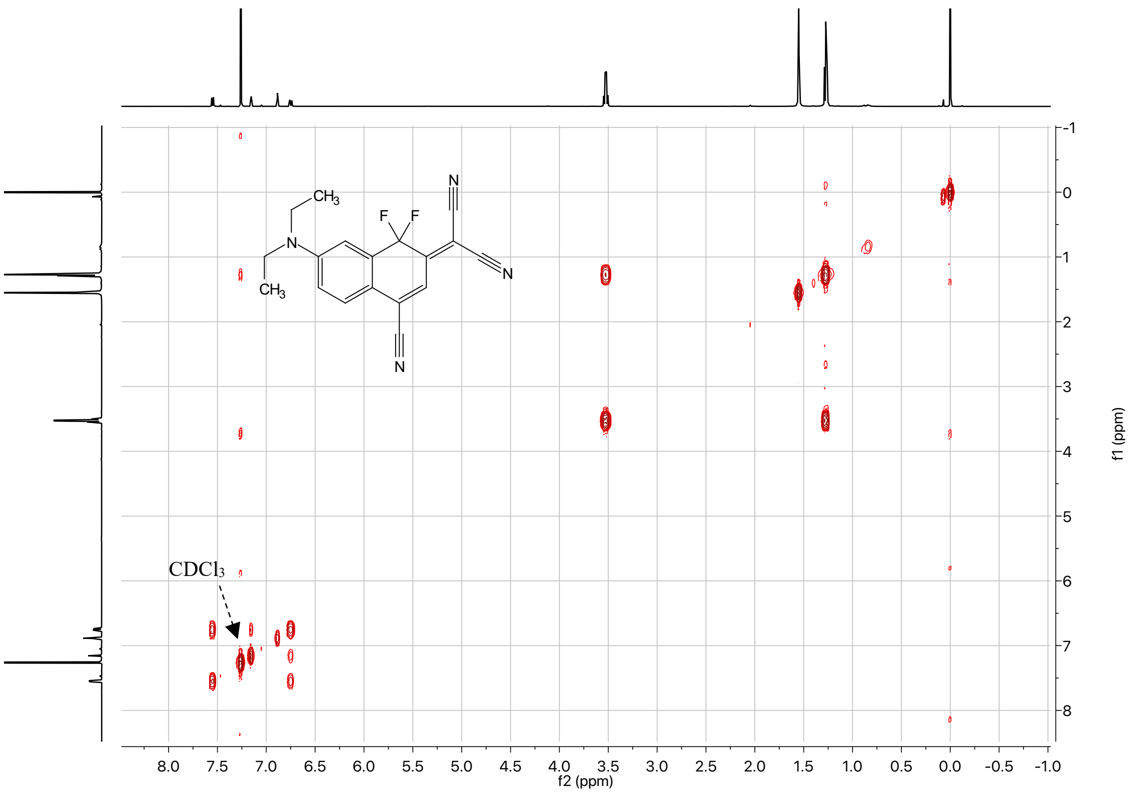

gCOSY (500 MHz) NMR spectra of **FC10**.

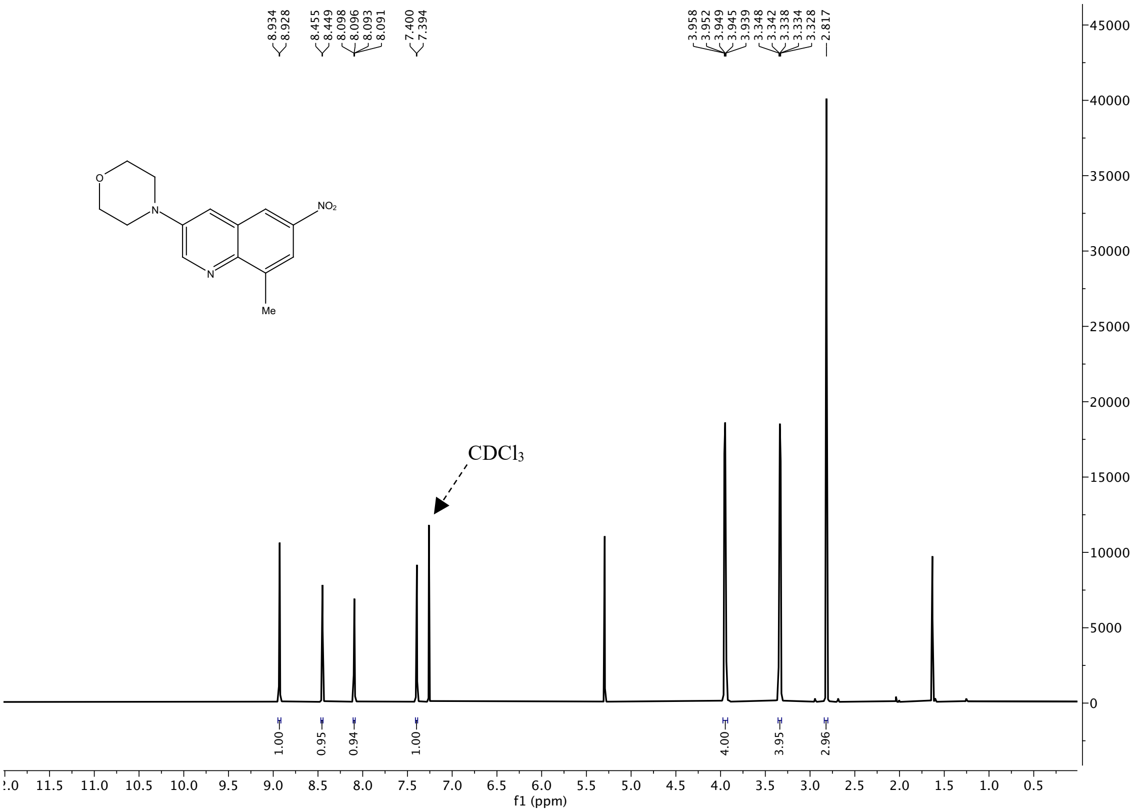

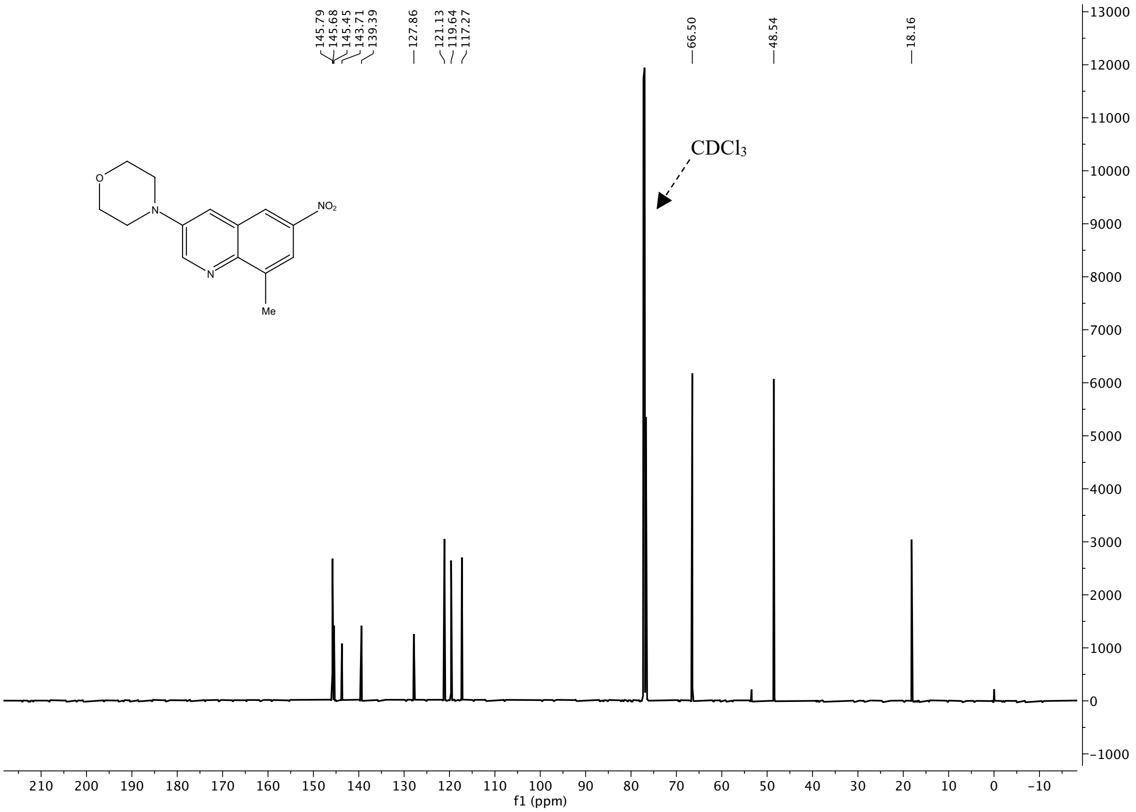

^{1}H (500 MHz) and ^{13}C (125 MHz) NMR spectra of **5**.

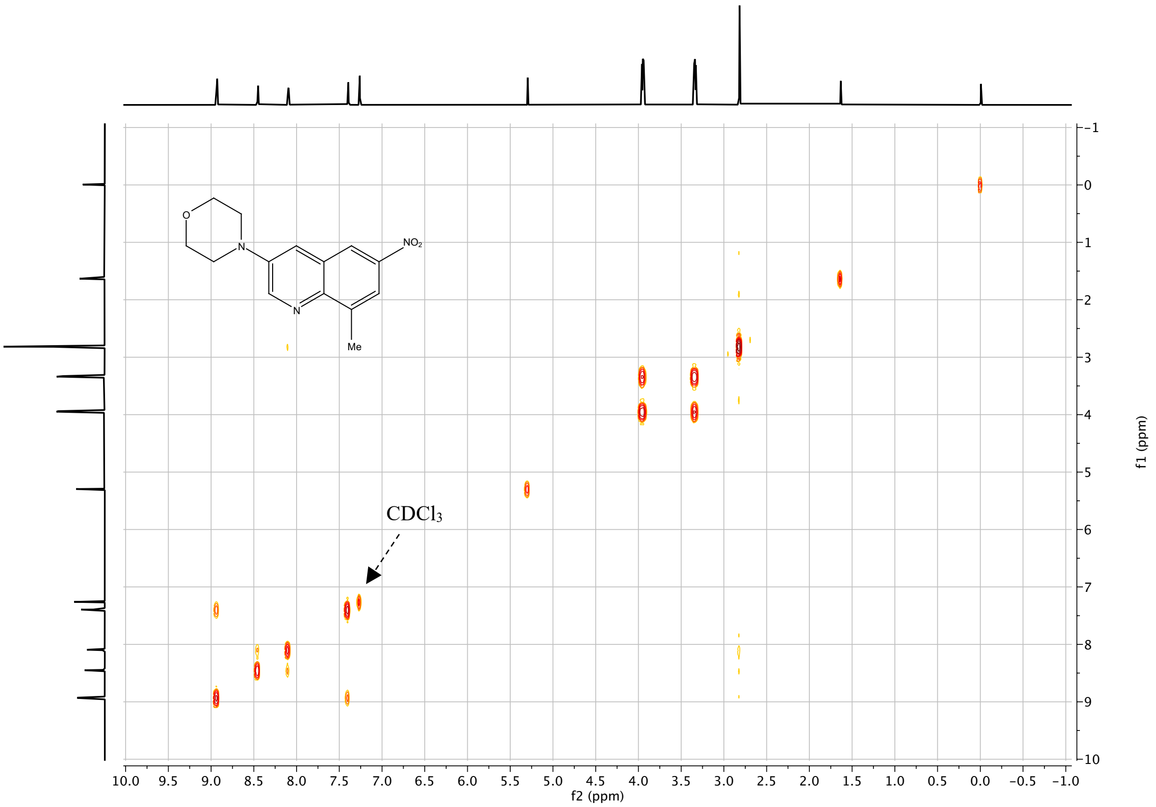

gCOSY (500 MHz) NMR spectra of **5**.

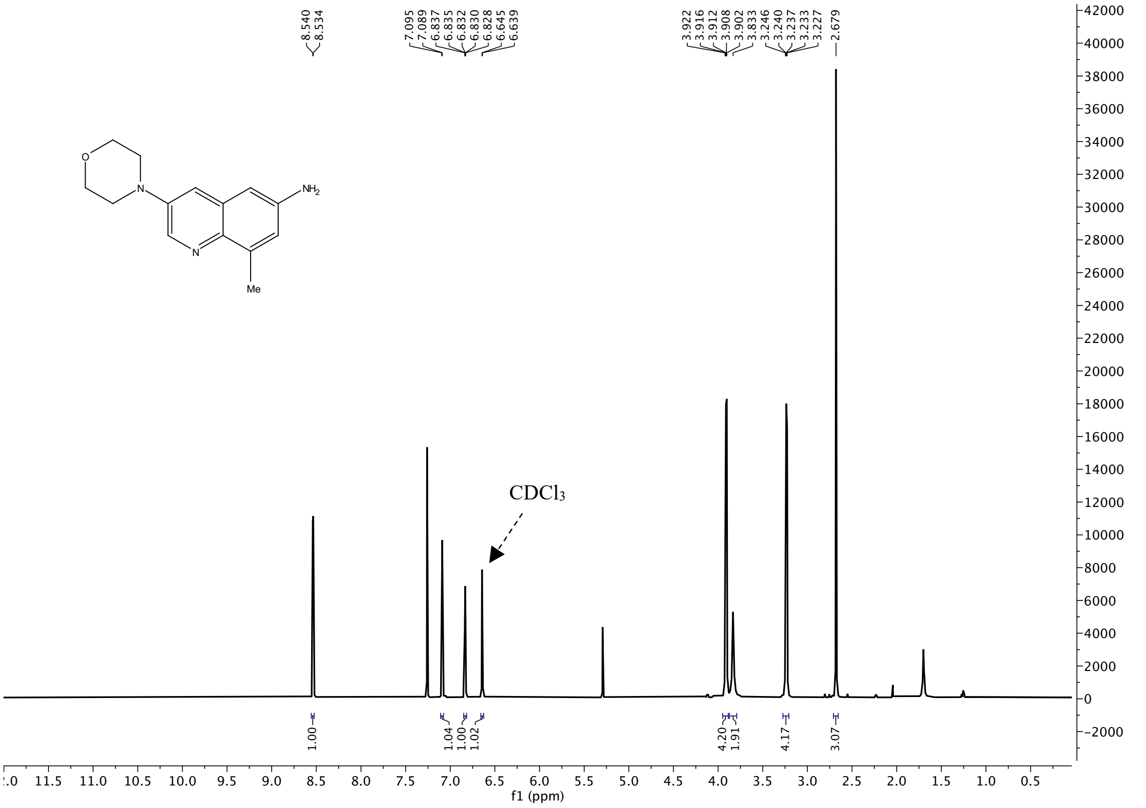

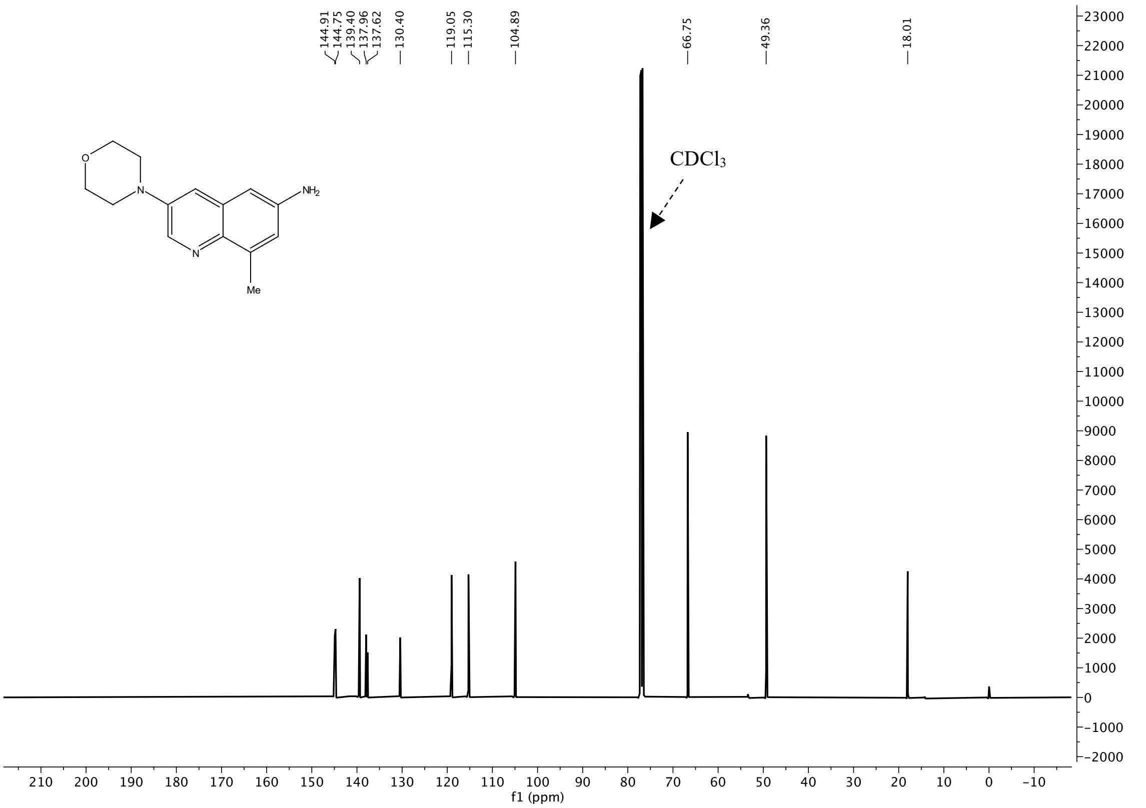

^{1}H (500 MHz) and ^{13}C (125 MHz) NMR spectra of the product obtained after the reduction of **5**.

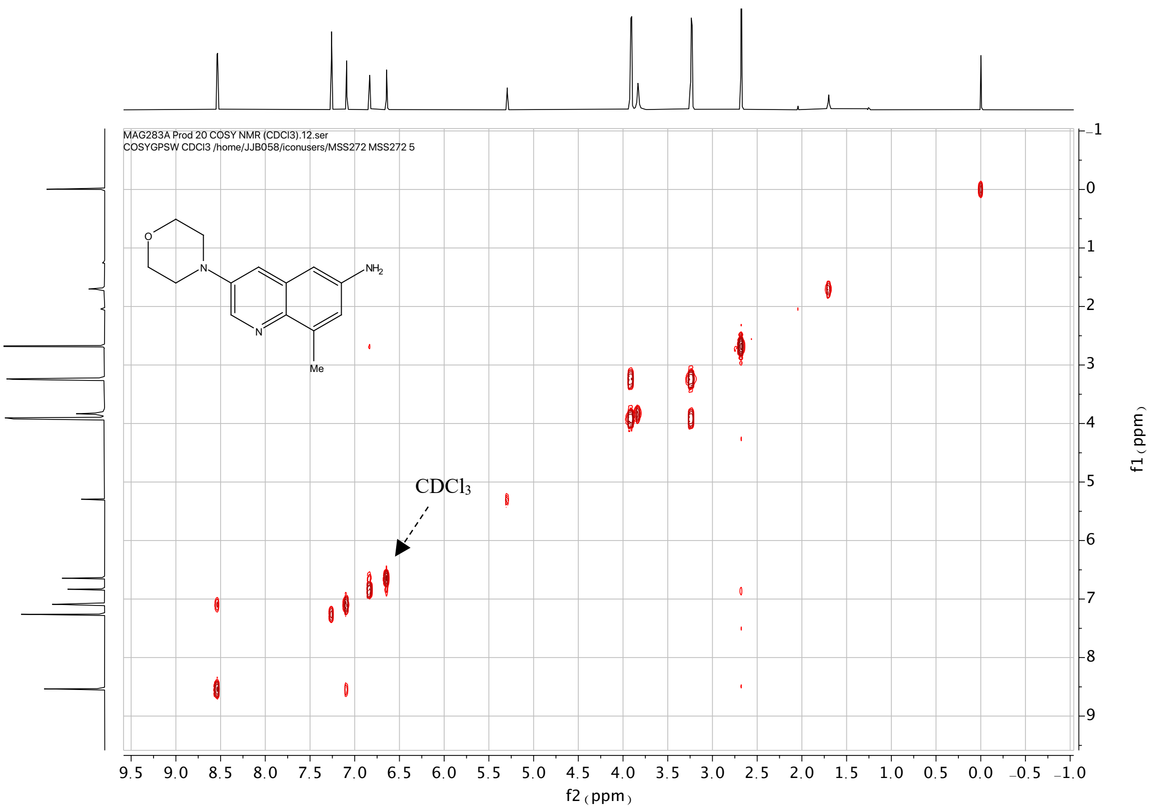

gCOSY (500 MHz) spectra of the product obtained after the reduction of **5**.

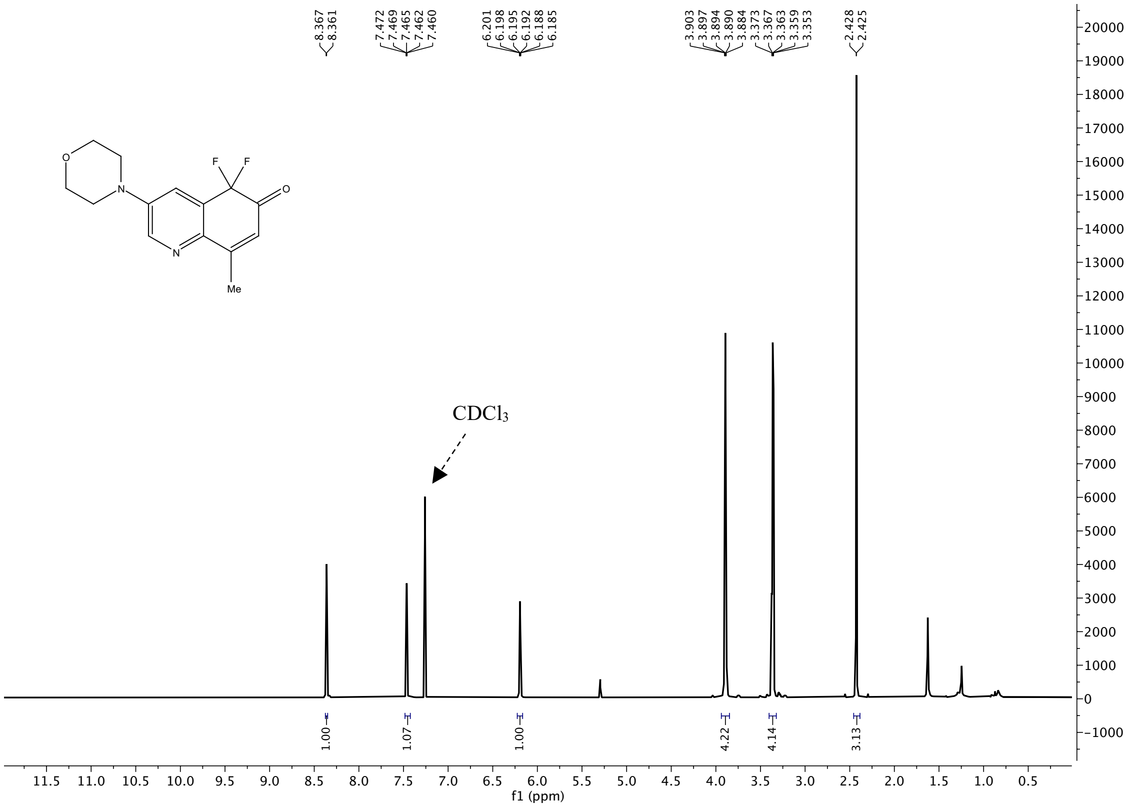

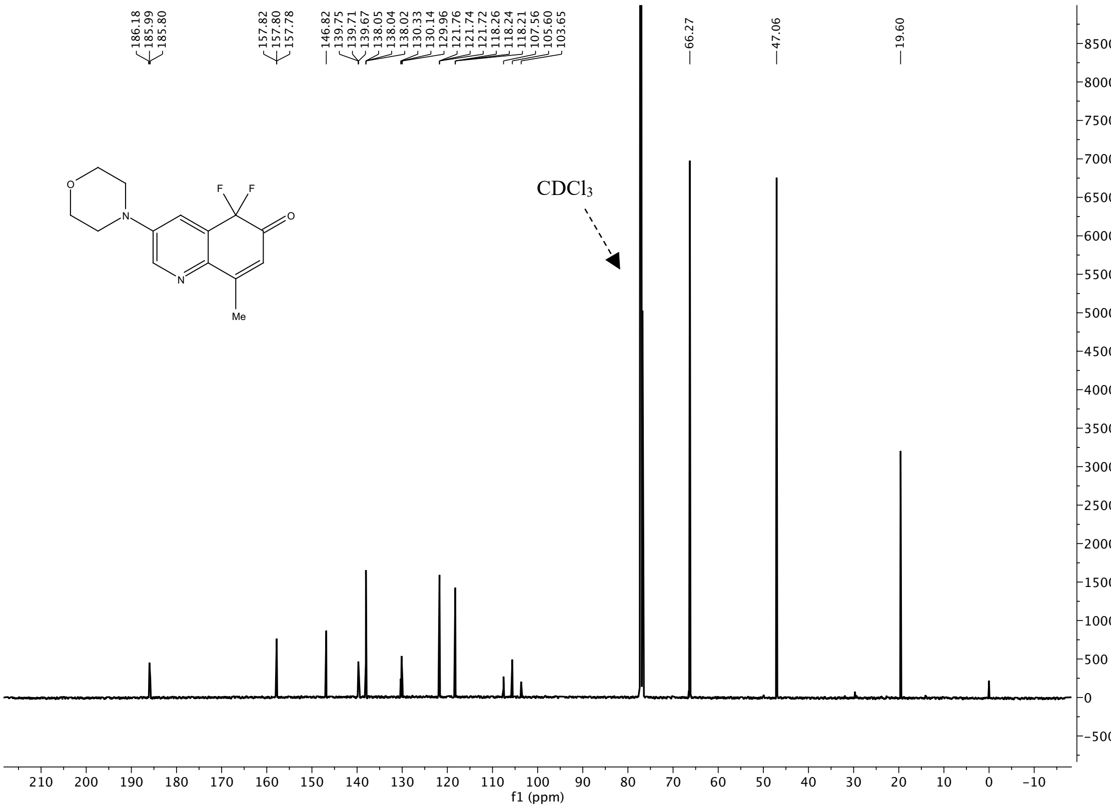

^{1}H (500 MHz) and ^{13}C (125 MHz) NMR spectra of **FC11**.

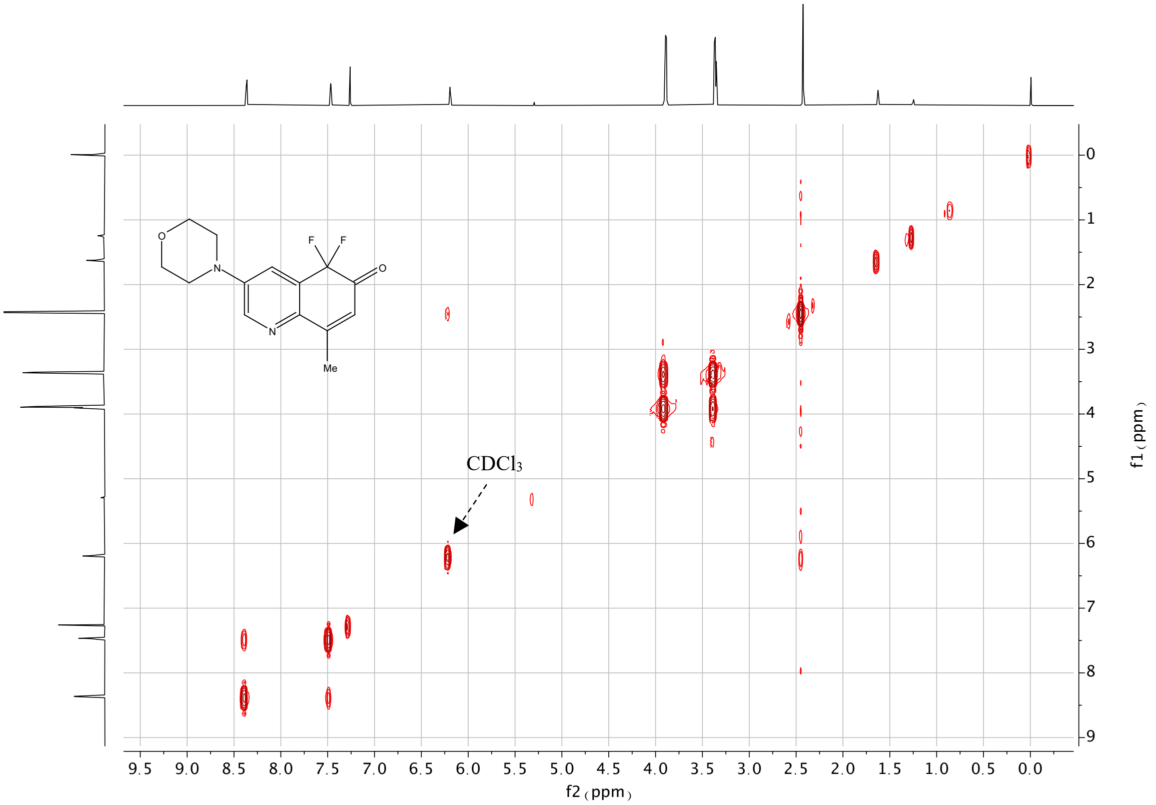

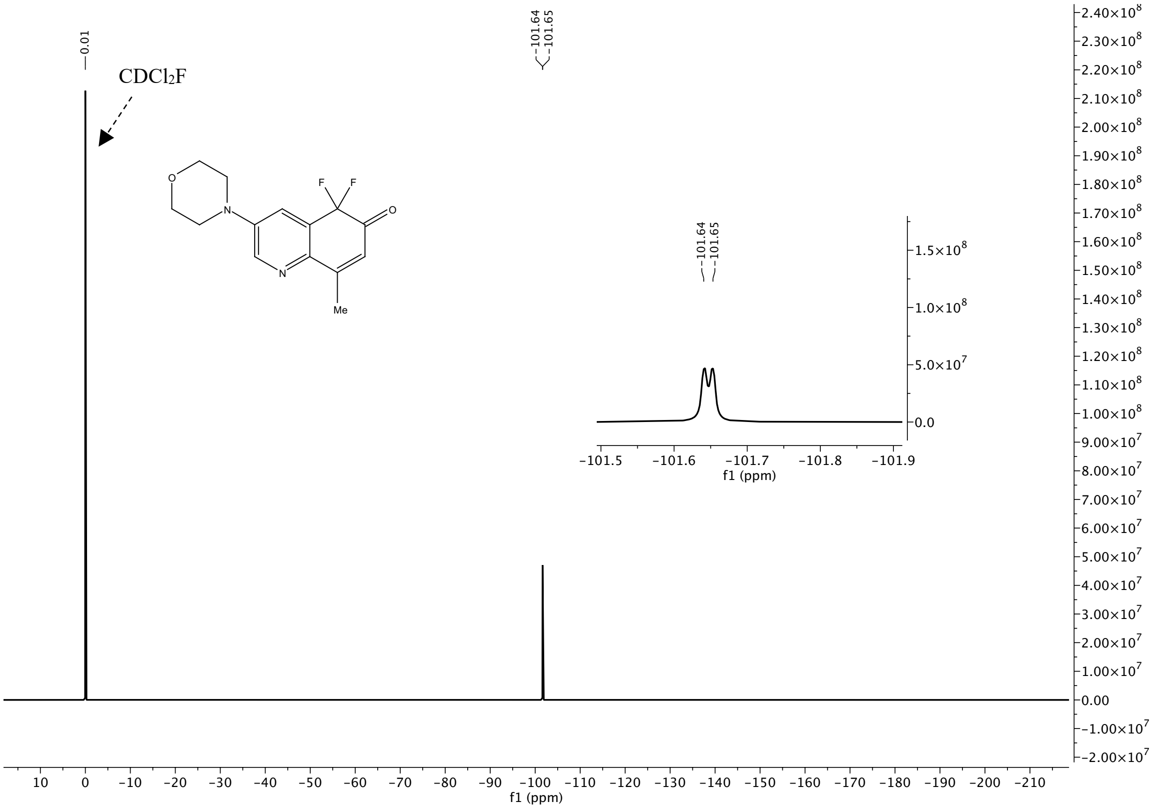

gCOSY (500 MHz) and ^{19}F (376 MHz) NMR spectra of **FC11**.

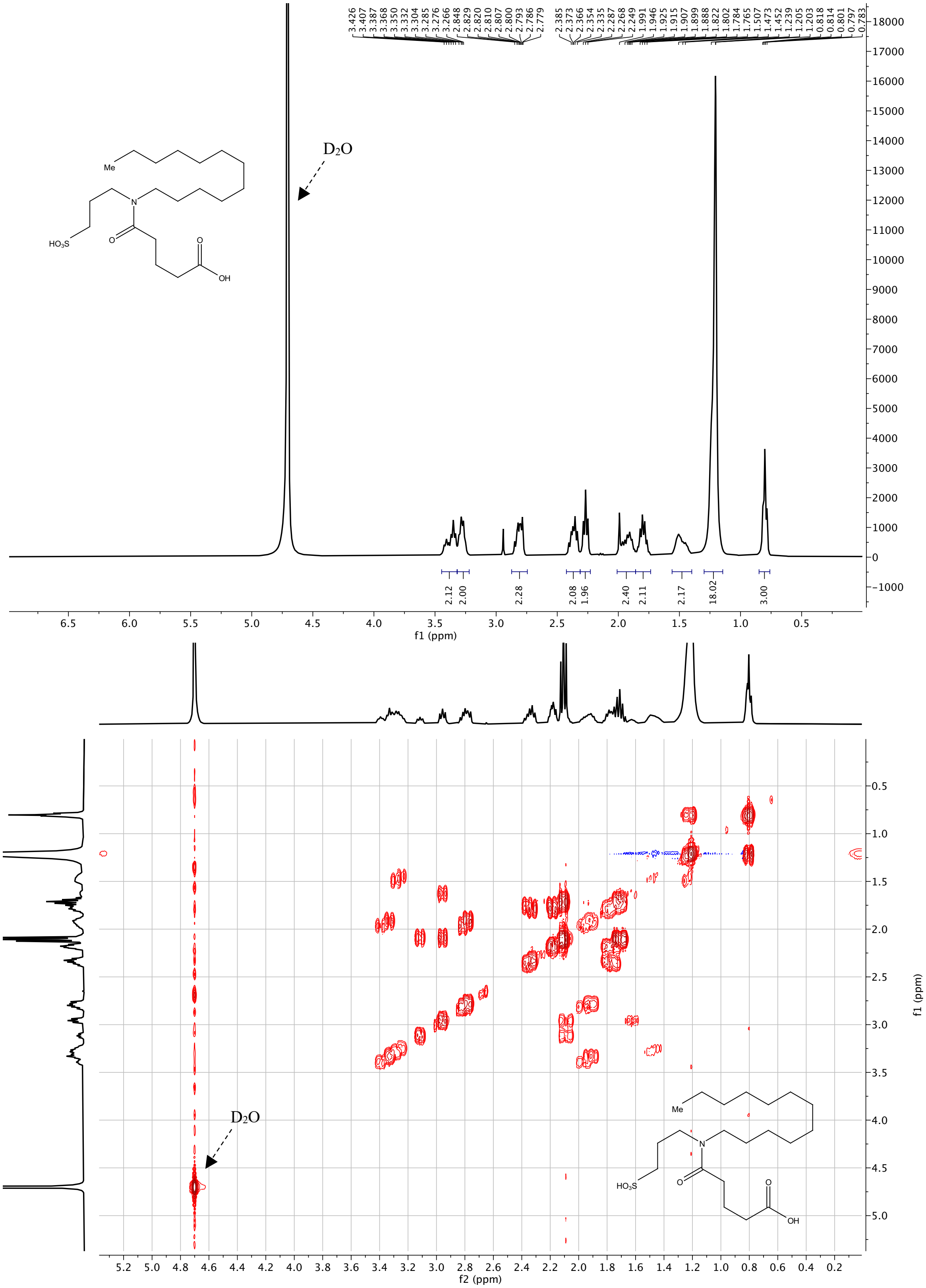

^{1}H (400 MHz) and gCOSY (400 MHz) NMR of **7**.

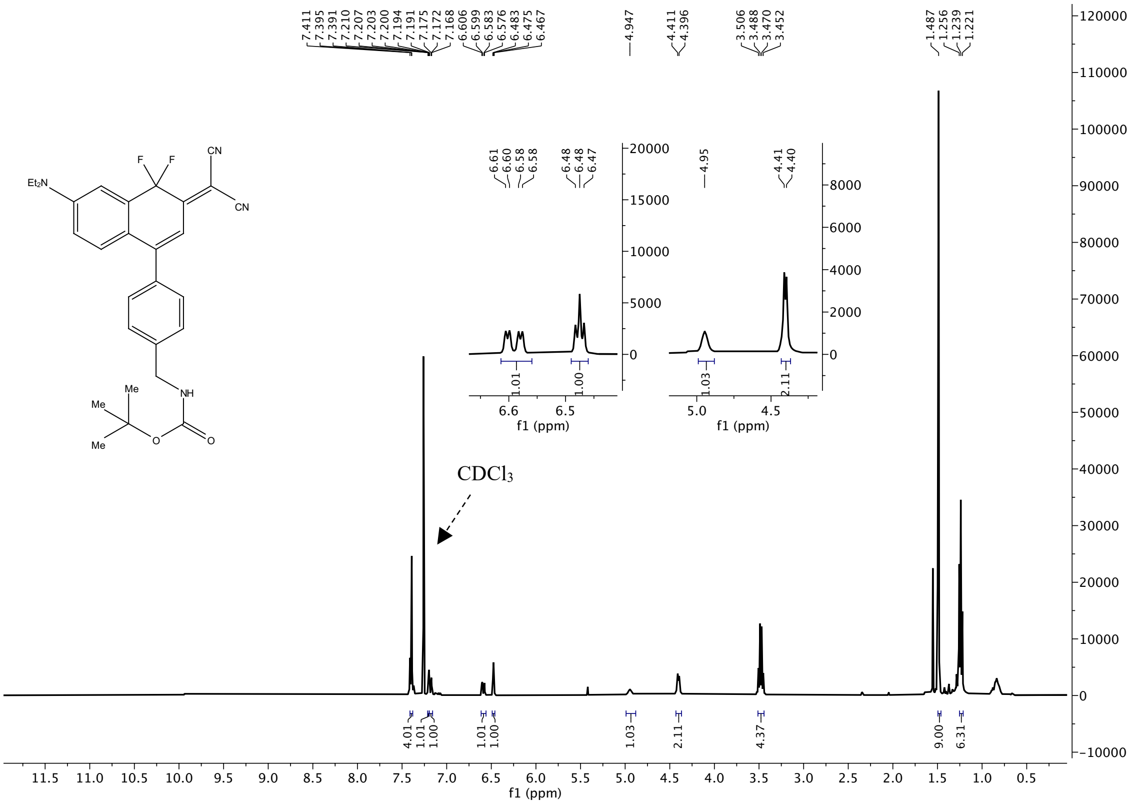

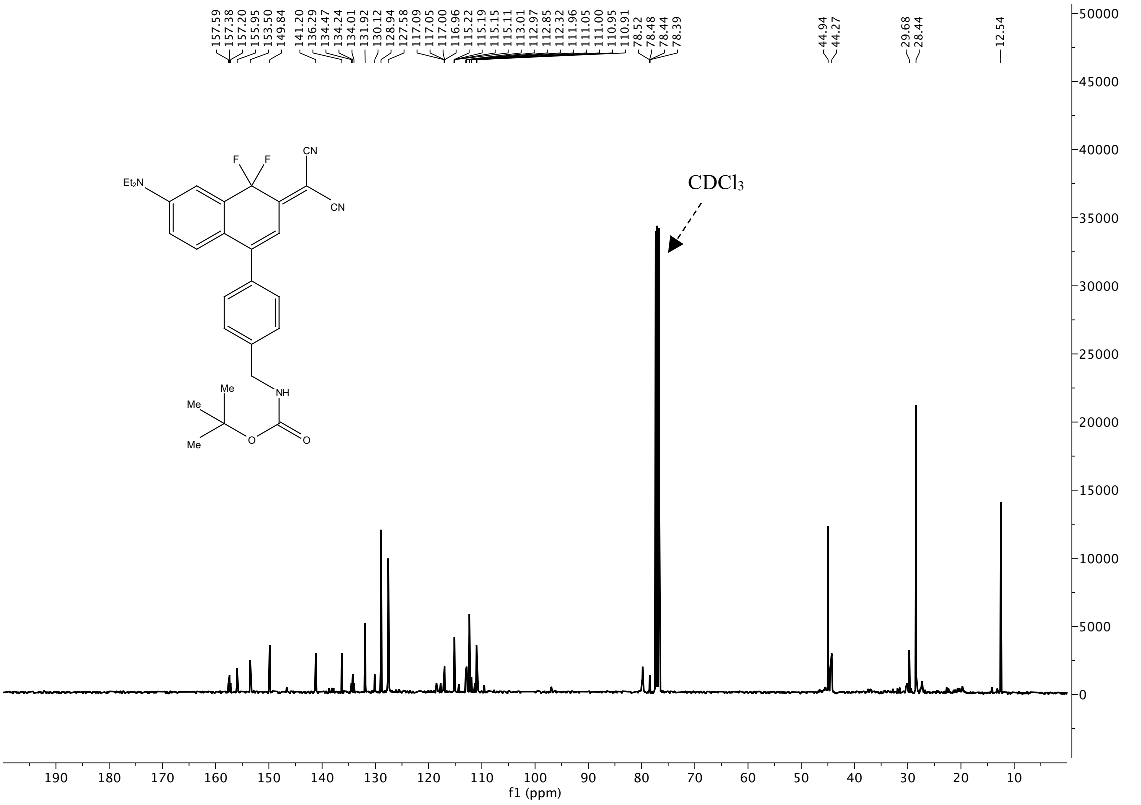

^{1}H (400 MHz) and ^{13}C (100 MHz) NMR spectra of **10**.

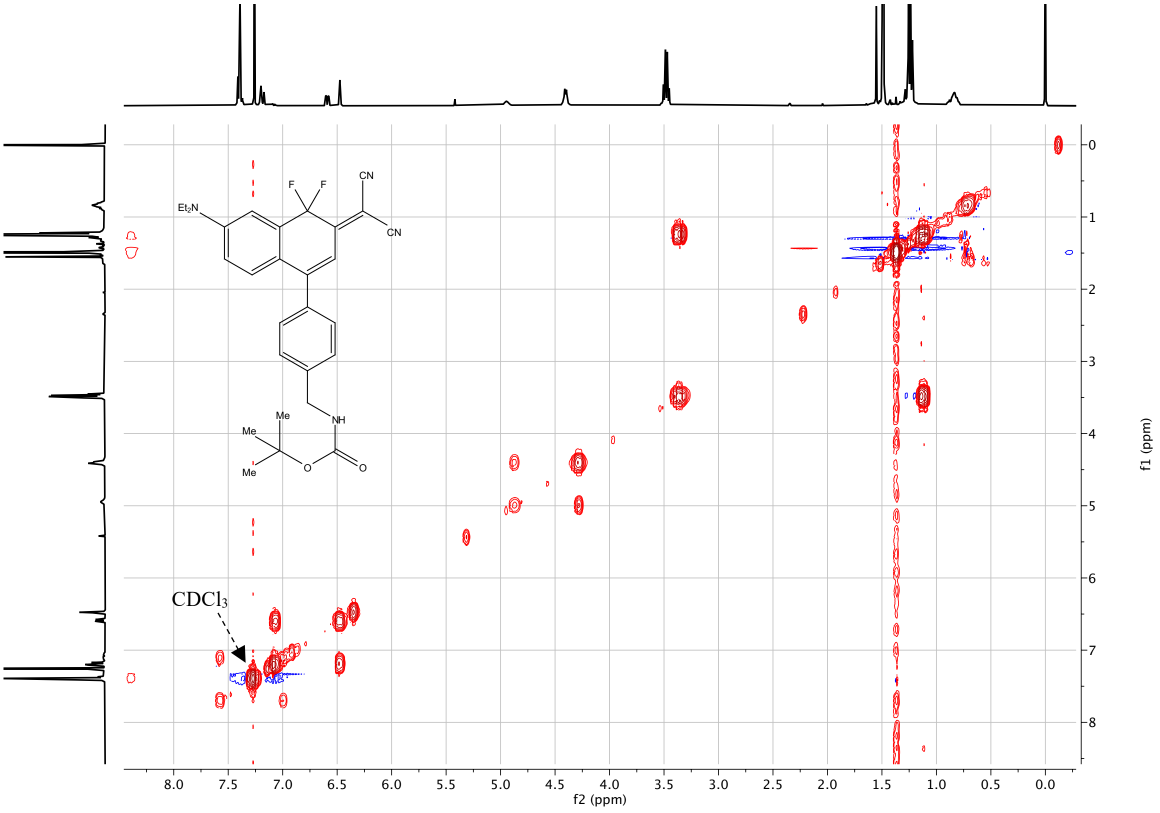

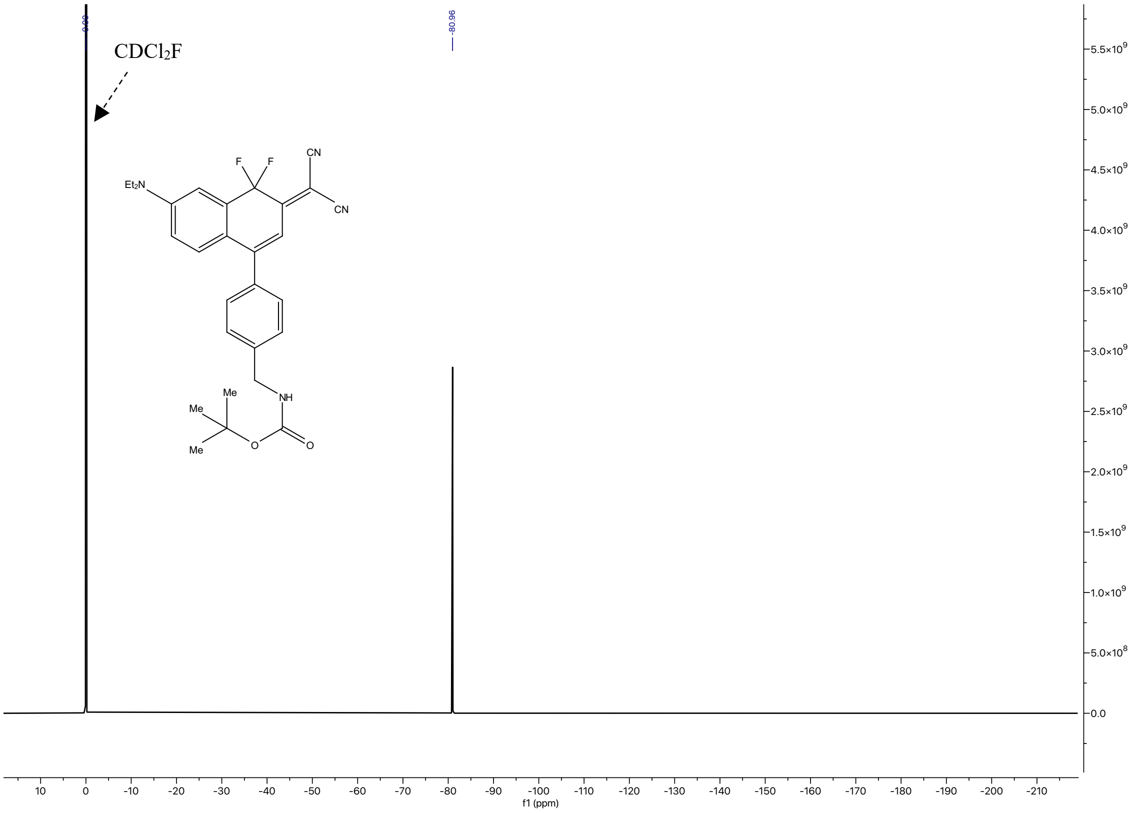

gCOSY (400 MHz) and ^{19}F (376 MHz) NMR spectra of **10**.

Section S9: Raw Data from Computational Analysis

Energies (hartree), oscillator strengths (S0 → S1*), HOMO & LUMO orbital energies, excitation/de-excitation amplitudes, and ground state optimized structures (Å)

a. C1

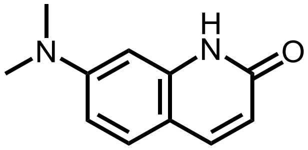

E(S0) = -611.007863
E(S1) = -610.874667*
Oscillator strength = 0.4775
E(HOMO) = -0.1971; E(LUMO) = -0.0498

OCC I	VIR A	EXCITATION AMPLITUDE X(I->A)	DE-EXCITATION Y(A->I)
50	51	0.980890	-0.066118
49	52	0.150171	0.029857
50	52	-0.046781	-0.003400
47	53	0.056809	0.008881
49	53	-0.071966	-0.022543
50	53	0.059285	0.015921

ATOM	CHARGE	X	Y	Z
C	6.0	-1.2449960515	0.6282005680	0.0321976523
C	6.0	-2.2802732069	-0.3340204919	0.0185899168
C	6.0	-1.9216424395	-1.7116836696	0.0105406724
C	6.0	-0.6249475033	-2.1146286891	0.0141366278
C	6.0	0.4557041105	-1.1538642427	0.0263302588
N	7.0	0.0576772019	0.1714342830	0.0358166435
H	1.0	-0.3497140216	-3.1588494054	0.0074351239
C	6.0	-2.8503838429	2.4537817902	0.0334328891
C	6.0	-3.8941706220	1.4817529076	0.0243404306
C	6.0	-3.6044138346	0.1401616169	0.0166024423
C	6.0	-1.5216087938	1.9926379455	0.0429025029
H	1.0	-4.9256926659	1.7952413143	0.0258724519
H	1.0	-4.4140816102	-0.5791768699	0.0102135751
H	1.0	-0.6954964399	2.6868345625	0.0569919114
N	7.0	-3.1358206349	3.7891629367	0.0316837151
O	8.0	1.6608055477	-1.4384381482	0.0282870148
H	1.0	-2.7157561717	-2.4494564039	0.0010379699

C	6.0	-4.5144480933	4.2535944782	0.0723204871
C	6.0	-2.0612782422	4.7663608714	0.1066173295
H	1.0	-5.0894371243	3.8813752690	-0.7786939293
H	1.0	-4.5214617033	5.3383882763	0.0283731914
H	1.0	-5.0257119609	3.9483817055	0.9904730473
H	1.0	-1.4856571792	4.6693075064	1.0326526728
H	1.0	-1.3698812841	4.6670920185	-0.7343478669
H	1.0	-2.4859800098	5.7648155652	0.0748809886
H	1.0	0.8088136757	0.8478831653	0.0435925708

b. C2

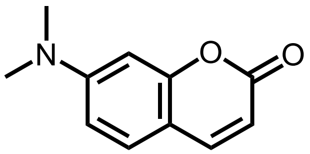

E(S0) = -630.865253
E(S1) = -630.738857*
Oscillator strength = 0.5260
E(HOMO) = -0.2018; E(LUMO) = -0.0631

OCC I	VIR A	EXCITATION AMPLITUDE X(I->A)	DE-EXCITATION AMPLITUDE Y(A->I)
49	51	0.035900	0.011138
50	51	0.990419	-0.076881
49	52	-0.097160	-0.026838
48	53	-0.049763	-0.008599
49	53	0.044819	0.017033
50	53	0.071601	0.019173

ATOM	CHARGE	X	Y	Z
C	6.0	-1.2490153828	0.6185831626	-0.0027192434
C	6.0	-2.2723001433	-0.3441182315	0.0073965511
C	6.0	-1.8990672652	-1.7152616015	-0.0128725843
C	6.0	-0.5943160860	-2.0861608428	-0.0389942488
C	6.0	0.4555901547	-1.1025073352	-0.0482815690
O	8.0	0.0643368325	0.2288908349	-0.0311645871
H	1.0	-0.2880494335	-3.1208445953	-0.0539009285
C	6.0	-2.8331784189	2.4438821927	0.0514426706
C	6.0	-3.8786394478	1.4749916669	0.0577230265
C	6.0	-3.5954912754	0.1317026805	0.0368614329
C	6.0	-1.5016002250	1.9759633877	0.0151189016
H	1.0	-4.9088615892	1.7917875337	0.0776181494
H	1.0	-4.4069742713	-0.5851539161	0.0418297065

H	1.0	-0.6630029160	2.6532742158	0.0039562329
N	7.0	-3.1089602053	3.7767316607	0.0804199449
O	8.0	1.6528099361	-1.3153424685	-0.0698535254
H	1.0	-2.6777521834	-2.4685998394	-0.0062586134
C	6.0	-4.4865610641	4.2489709409	0.0928791043
C	6.0	-2.0294303620	4.7514007612	0.0219636329
H	1.0	-5.0256440901	3.9605530052	-0.8140612706
H	1.0	-4.4867088169	5.3326508717	0.1548005203
H	1.0	-5.0346253098	3.8629680047	0.9552391743
H	1.0	-1.3387173069	4.6359782058	0.8612068642
H	1.0	-1.4563857803	4.6654010007	-0.9058868714
H	1.0	-2.4510187305	5.7503674450	0.0683910095

c. C3

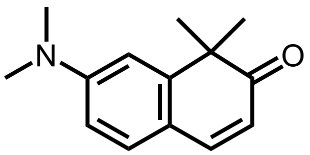

E(S0) = -673.518134
E(S1) = -673.404551*
Oscillator strength = 0.4345
E(HOMO) = -0.1948; E(LUMO) = -0.0683

OCC I	VIR A	EXCITATION AMPLITUDE X(I->A)	DE-EXCITATION AMPLITUDE Y(A->I)
58	59	-0.993474	0.080576
56	60	-0.058431	-0.022309
58	60	-0.076028	-0.006210
55	61	-0.036370	-0.006703
58	61	0.060395	0.020999

ATOM	CHARGE	X	Y	Z
C	6.0	-1.2427124474	0.7026350079	-0.0124547750
C	6.0	-2.2716661289	-0.2636390370	0.0023146858
C	6.0	-1.9535828152	-1.6611913768	-0.0245652284
C	6.0	-0.6878499717	-2.1388109668	-0.0636551752
C	6.0	0.4508682212	-1.2515495515	-0.0806314466
C	6.0	0.2259065977	0.2781442943	-0.0535151905
H	1.0	-0.4834763146	-3.2011928601	-0.0826159784
C	6.0	-2.9200520672	2.4924067500	0.0601258420
C	6.0	-3.9375212464	1.5062964307	0.0687475244
C	6.0	-3.6058750421	0.1710009782	0.0413382584
C	6.0	-1.5769760375	2.0462559895	0.0129744317

H	1.0	-4.9774758096	1.7905117659	0.0947530426
H	1.0	-4.3959177416	-0.5703880164	0.0483636704
H	1.0	-0.7885531730	2.7815188493	0.0005893305
N	7.0	-3.2216730849	3.8236678114	0.0982811788
O	8.0	1.6039574471	-1.6862153611	-0.1142407371
H	1.0	-2.7841975882	-2.3590627384	-0.0124658623
C	6.0	-4.6086106214	4.2615687034	0.1390102399
C	6.0	-2.1687328492	4.8259551030	0.0322002750
H	1.0	-5.1546982274	3.9796132380	-0.7664996712
H	1.0	-4.6354119130	5.3432676146	0.2265331790
H	1.0	-5.1348986800	3.8407248248	0.9989328000
H	1.0	-1.4721685693	4.7362515394	0.8701022751
H	1.0	-1.5952934145	4.7535669663	-0.8966468608
H	1.0	-2.6181527575	5.8131716754	0.0742031549
C	6.0	0.9041942847	0.8442878657	-1.3241459057
C	6.0	0.9593883134	0.8049863955	1.2040897838
H	1.0	0.3887356660	0.5103613445	-2.2259114873
H	1.0	1.9369614499	0.5021721394	-1.3678499774
H	1.0	0.8959457041	1.9333510315	-1.3123864178
H	1.0	0.9428093758	1.8936130577	1.2312301904
H	1.0	1.9954118937	0.4704379491	1.1889226298
H	1.0	0.4885717169	0.4351292426	2.1162355914

d. C4

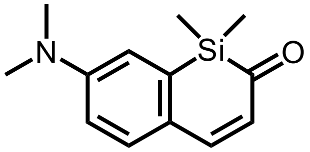

E(S0) = -924.916403
E(S1) = -924.817784 (HOMO-1 → LUMO; n → π*)*
Oscillator strength = 0.0016

OCC I	VIR A	EXCITATION AMPLITUDE X(I->A)	DE-EXCITATION AMPLITUDE Y(A->I)
61	63	0.986528	-0.029890
62	63	0.034317	-0.002589
61	65	0.147192	-0.015452

E(S2) = -924.812109 (HOMO → LUMO; π → π*)*
Oscillator strength = 0.3885
E(HOMO) = -0.1961; E(LUMO) = -0.0787

EXCITATION DE-EXCITATION

OCC I	VIR A	AMPLITUDE X(I->A)	AMPLITUDE Y(A->I)
61	63	0.033824	-0.001349
62	63	-0.992352	0.083293
60	64	0.054671	0.024442
62	64	0.082603	0.008256
62	65	-0.052652	-0.022500

ATOM	CHARGE	X	Y	Z
C	6.0	0.0899882359	-0.0343066970	0.0032042367
C	6.0	0.1674234951	-1.4517980368	0.0138176881
C	6.0	1.4083451866	-2.1954940066	0.0119004145
C	6.0	2.6958924496	-1.7532080644	-0.0066656547
C	6.0	3.0781950685	-0.3561917454	-0.0267418862
SI	14.0	1.6850951126	0.9727483738	-0.0022271212
H	1.0	3.5028311330	-2.4799108938	-0.0094766400
C	6.0	-2.3684528839	-0.1474403758	0.0040352620
C	6.0	-2.2707498197	-1.5586579969	0.0183712654
C	6.0	-1.0380665577	-2.1755932531	0.0228324631
C	6.0	-1.1500195248	0.5827365526	-0.0011756935
H	1.0	-3.1603187752	-2.1689583358	0.0253378064
H	1.0	-1.0002808715	-3.2586469175	0.0327354760
H	1.0	-1.1958670383	1.6624650114	-0.0104940880
N	7.0	-3.5764881515	0.4839902063	-0.0049146983
O	8.0	4.2701280938	-0.0089197787	-0.0535354284
H	1.0	1.2727949290	-3.2729835108	0.0239889075
C	6.0	-4.8089690170	-0.2909842814	-0.0106118127
C	6.0	-3.6568470709	1.9376147586	-0.0191614474
H	1.0	-4.8955197188	-0.9176546205	0.8811440097
H	1.0	-4.8731779213	-0.9379772603	-0.8895489200
H	1.0	-5.6543818550	0.3894951425	-0.0290404224
H	1.0	-3.1867611324	2.3607880978	-0.9111401203
H	1.0	-3.1791980161	2.3773356330	0.8603913268
H	1.0	-4.7017075966	2.2305588822	-0.0170882911
C	6.0	1.8571950696	2.0007289284	1.5617442762
C	6.0	1.8239624174	2.0407619206	-1.5416616036
H	1.0	1.7576000308	1.3813127305	2.4540963610
H	1.0	1.7234502899	1.4410335075	-2.4473701685
H	1.0	1.0926116773	2.7793798503	1.6019704601
H	1.0	1.0475374902	2.8084401564	-1.5554382410
H	1.0	2.8342300276	2.4868245935	1.5884967628
H	1.0	2.7932277638	2.5421524494	-1.5677744791

e. C5

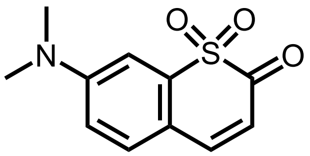

E(S0) = -1104.202520
E(S1) = -1104.106157*
Oscillator strength = 0.3600
E(HOMO) = -0.2143; E(LUMO) = -0.1064

OCC I	VIR A	EXCITATION AMPLITUDE X(I->A)	DE-EXCITATION AMPLITUDE Y(A->I)
59	63	0.042644	-0.018469
60	63	-0.052066	-0.011152
62	63	-0.991849	0.099316
60	64	0.057035	0.026658
62	64	0.080376	0.008465
59	65	0.031778	0.007060
62	65	-0.050524	-0.022992
61	67	-0.053477	-0.024489
61	68	0.032626	0.014960

ATOM	CHARGE	X	Y	Z
C	6.0	-1.2066070859	0.7918590702	0.0064252052
C	6.0	-2.1746871458	-0.2327000812	0.0029025275
C	6.0	-1.8624197873	-1.6275186366	-0.0217596929
C	6.0	-0.6373534859	-2.2263733282	-0.0532018871
C	6.0	0.5995227939	-1.5203829359	-0.0719658995
S	16.0	0.5251946674	0.3637052042	-0.0272196225
H	1.0	-0.5621898000	-3.3059146198	-0.0710512889
C	6.0	-2.8875544091	2.5384589776	0.0449563767
C	6.0	-3.8733300812	1.5164556021	0.0360147885
C	6.0	-3.5187402981	0.1894615946	0.0183286368
C	6.0	-1.5263365247	2.1270850861	0.0266631736
H	1.0	-4.9200832078	1.7740991703	0.0400674414
H	1.0	-4.2965264648	-0.5632298897	0.0116703524
H	1.0	-0.7368710502	2.8618716443	0.0279888423
N	7.0	-3.2199519042	3.8493707546	0.0688024863
O	8.0	1.7288056969	-1.9570635675	-0.1088773966
H	1.0	-2.7222256874	-2.2877596641	-0.0163708514
C	6.0	-4.6213211607	4.2544973519	0.0915448322
C	6.0	-2.1886395434	4.8817456635	0.0442673805
H	1.0	-5.1410566845	3.9562074433	-0.8224797890

H	1.0	-4.6723083243	5.3345798312	0.1766765689
H	1.0	-5.1433712867	3.8193412694	0.9455284865
H	1.0	-1.5306507812	4.8097341451	0.9131877227
H	1.0	-1.5794797273	4.8173358989	-0.8602559071
H	1.0	-2.6670401039	5.8550481802	0.0617532655
O	8.0	1.1427107647	0.7827781524	1.2176078522
O	8.0	1.1018247618	0.8539306425	-1.2651649347

f. C6

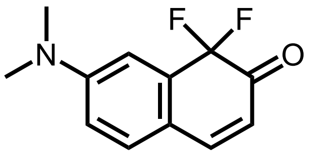

E(S0) = -793.411101
E(S1) = -793.315160*
Oscillator strength = 0.3271
E(HOMO) = -0.2048; E(LUMO) = -0.0960

OCC I	VIR A	EXCITATION AMPLITUDE X(I->A)	DE-EXCITATION AMPLITUDE Y(A->I)
55	59	0.036720	-0.022308
56	59	0.045850	0.012699
58	59	0.994337	-0.096403
56	60	0.048877	0.024567
58	60	0.087511	0.009893
55	61	0.033767	0.008622
58	61	0.045264	0.022498

ATOM	CHARGE	X	Y	Z
C	6.0	-1.2150916568	0.6937045596	-0.0081031671
C	6.0	-2.2239859625	-0.2867169813	0.0050298609
C	6.0	-1.8884359040	-1.6821245745	-0.0116595011
C	6.0	-0.6185523047	-2.1636896922	-0.0392532341
C	6.0	0.5106647648	-1.2781227143	-0.0548267265
C	6.0	0.2242580056	0.2538495078	-0.0410067613
H	1.0	-0.4157731044	-3.2256226695	-0.0497106179
C	6.0	-2.8593970338	2.4864175915	0.0430127870
C	6.0	-3.8739506866	1.4959698498	0.0511533519
C	6.0	-3.5544610709	0.1556226392	0.0337357353
C	6.0	-1.5122629863	2.0374024959	0.0082751064
H	1.0	-4.9130402455	1.7833933123	0.0691334492
H	1.0	-4.3514792898	-0.5772874599	0.0405271823
H	1.0	-0.7038200626	2.7512695013	-0.0025454229

N	7.0	-3.1554573278	3.8122521017	0.0684991609
O	8.0	1.6838169483	-1.6249894614	-0.0785230267
H	1.0	-2.7140577956	-2.3847506571	-0.0007962360
C	6.0	-4.5426942157	4.2546090378	0.1052722581
C	6.0	-2.0966787673	4.8123954038	0.0132371605
H	1.0	-5.0861108875	3.9610769631	-0.7972904099
H	1.0	-4.5661134182	5.3369928196	0.1787491019
H	1.0	-5.0671783434	3.8441303313	0.9708281592
H	1.0	-1.4149108303	4.7235357889	0.8625754392
H	1.0	-1.5127617205	4.7310218195	-0.9072821119
H	1.0	-2.5446761734	5.8002327306	0.0429073284
F	9.0	0.8515397691	0.7878961033	-1.1474912427
F	9.0	0.8985731102	0.7761396432	1.0432362073

g. C7

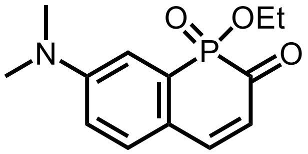

E(S0) = -1126.653876
E(S1) = -1126.558203*
Oscillator strength = 0.3232
E(HOMO) = -0.2064; E(LUMO) = -0.0979

OCC I	VIR A	EXCITATION AMPLITUDE X(I->A)	DE-EXCITATION AMPLITUDE Y(A->I)
67	71	0.040564	-0.019068
68	71	-0.037149	-0.012360
69	71	-0.112009	-0.000241
70	71	0.987042	-0.092378
68	72	0.048794	0.023050
70	72	-0.079255	-0.007694
67	73	0.032855	0.008294
70	73	0.051394	0.023597

ATOM	CHARGE	X	Y	Z
C	6.0	-1.1771501626	0.7372436787	0.0250456205
C	6.0	-2.1816112033	-0.2555507922	-0.0583540146
C	6.0	-1.9136713503	-1.6695097560	-0.0505031983
C	6.0	-0.7177418261	-2.3091776528	0.0678338781
C	6.0	0.5400898271	-1.6273668194	0.2103456902
P	15.0	0.5515066732	0.2403614678	0.1347581899
H	1.0	-0.6835329618	-3.3921457091	0.0763399819

C	6.0	-2.8407331196	2.5243594719	0.0032073998
C	6.0	-3.8432953703	1.5279734158	-0.0940619291
C	6.0	-3.5123928586	0.1915185653	-0.1222872230
C	6.0	-1.4921258536	2.0798165679	0.0595503354
H	1.0	-4.8837265426	1.8068629562	-0.1444850938
H	1.0	-4.3057987799	-0.5423070293	-0.1897978921
H	1.0	-0.6951714109	2.8022390907	0.1450271607
N	7.0	-3.1484140129	3.8457943731	0.0460914785
O	8.0	1.6259970894	-2.1892809930	0.3511316080
H	1.0	-2.7950592202	-2.2960307559	-0.1352929366
C	6.0	-4.5406277642	4.2755333783	0.0527881383
C	6.0	-2.0984643708	4.8540554738	0.1287637623
H	1.0	-5.0511500890	4.0064154807	-0.8756370876
H	1.0	-4.5757884071	5.3547749790	0.1588117293
H	1.0	-5.0890018349	3.8327614981	0.8874895395
H	1.0	-1.5350923727	4.7772273718	1.0626583668
H	1.0	-1.3986468899	4.7686607790	-0.7051787836
H	1.0	-2.5533561658	5.8382889569	0.0867880736
O	8.0	1.3350653929	0.8593443158	1.2299172846
O	8.0	1.0780188576	0.5487510061	-1.3410217423
C	6.0	2.4754793417	0.3597490198	-1.7229102104
C	6.0	2.6585145543	0.9195525461	-3.1145070854
H	1.0	3.6978747837	0.7942642079	-3.4213840212
H	1.0	2.4179119644	1.9824726939	-3.1399185693
H	1.0	2.0236266581	0.3982354168	-3.8312093118
H	1.0	3.1012756798	0.8767035193	-0.9965909594
H	1.0	2.7008298248	-0.7059074230	-1.6858994789

Energies (hartree), oscillator strengths (S1 → S0*), and excited state (S1) optimized structures (Å).

a. C1

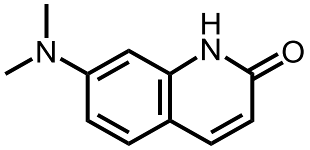

E(S0) = -611.003722*
E(S1) = -610.884291
Oscillator strength = 0.7090

ATOM	CHARGE	X	Y	Z
C	6.0	-1.2459825248	0.6245145913	0.0288934560
C	6.0	-2.2959546852	-0.3491740862	0.0321316429
C	6.0	-1.9411182239	-1.7299792177	0.0200474330
C	6.0	-0.5907167614	-2.1106149639	0.0089062447
C	6.0	0.4650746421	-1.1661758758	0.0030879125
N	7.0	0.0607670395	0.1833903788	0.0096068781
H	1.0	-0.3101235114	-3.1548699811	0.0021421390
C	6.0	-2.8776051520	2.4425277206	0.0553734239
C	6.0	-3.9228875919	1.4911535497	0.0580409688
C	6.0	-3.6273919843	0.1378148423	0.0485869842
C	6.0	-1.5325889660	1.9774029479	0.0427180203
H	1.0	-4.9536087389	1.8058174707	0.0781944345
H	1.0	-4.4370353317	-0.5800340518	0.0554378365
H	1.0	-0.7131126285	2.6791884813	0.0338015025
N	7.0	-3.1342632484	3.7913708560	0.0614906003
O	8.0	1.6962522620	-1.4113366410	-0.0075412663
H	1.0	-2.7223995948	-2.4767853126	0.0231034278
C	6.0	-4.5016819380	4.2759538486	0.0090858379
C	6.0	-2.0573938822	4.7652430000	0.1551536925
H	1.0	-5.0268811279	3.8559744108	-0.8513774513
H	1.0	-4.4966816527	5.3567824375	-0.0746881377
H	1.0	-5.0523422108	3.9961181300	0.9130572861
H	1.0	-1.4541362516	4.5960143218	1.0496658128
H	1.0	-1.4007831606	4.7144395236	-0.7175304583
H	1.0	-2.4849717975	5.7600536953	0.2071187802
H	1.0	0.8077141208	0.8614987838	0.0077732890

b. C2

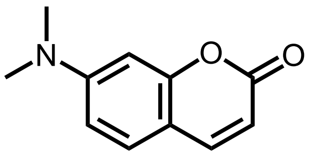

E(S0) = -630.861335*

E(S1) = -630.748981

Oscillator strength = 0.7103

ATOM	CHARGE	X	Y	Z
C	6.0	-1.2479528399	0.6107643601	-0.0002515474
C	6.0	-2.2833776692	-0.3559480821	-0.0048925783
C	6.0	-1.9243581681	-1.7424706529	-0.0221554577
C	6.0	-0.5698727004	-2.0760508330	-0.0302177529
C	6.0	0.4660066714	-1.1154882169	-0.0253190673
O	8.0	0.0735933133	0.2539999637	-0.0105748124
H	1.0	-0.2527450275	-3.1096633958	-0.0415585405
C	6.0	-2.8523675382	2.4361165917	0.0361932500
C	6.0	-3.9007108573	1.4823475972	0.0307302744
C	6.0	-3.6110774299	0.1339362345	0.0093030551
C	6.0	-1.5135479358	1.9637225933	0.0172152919
H	1.0	-4.9309375328	1.7991678894	0.0349747747
H	1.0	-4.4232892482	-0.5810184493	0.0017740155
H	1.0	-0.6778625930	2.6449057781	0.0258478076
N	7.0	-3.1090816740	3.7823572149	0.0617756327
O	8.0	1.6863690596	-1.2952045854	-0.0328131733
H	1.0	-2.6914545804	-2.5019442946	-0.0272605837
C	6.0	-4.4757857779	4.2677780333	0.1456060461
C	6.0	-2.0293485744	4.7533864612	-0.0164914382
H	1.0	-5.0409703711	4.0036139036	-0.7536098692
H	1.0	-4.4665603106	5.3469841250	0.2468165014
H	1.0	-4.9864350929	3.8350002209	1.0082940815
H	1.0	-1.3676997057	4.6788189455	0.8511477848
H	1.0	-1.4317316965	4.5999981893	-0.9178314162
H	1.0	-2.4523651006	5.7509991486	-0.0438487984

c. C3

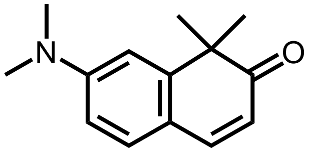

E(S0) = -673.514564*

E(S1) = -673.413615
Oscillator strength = 0.6095

ATOM	CHARGE	X	Y	Z
C	6.0	-1.2446883959	0.6784301957	-0.0187991936
C	6.0	-2.2945797941	-0.2879746841	-0.0051156443
C	6.0	-2.0072048527	-1.6958209562	-0.0310347544
C	6.0	-0.6934967408	-2.1201539837	-0.0686792051
C	6.0	0.4351432125	-1.2425300706	-0.0814746502
C	6.0	0.2319508563	0.2826819287	-0.0510231198
H	1.0	-0.4650656563	-3.1801095824	-0.0892565536
C	6.0	-2.9196571253	2.4787606965	0.0478992403
C	6.0	-3.9558029204	1.4997913753	0.0614045157
C	6.0	-3.6352268310	0.1697840728	0.0338860249
C	6.0	-1.5807166552	2.0248863643	0.0043963203
H	1.0	-4.9922134488	1.7959247655	0.0859030935
H	1.0	-4.4294706708	-0.5658416554	0.0404373473
H	1.0	-0.7893852879	2.7568192338	-0.0018651609
N	7.0	-3.2182304093	3.8119535096	0.0774698031
O	8.0	1.6142146348	-1.6887079173	-0.1128155534
H	1.0	-2.8261406781	-2.4009730564	-0.0217197953
C	6.0	-4.5984627124	4.2657410394	0.1766680706
C	6.0	-2.1656793328	4.8128405679	-0.0045656805
H	1.0	-5.1682405164	3.9982516700	-0.7175350550
H	1.0	-4.6092146585	5.3446990423	0.2826384780
H	1.0	-5.0915462403	3.8253818304	1.0447530702
H	1.0	-1.4879888424	4.7431557259	0.8506933344
H	1.0	-1.5790136940	4.6889656163	-0.9178961661
H	1.0	-2.6143192913	5.7996821792	-0.0108254455
C	6.0	0.9258807552	0.8747067379	-1.3071264177
C	6.0	0.9629340134	0.8273943896	1.2064707097
H	1.0	0.4115639465	0.5665226088	-2.2188081987
H	1.0	1.9520471142	0.5128454867	-1.3493683561
H	1.0	0.9446093807	1.9643724264	-1.2769808074
H	1.0	0.9760564247	1.9174888999	1.2195083950
H	1.0	1.9913942109	0.4695032267	1.2038963644
H	1.0	0.4778043754	0.4803749764	2.1202283602

d. C4 (data at geometry optimization step 32, see Figure S18 for full details)

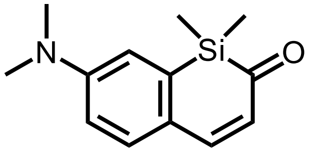

E(S0) = -924.912396*

E(S1) = -924.821667
Oscillator strength = 0.4763

ATOM	CHARGE	X	Y	Z
C	6.0	0.1206619189	-0.0740814473	0.0235209207
C	6.0	0.1655731887	-1.5097884054	0.0407133178
C	6.0	1.3869076192	-2.2757694184	0.0531576603
C	6.0	2.6662226820	-1.7630333330	-0.0048433116
C	6.0	3.0476089973	-0.3718689026	-0.0914750639
SI	14.0	1.6977106029	0.9487095031	-0.0021139089
H	1.0	3.4967693830	-2.4640789712	0.0461928495
C	6.0	-2.3417197053	-0.1562965355	-0.0279621407
C	6.0	-2.2752562882	-1.5825865489	0.0003094057
C	6.0	-1.0638499813	-2.2167858138	0.0372989796
C	6.0	-1.1206315655	0.5557456596	-0.0188184016
H	1.0	-3.1782319624	-2.1722450368	-0.0110810950
H	1.0	-1.0402510333	-3.2992531562	0.0527963917
H	1.0	-1.1544967346	1.6355482506	-0.0273719797
N	7.0	-3.5470766200	0.4806262470	-0.0414990529
O	8.0	4.2694636299	-0.0356869453	-0.1780508016
H	1.0	1.2740549247	-3.3514380354	0.0992936099
C	6.0	-4.7952943861	-0.2718623590	-0.0004778905
C	6.0	-3.6150033663	1.9341865173	-0.0619775521
H	1.0	-4.8612857496	-0.8815688626	0.9031866599
H	1.0	-4.8879283415	-0.9276596623	-0.8688683393
H	1.0	-5.6253829035	0.4255220578	-0.0068386614
H	1.0	-3.1171215083	2.3381757293	-0.9464461693
H	1.0	-3.1392004346	2.3624351420	0.8246231425
H	1.0	-4.6549118929	2.2392918498	-0.0788575279
C	6.0	1.8641641034	2.0103638416	1.5519300203
C	6.0	1.7627601800	2.1134168070	-1.4894089280
H	1.0	1.7992724731	1.4097326699	2.4600635217
H	1.0	1.6571518696	1.5653962286	-2.4269530785
H	1.0	1.0766603110	2.7675754640	1.5884797954
H	1.0	0.9727944712	2.8660390762	-1.4427133152
H	1.0	2.8235207732	2.5319161620	1.5525917546
H	1.0	2.7200478658	2.6389632481	-1.5084008115

e. C5

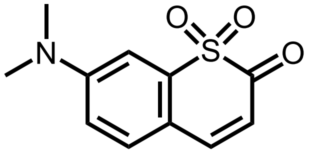

E(S0) = -1104.193043

E(S1) = -1104.117417*
Oscillator strength = 0.3153

ATOM	CHARGE	X	Y	Z
C	6.0	-1.1902784912	0.7708510595	-0.2120932044
C	6.0	-2.1913095265	-0.2485241854	-0.2062479289
C	6.0	-1.8756279169	-1.6566998289	-0.1295989384
C	6.0	-0.6315617899	-2.1245310028	0.1735632659
C	6.0	0.5552593746	-1.3093660689	0.3486296677
S	16.0	0.4892577559	0.3089105471	-0.4656379893
H	1.0	-0.4822658668	-3.1859868312	0.3359143515
C	6.0	-2.8560250028	2.5136915678	0.0136303779
C	6.0	-3.8659215874	1.5048486822	-0.0661607813
C	6.0	-3.5313053997	0.1779431714	-0.1702777178
C	6.0	-1.5067244060	2.1112244669	-0.0746710116
H	1.0	-4.9071455042	1.7770863560	-0.0040588322
H	1.0	-4.3152430700	-0.5669609384	-0.1830298861
H	1.0	-0.7108387497	2.8380528126	-0.0717385457
N	7.0	-3.2008761593	3.8204325576	0.1575459020
O	8.0	1.6027508914	-1.6430277559	0.9138610586
H	1.0	-2.6942131326	-2.3544576554	-0.2370465933
C	6.0	-4.5873690267	4.2639082789	0.0567921455
C	6.0	-2.1819133207	4.8287249145	0.4095411451
H	1.0	-5.0843565605	3.7953972551	-0.7900554449
H	1.0	-4.5990221631	5.3392583194	-0.0869112718
H	1.0	-5.1422363550	4.0304456855	0.9698679290
H	1.0	-1.5016240068	4.4952150696	1.1928612099
H	1.0	-1.6001540089	5.0334958645	-0.4947348416
H	1.0	-2.6642619625	5.7468356259	0.7269617268
O	8.0	1.3635914278	1.2564094347	0.2066380644
O	8.0	0.7187286979	0.1334455572	-1.8975051875

f. C6

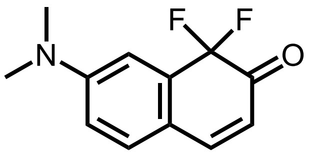

E(S0) = -793.403226*
E(S1) = -793.326623
Oscillator strength = 0.3242

ATOM	CHARGE	X	Y	Z
C	6.0	-1.1975170928	0.6708610387	0.0065801358

C	6.0	-2.2398666396	-0.3045982697	0.0161432966
C	6.0	-1.9385238070	-1.7135563122	-0.0115847514
C	6.0	-0.6424445885	-2.1264361414	-0.0658786358
C	6.0	0.4900557182	-1.2183785746	-0.0931981249
C	6.0	0.2204669675	0.2528244219	0.0019180716
H	1.0	-0.3964793482	-3.1805562217	-0.0981871041
C	6.0	-2.8396136585	2.4701986796	0.0410606489
C	6.0	-3.8783162425	1.4860415297	0.0551081329
C	6.0	-3.5725746467	0.1506939372	0.0420548115
C	6.0	-1.5036660541	2.0250894460	0.0167118720
H	1.0	-4.9130068318	1.7882900068	0.0644067727
H	1.0	-4.3720431936	-0.5779544032	0.0455326295
H	1.0	-0.6937810471	2.7359523605	0.0145544042
N	7.0	-3.1496284242	3.7954989764	0.0481082560
O	8.0	1.6718186613	-1.6156935675	-0.1743240285
H	1.0	-2.7549668925	-2.4215522890	0.0040553874
C	6.0	-4.5321966034	4.2536600901	0.1375482865
C	6.0	-2.0960144113	4.7954054209	-0.0510483140
H	1.0	-5.0864453893	4.0134284231	-0.7731201925
H	1.0	-4.5362379650	5.3297127098	0.2695446333
H	1.0	-5.0386139602	3.7979872917	0.9879257200
H	1.0	-1.4370465070	4.7549919021	0.8204171496
H	1.0	-1.4927518542	4.6317379443	-0.9462309789
H	1.0	-2.5444447935	5.7806362476	-0.1044920358
F	9.0	0.9279136524	0.9307630624	-1.0156719959
F	9.0	0.8838877619	0.7695602805	1.1597497836

g. C7

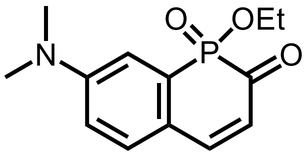

E(S0) = -1126.647853*
E(S1) = -1126.568391
Oscillator strength = 0.3733

ATOM	CHARGE	X	Y	Z
C	6.0	-1.1377684523	0.7993641186	-0.0117759081
C	6.0	-2.1436260901	-0.2060888409	-0.1839615556
C	6.0	-1.8578343803	-1.6219326351	-0.1919838114
C	6.0	-0.6356912651	-2.1604764874	0.0960617565
C	6.0	0.5844422463	-1.4260161582	0.3874288180
P	15.0	0.5928527228	0.3651176141	0.0927895697
H	1.0	-0.5296476509	-3.2403376733	0.1204889687
C	6.0	-2.8443949946	2.5442786781	0.0460435166

C	6.0	-3.8362995055	1.5418029414	-0.1857008151
C	6.0	-3.4834305906	0.2224067007	-0.2949569824
C	6.0	-1.4969533893	2.1357068442	0.1186329739
H	1.0	-4.8776565788	1.8135787252	-0.2499625702
H	1.0	-4.2561631379	-0.5203857947	-0.4410702844
H	1.0	-0.7189038605	2.8678470248	0.2662030781
N	7.0	-3.2034770641	3.8500956432	0.1811093562
O	8.0	1.6379105452	-1.9904912889	0.7716728178
H	1.0	-2.6896996765	-2.2849221352	-0.3880218140
C	6.0	-4.5796603749	4.2861573728	-0.0304192365
C	6.0	-2.2109753481	4.8547500781	0.5323676916
H	1.0	-4.9637510927	3.9140022316	-0.9796189671
H	1.0	-4.6034876253	5.3700498802	-0.0484906170
H	1.0	-5.2318654423	3.9382646991	0.7749609007
H	1.0	-1.6496177967	4.5494654292	1.4167016937
H	1.0	-1.5033385913	5.0127140796	-0.2871392740
H	1.0	-2.7142700480	5.7917346803	0.7423836575
O	8.0	1.4062420192	1.1794861400	1.0368403830
O	8.0	1.0874440033	0.5648234765	-1.4367610893
C	6.0	2.4749258401	0.3204025245	-1.7933190556
C	6.0	2.6435381755	0.5725955728	-3.2752306232
H	1.0	3.6782217697	0.3771879690	-3.5611991153
H	1.0	2.4071207927	1.6071375877	-3.5254648153
H	1.0	1.9971355136	-0.0844959088	-3.8578527292
H	1.0	3.1075797599	0.9835252332	-1.2027941448
H	1.0	2.7247376472	-0.7116650225	-1.5404530733

Section S10: References

1. P. A. Zaikin, O. T. Dyan, D. V. Evtushok, A. N. Usoltsev, G. I. Borodkin, E. V. Karpova and V. G. Shubin, *Eur. J. Org. Chem.*, 2017, **2017**, 2469-2474.
2. Y. Li, Z. Qi, H. Wang, X. Fu and C. Duan, *J. Org. Chem.*, 2012, **77**, 2053-2057.
3. C. Lamberth, F. M. Kessabi, R. Beaudegnies, L. Quaranta, S. Trah, G. Berthon, F. Cederbaum, T. Vettiger and C. Prasanna, *Synlett*, 2014, **25**, 858-862.
4. H. Wang, Z. Lu, S. J. Lord, W. Moerner and R. J. Twieg, *Tetrahedron lett.*, 2007, **48**, 3471-3474.
5. C. Hansch, A. Leo and R. Taft, *Chemical Reviews*, 1991, **91**, 165-195.
6. S. B. Qiu, D. D. Dai, C. L. Guo, Z. L. Sun, H. Li, X. H. Qian and Y. J. Yang, *Dyes Pigm.*, 2019, **163**, 55-61.
7. C. Li, T. Wang, N. Li, M. Li, Y. T. Li, Y. Sun, Y. Tian, J. R. Zhu, Y. Q. Wu, D. Z. Zhang and X. Y. Cui, *Chem. Comm.*, 2019, **55**, 11802-11805.
8. P. Greenspan and S. D. Fowler, *J. Lipid Res.*, 1985, **26**, 781-789.
9. A. Gandioso, R. Bresolí-Obach, A. Nin-Hill, M. Bosch, M. Palau, A. Galindo, S. Contreras, A. Rovira, C. Rovira, S. Nonell and V. Marchań, *J. Org. Chem.*, 2018, **83**, 1185-1195.
10. S. Benson, A. Fernandez, N. D. Barth, F. de Moliner, M. H. Horrocks, C. S. Herrington, J. L. Abad, A. Delgado, L. Kelly and Z. Chang, *Angew. Chem. Int. Ed.*, 2019, **131**, 6985-6989.
11. T. Lagache, N. Sauvonnet, L. Danglot and J. C. Olivo-Marin, *J. Cytometry Part A*, 2015, **87**, 568-579.
12. M. W. Schmidt, K. K. Baldridge, J. A. Boatz, S. T. Elbert, M. S. Gordon, J. H. Jensen, S. Koseki, N. Matsunaga, K. A. Nguyen, S. J. Su, T. L. Windus, M. Dupuis and J. A. Montgomery, *J. Comput. Chem.*, 1993, **14**, 1347−1363.
13. M. S. Gordon and M. W. Schmidt, in *Theory and applications of computational chemistry*, Elsevier, 2005, pp. 1167-1189.
14. T. H. Dunning Jr, *J. Chem. Phys.*, 1989, **90**, 1007-1023.
15. B. P. Prascher, D. E. Woon, K. A. Peterson, T. H. Dunning and A. K. Wilson, *Theor. Chem. Acc.*, 2011, **128**, 69-82.
16. T. H. Dunning Jr, K. A. Peterson and A. K. Wilson, *J. Chem. Phys.*, 2001, **114**, 9244-9253.
17. W. Kohn and L. J. Sham, *Phys. Rev.*, 1965, **140**, A1133.
18. E. Runge and E. K. Gross, *Phys. Rev. Lett.*, 1984, **52**, 997.
19. E. Gross and W. Kohn, in *Adv. Quantum Chem.*, Elsevier, 1990, vol. 21, pp. 255-291.
20. P. Elliott, F. Furche and K. Burke, *Rev. Comp. Ch.*, 2009, **26**, 91.
21. M. Chiba, T. Tsuneda and K. Hirao, *J. Chem. Phys.*, 2006, **124**, 144106.
22. A. D. Becke, *J. Chem. Phys.*, 1993, **98**, 5648-5652.
23. P. J. Stephens, F. Devlin, C. Chabalowski and M. J. Frisch, *J. Phys. Chem.*, 1994, **98**, 11623-11627.
24. R. H. Hertwig and W. Koch, *Chem. Phys. Lett.*, 1997, **268**, 345-351.
25. C. W. Murray, N. C. Handy and G. J. Laming, *Mol. Phys.*, 1993, **78**, 997-1014.
26. V. I. Lebedev and D. Laikov, *Dokday Mathematica,*1999, **59**, 477-481.
27. S. Miertuš, E. Scrocco and J. Tomasi, *Chem. Phys.*, 1981, **55**, 117-129.
28. H. Li and J. H. Jensen, *J. Comput. Chem.*, 2004, **25**, 1449-1462.
29. J. Tomasi, B. Mennucci and R. Cammi, *Chem. Rev.*, 2005, **105**, 2999-3094.

30. H. Li, *J. Chem. Phys.*, 2009, **131**, 184103.
31. Y. Wang and H. Li, *J. Chem. Phys.*, 2010, **133**, 034108.
32. R. S. Mulliken, *J. Chem. Phys.*, 1955, **23**, 1833-1840.
33. B. M. Bode and M. S. Gordon, *J. Mol. Graph. Model.*, 1998, **16**, 133-138.
34. R. Nakagaki, N. Kitamura, I. Aoyama and H. Ohtsubo, *J. Photch. Photbio. A*, 1994, **80**, 113-119.
35. S. Qiu, D. Dai, C. Guo, Z. Sun, H. Li, X. Qian and Y. Yang, *Dyes Pigm.*, 2019, **163**, 55-61.
36. C. J. MacNevin, T. Watanabe, M. Weitzman, A. Gulyani, S. Fuehrer, N. K. Pinkin, X. Tian, F. Liu, J. Jin and K. M. Hahn, *J. Amer. Chem. Soc.*, 2019, **141**, 7275-7282.
